# Serum profiling identifies CCL8, CXCL13, and IL-1RA as markers of active disease in patients with systemic lupus erythematosus

**DOI:** 10.3389/fimmu.2023.1257085

**Published:** 2023-11-30

**Authors:** Julius Lindblom, Lorenzo Beretta, Maria Orietta Borghi, PRECISESADS Clinical Consortium, Lorenzo Beretta, Marta E. Alarcón-Riquelme, Ioannis Parodis

**Affiliations:** ^1^ Division of Rheumatology, Department of Medicine Solna, Karolinska Institutet, Stockholm, Sweden; ^2^ Department of Gastroenterology, Dermatology and Rheumatology, Karolinska University Hospital, Stockholm, Sweden; ^3^ Referral Center for Systemic Autoimmune Diseases, Fondazione IRCCS Ca’ Granda Ospedale Maggiore Policlinico di Milano, Milan, Italy; ^4^ IRCCS Istituto Auxologico Italiano, Immunorheumatology Research Laboratory, Milan, Italy; ^5^ Department of Clinical Sciences and Community Health, University of Milan, Milan, Italy; ^6^ GENYO, Centre for Genomics and Oncological Research: Pfizer, University of Granada/Andalusian Regional Government, Medical Genomics, Granada, Spain; ^7^ Institute of Environmental Medicine, Karolinska Institutet, Stockholm, Sweden; ^8^ Department of Rheumatology, Faculty of Medicine and Health, Örebro University, Örebro, Sweden

**Keywords:** autoimmunity, systemic lupus erythematosus, antiphospholipid syndrome, diagnosis, biomarkers, cytokines, autoantibodies

## Abstract

**Introduction:**

Systemic lupus erythematosus (SLE) is a clinically heterogeneous disease that presents a challenge for clinicians. To identify potential biomarkers for diagnosis and disease activity in SLE, we investigated a selected yet broad panel of cytokines and autoantibodies in patients with SLE, healthy controls (HC), and patients with other autoimmune diseases (AIDs).

**Methods:**

Serum samples from 422 SLE patients, 546 HC, and 1223 other AIDs were analysed within the frame of the European PRECISESADS project (NTC02890121). Cytokine levels were determined using Luminex panels, and autoantibodies using different immunoassays.

**Results:**

Of the 83 cytokines analysed, 29 differed significantly between patients with SLE and HC. Specifically, CCL8, CXCL13, and IL-1RA levels were elevated in patients with active, but not inactive, SLE versus HC, as well as in patients with SLE versus other AIDs. The levels of these cytokines also correlated with SLE Disease Activity Index 2000 (SLEDAI-2K) scores, among five other cytokines. Overall, the occurrence of autoantibodies was similar across SLEDAI-2K organ domains, and the correlations between autoantibodies and activity in different organ domains were weak.

**Discussion:**

Our findings suggest that, upon validation, CCL8, CXCL13, and IL-1RA could serve as promising serum biomarkers of activity in SLE.

## Introduction

The immune system forms a complex organisation of molecules and cells that provides host defence mechanisms against infectious agents. Its importance is highlighted in autoimmune diseases, where a breach of immune tolerance leads to tissue damage through a cascade of events. Autoimmune diseases affect 3–5% of the population and contribute to greater morbidity and mortality compared with the general population (1). Among the autoimmune diseases, systemic lupus erythematosus (SLE) constitutes the prototype of systemic autoimmune disease and is characterised by diverse immunological dysfunctions that involve multiple organs. The maladaptive immune response of both the innate and the adaptive immune system in SLE leads to immune cell activation and cytokine release, ultimately resulting in autoantibody production, complement activation, inflammatory injury, and tissue damage (2). Among the many immune mediators implicated in SLE pathogenesis, type I interferons (IFNs) have been shown to play a central role in both human and animal studies (3). Administration of IFNα has been shown to accelerate disease progression in murine lupus, supporting a causative role of type I IFNs in SLE pathogenesis (4). Elevated interferon-induced cytokines and transcripts, the latter known as an interferon signature, have also been identified in the blood (5–8) and tissues (3) of patients with SLE.

The aetiology of SLE is multifactorial, involving genetic, hormonal, and environmental factors, and SLE exhibits striking clinical heterogeneity (2). The marked clinical diversity ranges from mild features, such as skin rashes and arthritis, to more severe manifestations, such as lupus nephritis (LN) and neuropsychiatric SLE (NPSLE). Non-targeted treatment for SLE includes glucocorticoids, antimalarials, and broad immunosuppressive agents. In the past two decades, further advances in treatment have been achieved with the introduction of biological therapies, such as belimumab, a monoclonal antibody against B cell activating factor belonging to the tumour necrosis factor (TNF) ligand family (BAFF), and anifrolumab that binds the type I IFN receptor, both approved for treating SLE, as well as the anti-CD20 agent rituximab that is used off-label in refractory cases (9).

The immune dysfunction in SLE causes laboratory haematological and immunological abnormalities that can be detected through routine clinical care. However, due to the heterogeneity of the disease, managing SLE remains a clinical challenge. To aid in the diagnosis of SLE, general laboratory indices and antinuclear antibodies (ANAs), especially anti-double-stranded DNA (dsDNA) and anti-Smith (Sm) antibodies, are frequently used in clinical practice (2). However, monitoring disease activity remains a considerable challenge for clinicians. Although changes in some serological markers, particularly anti-dsDNA and complement levels, may suggest a change in overall disease activity, these markers do not accurately predict flares or activity in all patients, especially organ-specific activity (2, 10, 11). Despite advances in technology with broad molecular and cellular profiling through next-generation sequencing, such biomarker screens are not yet readily implemented in routine clinical practice (12). Nevertheless, selective panels of serum biomarkers that are less invasive than tissue biomarkers might have merit in the future clinical management of SLE. Early diagnosis and early signals of impending flares, thus early initiation of treatment, are crucial for the prevention of organ damage in patients with SLE (2), and biomarkers for diagnosis and monitoring are therefore eagerly awaited (13–15).

The aim of this study was to investigate serum levels of selected cytokines and autoantibodies in patients with SLE compared with healthy controls (HC) or patients with other autoimmune diseases (AIDs) from the PRECISESADS project (6) towards identification of biomarkers for SLE diagnosis and disease activity.

## Materials and methods

### Study population and data

Patient data and peripheral blood samples were collected from individuals diagnosed with the following AIDs: SLE (N=422), systemic sclerosis (SSc; N=355), primary Sjögren’s syndrome (pSS; N=336), rheumatoid arthritis (RA; N=344), primary antiphospholipid syndrome (pAPS; N=97), and mixed connective tissue disease (MCTD; N=91). The diagnosis was based on internationally recognised diagnostic or classification criteria (16–21). We also included 546 healthy controls (HC). Data and samples from patients and controls were acquired within the 5-year European PRECISESADS project (NTC02890121; see **Supplementary Table 1** for complete inclusion and exclusion criteria). Local investigators’ affiliations can be found in the online **Supplementary Material** (page 4). Levels of a broad panel of selected cytokines and autoantibodies were measured, as described previously (6). Initially, 88 cytokines were measured in a subset of 288 patients and HC using Luminex xMAP Technology from Luminex Corporation (Austin, TX, USA). Subsequently, a customised panel from R&D Systems (Luminex assay, Luminex Corporation, Austin, TX, USA) was used to analyse 12 cytokines (C-C motif chemokine ligand 4 [CCL4], CCL8, CCL13, CCL17, chemokine (C-X-C motif) ligand 10 [CXCL10], CXCL13, Fas ligand [FasL], growth differentiation factor 15 [GDF15], interleukin 1 receptor type 2 [IL-1R2], interleukin 1 receptor antagonist [IL-1RA], matrix metalloproteinase 8 [MMP-8], and TNF receptor 1 [TNFR1]), while a quantitative sandwich enzyme immunoassay from Biorad Laboratories Inc. (Hercules, CA, USA) was employed for the analysis of 6 cytokines (BAFF, C-reactive protein [CRP], IL-6, MMP-2, transforming growth factor β [TGF-β], and TNF-α). Autoantibodies were analysed using an automated chemiluminescent immunoassay (IDS-iSYS, Immunodiagnostic Systems Holdings Ltd., East Boldon, United Kingdom). A turbidimetric immunoassay (SPAPLUS analyzer, The Binding Site Group Ltd., Birmingham, United Kingdom) was used to analyse rheumatoid factor (RF), complement component 3c (C3c), C4, and polyclonal free light chains of kappa and lambda type (PFLC), and an enzyme-linked immunosorbent assay (ELISA) kit from EUROIMMUN Medizinische Labordiagnostika AG (Lübeck, Germany) was employed for the analysis of anti-chromatin antibodies.

### Definitions of SLE disease activity

The SLE Disease Activity Index (SLEDAI) was used to evaluate the overall SLE disease activity. This index considers the physician’s overall assessment and consists of 24 descriptors, each assigned a separate score based on its specific importance (22). SLEDAI 2000 (SLEDAI-2K) is a modified version of SLEDAI that treats certain descriptors as active whenever they are present, unlike the original SLEDAI where they are only considered active upon initial occurrence or recurrence (23). In this study, the disease activity in SLE patients was evaluated using SLEDAI-2K, while clinical disease activity irrespective of serological activity was assessed using the clinical version of SLEDAI-2K (cSLEDAI-2K) (24), which excludes the serological descriptors (DNA binding and hypocomplementemia) from the SLEDAI-2K score.

### Statistical analysis

Data are presented as numbers (percentage) or means (standard deviation), and in the case of non-normal distributions the medians (interquartile range) are indicated. We compared serum levels of cytokines between AIDs and HC using the non-parametric Mann-Whitney *U* test. Inactive SLE was defined as having a SLEDAI-2K score equal to zero, while active SLE was defined as a having a SLEDAI-2K score ≥1. We also assessed correlations between cytokine levels, and in relation to SLEDAI-2K scores, using the Spearman’s rank correlation coefficient (ρ). Autoantibody positivity and low levels of C3c and C4 in relation to activity in distinct organ domains, as defined by the cSLEDAI-2K, were reported in the form of chord diagrams using the circlize R package (25), and correlations were assessed using the phi correlation coefficient (φ). We also assessed correlations between serological markers and history of or current organ manifestations of SLE, as per the case report form (CRF) of the PRECISESADS study protocol or as determined by the SLEDAI-2K organ domains, using the phi correlation coefficient (φ). Specifications regarding numbers of individuals in each group being subjected to comparative analysis are provided in the corresponding **Supplementary Tables**. *p* values <0.05 were considered statistically significant. Analyses were performed using the R software version 4.2.1 (R Foundation for Statistical Computing, Vienna, Austria).

## Results

Patient characteristics and clinical data are presented in **Table 1**. The majority of patients with SLE were women (92.4%). The mean age of the patients was 45.9 years (standard deviation [s.d.]: ± 13.8 years) at the time of enrolment. The age was similar in the other groups (HC, pAPS, and AIDs), except for patients with AIDs whose mean age was 56.8 ± 13.3 years. The mean SLEDAI-2K score was 6.1 ± 5.9 years. Among patients with SLE, 116 (33.5%) tested positive for anti-dsDNA and 114 (32.4%) had low levels of C4. A total of 317 (75.1%) SLE patients received antimalarial agents.

**Table 1:** Characteristics of patients with SLE, patients with pAPS, patients with autoimmune diseases other than SLE, and healthy controls from the PRECISESADS study population.

			Comparators	
	SLE	HC	pAPS	AID*
	N=422	N=546	N=97	N=1223
Demographics				
**Age (years); mean (s.d.)**	45.9 ± 13.8	46.8 ± 13.1	48.3 ± 12.0	56.8 ± 13.3
**Female sex; n (%)**	390 (92.4%)	429 (78.6%)	70 (72.2%)	1035 (84.6%)
Clinical data				
**Disease duration (years); mean (s.d.)**	13.9 ± 9.7	N/A	9.9 ± 7.8	11.2 ± 9.0
**SLEDAI-2K score; mean (s.d.)**	6.2 ± 5.9; N=393	N/A	N/A	N/A
Serological profile; n (%)				
**Anti-dsDNA (+)**	116 (33.5%); N=346	N/A	4 (6.5%); N=62	32 (3.1%); N=1038
**Anti-Sm (+)**	17 (5.3%); N=320	N/A	0 (0.0%); N=62	21 (2.1%); N=995
**Anti-β_2_GPI IgG (+)**	38 (11.1%); N=343	N/A	32 (52.5%); N=61	38 (3.7%); N=1037
**Anti-β_2_GPI IgM (+)**	27 (7.9%); N=343	N/A	12 (19.4%); N=62	56 (5.4%); N=1038
**aCL IgG (+)**	39 (11.3%); N=346	N/A	32 (51.6%); N=62	41 (3.9%); N=1038
**aCL IgM (+)**	32 (9.2%); N=346	N/A	15 (24.2%); N=62	66 (6.4%); N=1038
**Low C3c**	138 (39.2%); N=352	N/A	12 (19.4%); N=62	260 (25.0%); N=1042
**Low C4**	114 (32.4%); N=352	N/A	10 (16.1%); N=62	154 (14.8%); N=1042
Medications				
**Prednisone equivalent dose during** **follow-up (mg/day); median (IQR)**	1.0 (0.0–5.0); N=411	N/A	0.0 (0.0–0.0); N=93	0.0 (0.0–2.0); N=1159
**Antimalarial agents; n (%)**	317 (75.1%)	N/A	23 (23.7%)	264 (21.6%)
**Immunosuppressants^†^; n (%)**	200 (47.4%)	N/A	4 (4.1%)	443 (36.2%)
**Biologics^‡^; n (%)**	4 (1.4%)	N/A	0 (0.0%)	173 (14.1%)

Data are presented as numbers (percentage) or means ± standard deviation. In case of non-normal distributions, the medians (interquartile range) are indicated. In case of missing values, the total number of patients with available data is indicated. aCL, antibodies against cardiolipin; AID, autoimmune disease; anti-β_2_GPI, antibodies against β_2_-glycoprotein I; anti-dsDNA, antibodies against double-stranded DNA; anti-Sm, antibodies against Smith; C3c, complement component 3c; C4, complement component 4; HC, healthy controls; Ig, immunoglobulin; IQR, interquartile range; N/A, not applicable; pAPS, primary antiphospholipid syndrome; s.d., standard deviation; SLE, systemic lupus erythematosus; SLEDAI-2K, Systemic Lupus Erythematosus Disease Activity Index 2000. *Mixed connective tissue disease, primary antiphospholipid syndrome, primary Sjögren’s syndrome, rheumatoid arthritis, or systemic sclerosis.^†^Azathioprine, calcineurin inhibitors, cyclophosphamide, leflunomide, methotrexate, or mycophenolic acid. ^‡^Abatacept, belimumab, interleukin (IL)-6 inhibitors, rituximab, or tumour necrosis factor (TNF) inhibitors.

### Cytokine profiles in SLE, other autoimmune diseases, and HC

**Figure 1:**
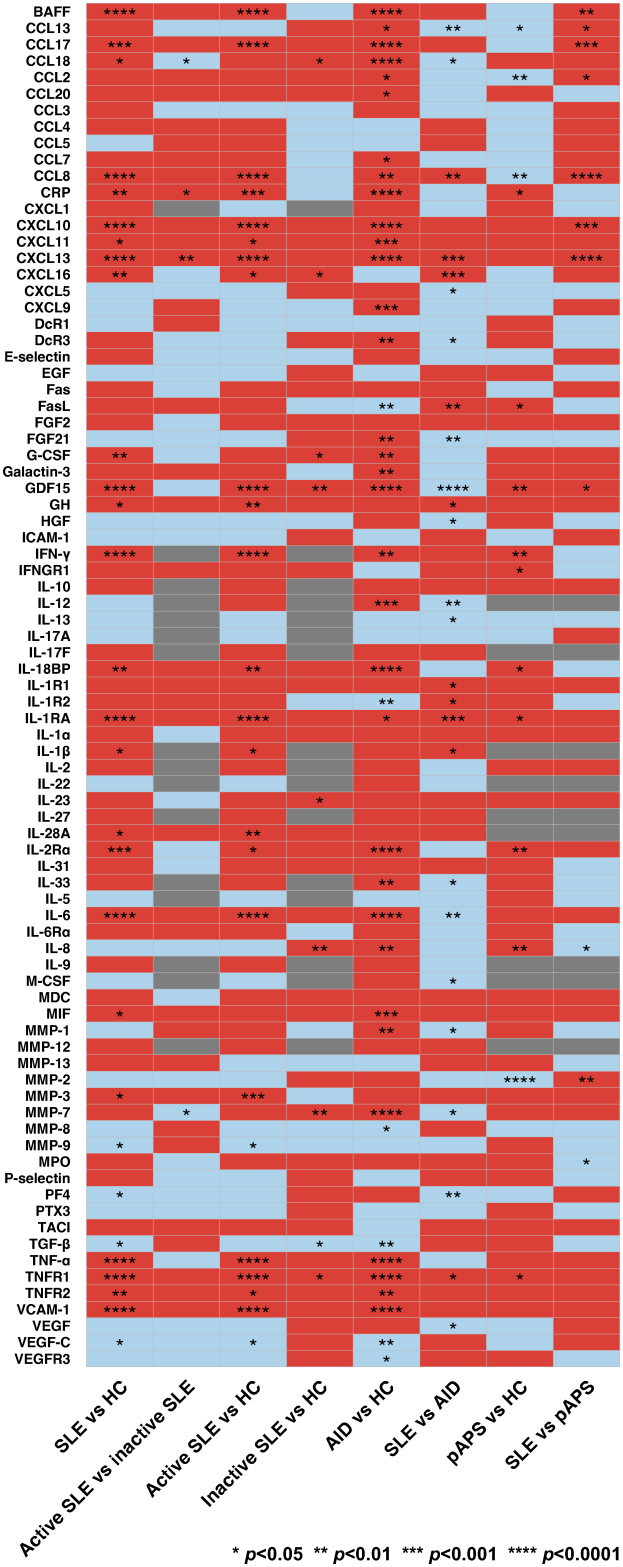
Cytokine levels in patients with systemic lupus erythematosus (SLE), healthy controls (HC), and patients with other autoimmune diseases (AIDs). Comparator groups included SLE, including active and inactive SLE, primary antiphospholipid syndrome (pAPS), AIDs other than SLE (mixed connective tissue disease, pAPS, primary Sjögren’s syndrome, rheumatoid arthritis, or systemic sclerosis), and HC. The latter group for each comparison was used as reference. Red and blue colours denote upregulation and downregulation of cytokines, respectively. Comparisons with insufficient observations are indicated in grey. p values are derived from Mann-Whitney *U* tests. Statistically significant differences are denoted by asterisks. AID, autoimmune disease; BAFF, B cell activating factor belonging to the tumour necrosis factor ligand family; CCL13, C-C motif chemokine ligand 13; CCL17, C-C motif chemokine ligand 17; CCL18; C-C motif chemokine ligand 18; CCL2; C-C motif chemokine ligand 2; CCL20; C-C motif chemokine ligand 20; CCL3; C-C motif chemokine ligand 3; CCL4; C-C motif chemokine ligand 4; CCL5; C-C motif chemokine ligand 5; CCL7; C-C motif chemokine ligand 7; CCL8; C-C motif chemokine ligand 8; CRP, C-reactive protein, CXCL1, C-X-C motif ligand 1; CXCL10, C-X-C motif ligand 10; CXCL11, C-X-C motif ligand 11; CXCL13, C-X-C motif ligand 13, CXCL16, C-X-C motif ligand 16; CXCL5, C-X-C motif ligand 5; CXCL9, C-X-C motif ligand 9; DcR1, decoy receptor 1; DcR3, decoy receptor 3; EGF, epidermal growth factor; FasL; Fas ligand; FGF2, fibroblast growth factor 2; FGF21, fibroblast growth factor 21; G-CSF, granulocyte colony-stimulating factor; GDF15, growth differentiation factor 15; GH, growth hormone; HC, healthy controls; HGF, hepatocyte growth factor; ICAM-1, intercellular adhesion molecule 1; IFN-γ, interferon γ; IFNGR1, interferon γ receptor 1; IL-10, interleukin 10; IL-12, interleukin 12; IL-13, interleukin 13; IL-17A, interleukin 17A; IL-17F, interleukin 17F; IL-18BP, interleukin 18 binding protein; IL-1R1, interleukin 1 receptor type 1; IL-1R2, interleukin 1 receptor type 2; IL-1RA, interleukin 1 receptor antagonist; IL-1α, interleukin 1α; IL-1β, interleukin 1β; IL-2, interleukin 2; IL-22, interleukin 22; IL-23, interleukin 23; IL-27, interleukin 27; IL-28A, interleukin 28A; IL-2Rα, interleukin 2 receptor α; IL-31, interleukin 31; IL-33, interleukin 33; IL-5, interleukin 5; IL-6, interleukin 6; IL-6Rα, interleukin 6 receptor α; IL-8, interleukin 8; IL-9; interleukin 9; M-CSF, macrophage colony-stimulating factor; MDC, macrophage-derived chemokine; MIF, macrophage migration inhibitory factor; MMP-1, matrix metalloproteinase 1; MMP-12, matrix metalloproteinase 12; MMP-13, matrix metalloproteinase 13; MMP-2, matrix metalloproteinase 12; MMP-3, matrix metalloproteinase 3; MMP-7, matrix metalloproteinase 7; MMP-8, matrix metalloproteinase 8; MMP-9, matrix metalloproteinase 9; MPO, myeloperoxidase; pAPS, primary antiphospholipid syndrome; PF4, platelet factor 4; PTX3, pentraxin 3; SLE, systemic lupus erythematosus; TACI, transmembrane activator and calcium modulator and cyclophilin ligand interactor; TGF-β, transforming growth factor β; TNF-α, tumour necrosis factor α; TNFR1, tumour necrosis factor receptor 1; TNFR2, tumour necrosis factor receptor 2; VCAM-1, vascular cell adhesion protein 1; VEGF, vascular endothelial growth factor; VEGF-C, vascular endothelial growth factor C; VEGFR3, vascular endothelial growth factor receptor 3.

Of the 83 cytokines that were analysed, 29 differed significantly between patients with SLE and HC (**Figure 1**; **Supplementary Table 2**). Among these, only serum levels of CRP and CXCL13 were higher in active versus inactive SLE (median [interquartile range]: 6.19 [5.75–6.59] pg/mL versus 5.79 [5.31–6.18] pg/mL; *p*=0.036 and 2.03 [1.88–2.27] pg/mL versus 5.79 [1.75–1.99] pg/mL; *p*=0.004; **Supplementary Table 3**). However, 21 cytokines differed significantly between patients with active, but not inactive, SLE and HC, including CRP (6.19 [5.75–6.59] pg/mL versus 5.97 [5.31–6.18] pg/mL; *p*=0.001) and CXCL13 (2.03 [1.88–2.27] pg/mL versus 1.78 [1.69–1.89] pg/mL; *p*<0.001; **Supplementary Tables 4, 5**).

A total of 39 cytokines differed significantly between patients with AIDs other than SLE (MCTD, pAPS, pSS, RA, or SSc) and HC (**Supplementary Table 6**). Of the 33 cytokines that were elevated in patients with AIDs other than SLE and HC, the serum levels of four cytokines were even higher in patients with SLE compared with patients with other AIDs (**Supplementary Table 7**). The latter cytokines included CCL8 (1.92 [1.76–2.05] pg/mL versus 1.88 [1.73–2.01] pg/mL; *p*=0.007), CXCL13 (2.02 [1.85–2.24] pg/mL versus 1.95 (1.78–2.10) pg/mL; *p*<0.001), IL-1RA (3.01 [2.89–3.19] pg/mL versus 2.96 (2.82–3.12) pg/mL; *p*=0.001), and TNFR1 (3.72 [3.62–3.81] pg/mL versus 3.69 (3.69–3.79) pg/mL; *p*=0.029). Furthermore, 10 cytokines differed significantly between SLE and other AIDs but not between patients with AIDs other than SLE and HC (**Supplementary Tables 6, 7**).

In total, 14 cytokines differed significantly between patients with pAPS and HC, including 10 that were elevated in pAPS (**Supplementary Table 8**). Among the latter, only GDF15 was even higher in patients with SLE than that in patients with pAPS (2.89 [2.70–3.11] pg/mL versus 2.74 (2.67–2.98) pg/mL; *p*=0.036; **Supplementary Table 9**). Five cytokines differed significantly between SLE and pAPS, but not between patients with pAPS and HC (**Supplementary Tables 8, 9**). Levels of 16 cytokines differed significantly between pAPS and AIDs other than SLE and pAPS (**Supplementary Table 10**).

### Correlation of autoimmunity-related cytokines with SLE disease activity

**Figure 2:**
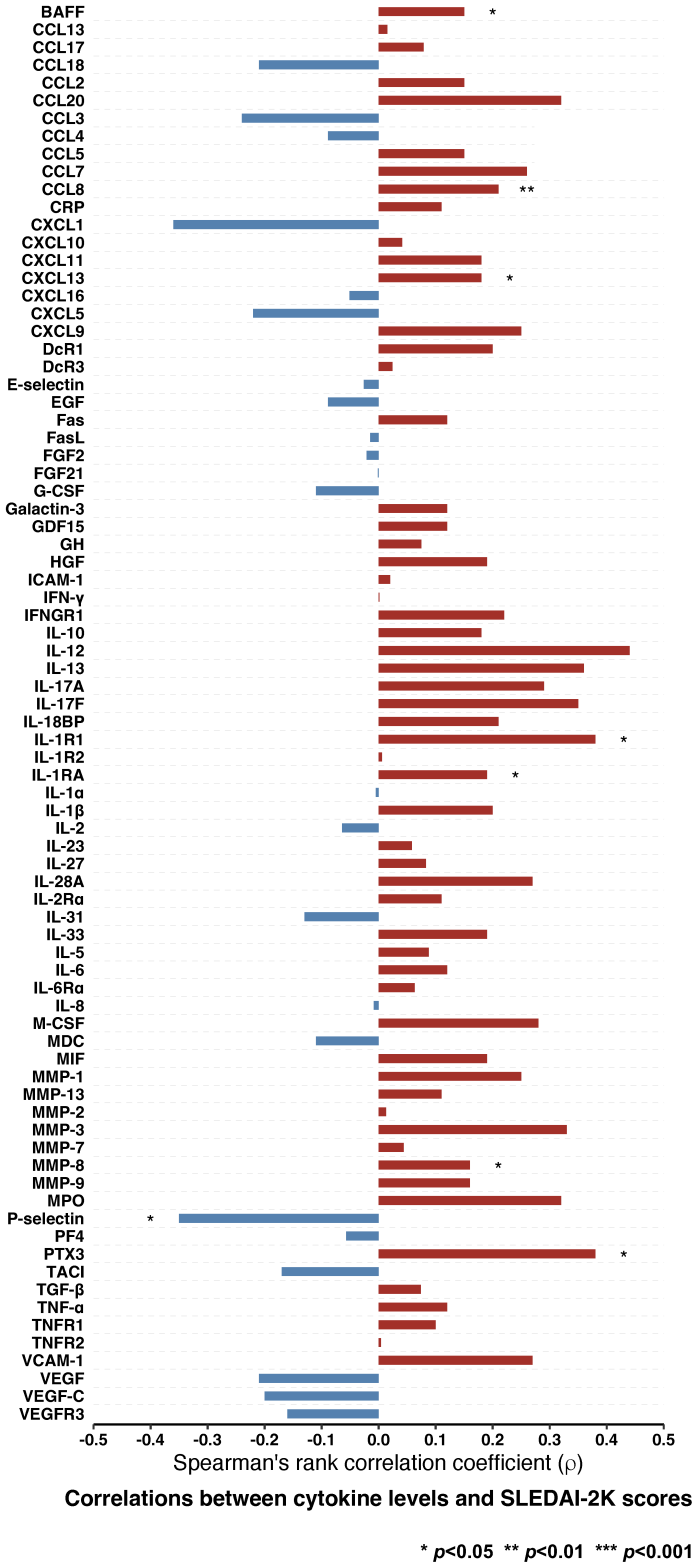
Correlations between cytokine levels and disease activity in patients with SLE. Spearman’s rank correlations between levels of different cytokines and SLEDAI-2K scores. Statistically significant correlations are denoted by asterisks. BAFF, B cell activating factor belonging to the tumour necrosis factor ligand family; CCL13, C-C motif chemokine ligand 13; CCL17, C-C motif chemokine ligand 17; CCL18; C-C motif chemokine ligand 18; CCL2; C-C motif chemokine ligand 2; CCL20; C-C motif chemokine ligand 20; CCL3; C-C motif chemokine ligand 3; CCL4; C-C motif chemokine ligand 4; CCL5; C-C motif chemokine ligand 5; CCL7; C-C motif chemokine ligand 7; CCL8; C-C motif chemokine ligand 8; CRP, C-reactive protein, CXCL1, C-X-C motif ligand 1; CXCL10, C-X-C motif ligand 10; CXCL11, C-X-C motif ligand 11; CXCL13, C-X-C motif ligand 13, CXCL16, C-X-C motif ligand 16; CXCL5, C-X-C motif ligand 5; CXCL9, C-X-C motif ligand 9; DcR1, decoy receptor 1; DcR3, decoy receptor 3; EGF, epidermal growth factor; FasL; Fas ligand; FGF2, fibroblast growth factor 2; FGF21, fibroblast growth factor 21; G-CSF, granulocyte colony-stimulating factor; GDF15, growth differentiation factor 15; GH, growth hormone; HGF, hepatocyte growth factor; ICAM-1, intercellular adhesion molecule 1; IFN-γ, interferon γ; IFNGR1, interferon γ receptor 1; IL-10, interleukin 10; IL-12, interleukin 12; IL-13, interleukin 13; IL-17A, interleukin 17A; IL-17F, interleukin 17F; IL-18BP, interleukin 18 binding protein; IL-1R1, interleukin 1 receptor type 1; IL-1R2, interleukin 1 receptor type 2; IL-1RA, interleukin 1 receptor antagonist; IL-1α, interleukin 1α; IL-1β, interleukin 1β; IL-2, interleukin 2; IL-23, interleukin 23; IL-27, interleukin 27; IL-28A, interleukin 28A; IL-2Rα, interleukin 2 receptor α; IL-31, interleukin 31; IL-33, interleukin 33; IL-5, interleukin 5; IL-6, interleukin 6; IL-6Rα, interleukin 6 receptor α; IL-8, interleukin 8; M-CSF, macrophage colony-stimulating factor; MDC, macrophage-derived chemokine; MIF, macrophage migration inhibitory factor; MMP-1, matrix metalloproteinase 1; MMP-13, matrix metalloproteinase 13; MMP-2, matrix metalloproteinase 12; MMP-3, matrix metalloproteinase 3; MMP-7, matrix metalloproteinase 7; MMP-8, matrix metalloproteinase 8; MMP-9, matrix metalloproteinase 9; MPO, myeloperoxidase; PF4, platelet factor 4; PTX3, pentraxin 3; SLE, systemic lupus erythematosus; SLEDAI-2K, Systemic Lupus Erythematosus Disease Activity Index 2000; TACI, transmembrane activator and calcium modulator and cyclophilin ligand interactor; TGF-β, transforming growth factor β; TNF-α, tumour necrosis factor α; TNFR1, tumour necrosis factor receptor 1; TNFR2, tumour necrosis factor receptor 2; VCAM-1, vascular cell adhesion protein 1; VEGF, vascular endothelial growth factor; VEGF-C, vascular endothelial growth factor C; VEGFR3, vascular endothelial growth factor receptor 3.

Albeit weak, correlations between eight cytokines and SLEDAI-2K scores were found to be statistically significant. Serum levels of P-selectin were negatively correlated with SLEDAI-2K scores (ρ=-0.35, *p*=0.040), whereas levels of seven other cytokines were positively correlated (**Figure 2**; **Supplementary Table 11**). Those included BAFF (ρ=0.15, *p*=0.019), CCL8 (ρ=0.21, *p*=0.005), CXCL13 (ρ=0.18, *p*=0.021), IL-1R1 (ρ=0.38, *p*=0.023), IL-1RA (ρ=0.19, *p*=0.015), MMP-8 (ρ=0.16, *p*=0.035), and pentraxin 3 (PTX-3; ρ=0.38, *p*=0.023).

### CCL8, CXCL13, and IL-1RA in SLE versus other autoimmune diseases or HC

**Figure 3:**
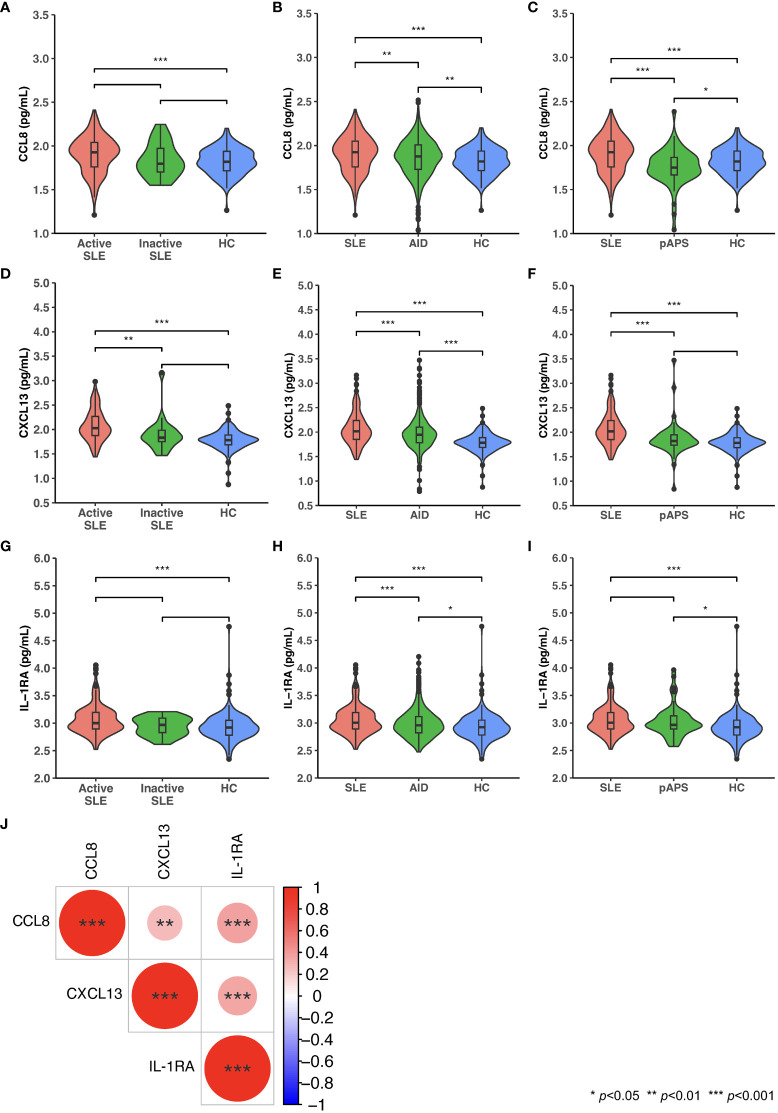
Serum levels of CCL8, CXCL13, and IL-1RA in patients with SLE, patients with other autoimmune diseases (AIDs), and healthy controls (HC). Violin plots displaying distributions of circulating levels of CCL8 **(A-C)**, CXCL13 **(D-F)**, and IL-1RA **(G-I)** in patients with active versus inactive SLE versus HC **(A, D, G)**, patients with SLE versus patients with AID versus HC **(B, E, H)**, and patients with SLE versus patients with primary antiphospholipid syndrome (pAPS) versus HC **(C, F, I)**. **(J)** Spearman’s rank correlations between levels of CCL8, CXCL13, and IL-1RA. Statistically significant correlations are denoted by asterisks. AID: autoimmune disease; CCL8; C-C motif chemokine ligand 8; CXCL13: C-X-C motif ligand 13: IL-1RA: interleukin 1 receptor antagonist; pAPS: primary antiphospholipid syndrome; SLE: systemic lupus erythematosus.

CCL8, CXCL13, and IL-1RA levels were elevated in patients with active, but not inactive, SLE versus HC, as well as in patients with SLE versus other AIDs, and the levels of these cytokines correlated with SLE disease activity. Distributions of levels of these cytokines in patients with SLE, active SLE, inactive SLE, other AIDs, and pAPS, and in HC are shown in **Figures 3A-I**. In patients with SLE, levels of CCL8 and levels of IL-1RA displayed the strongest albeit weak correlation (ρ=0.37, *p*<0.001), followed by CXCL13 and IL-1RA levels (ρ=0.34, *p*<0.001), and CCL8 and CXCL13 levels (ρ=0.28, *p*=0.003), as illustrated in **Figure 3J**.

### Autoantibody profiles across SLE manifestations

**Figure 4:**
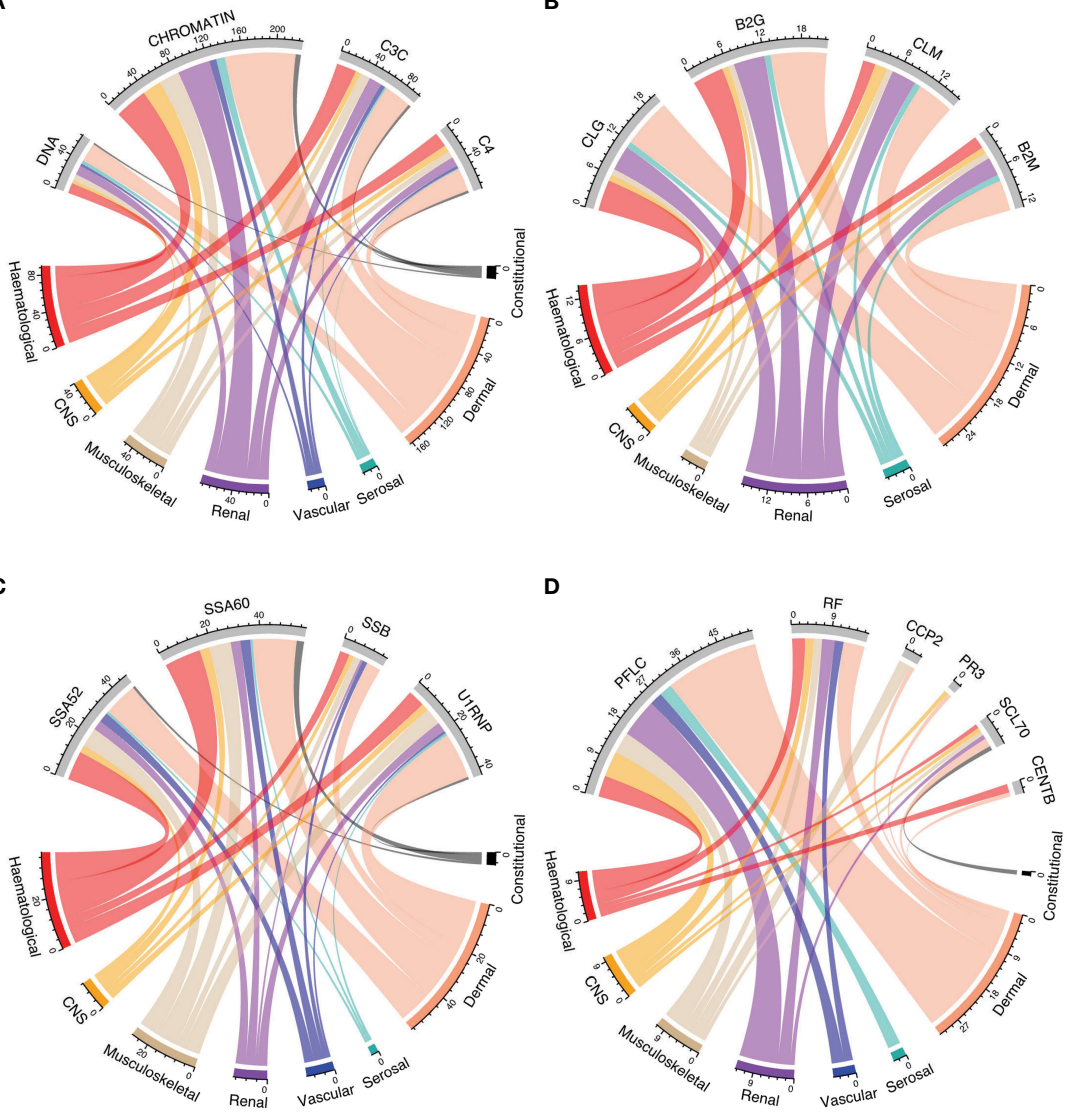
Autoantibody positivity and low levels of C3c and C4 in relation to organ domain-specific activity based on the cSLEDAI-2K. Chord diagrams of autoantibody levels and levels of C3c and C4 in relation to activity in cSLEDAI-2K organ domains, stratified into groups: **(A)** anti-dsDNA, anti-chromatin, complement C3, and complement C4; **(B)** anti-cardiolipin IgG, anti-β2GPI IgG, anti-cardiolipin IgM, and anti-β2GPI IgM; **(C)** anti-SSA/Ro52, anti-SSA/Ro60, anti-SSB/La, and anti-U1-RNP; **(D)** polyclonal kappa and lambda, rheumatoid factor, anti-CCP2, anti-PR3, anti-Scl70, and anti-centromere **(B)** B2G, anti-β2 glycoprotein I IgG; B2M, anti-β2 glycoprotein I IgM; C3c, complement component 3c; C4, complement component 4; CCP2, anti-cyclic citrullinated peptide (second generation); CLG, anti-cardiolipin IgG; CLM, anti-cardiolipin IgM; cSLEDAI-2K, clinical Systemic Lupus Erythematosus Disease Activity Index 2000; DNA, anti-double stranded (ds)DNA; PFLC, polyclonal free light chains of kappa and lambda type; PR3, anti-proteinase 3; RF, rheumatoid factor; SSA52, anti-SSA/Ro52; SSA60, anti-SSA/Ro60; SSB, anti-SSB/La.

**Figure 5:**
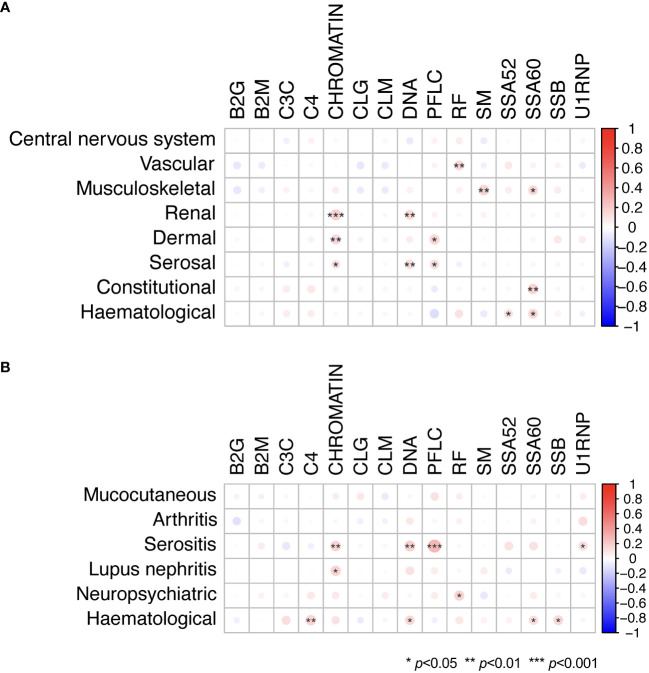
Serological markers in relation to organ domain-specific activity based on the cSLEDAI-2K, and history of or current involvement of various organ systems. Phi (φ) correlations between autoantibody levels and levels of C3c and C4, and **(A)** activity in cSLEDAI-2K organ domains and **(B)** history of or current involvement of various organ systems in patients with SLE. Mucocutaneous manifestations included malar rash, photosensitivity, acute cutaneous lupus erythematosus, discoid lupus, chronic cutaneous lupus erythematosus, mucosal ulcers, and alopecia. Haematological manifestations included haemolytic anaemia, leukopenia, lymphopenia, and thrombocytopenia. Statistically significant correlations are denoted by asterisks. B2G, anti-β2 glycoprotein I IgG; B2M, anti-β2 glycoprotein I IgM; C3c, complement component 3c; C4, complement component 4; CCP2, anti-cyclic citrullinated peptide (second generation); CLG, anti-cardiolipin IgG; CLM, anti-cardiolipin IgM; cSLEDAI-2K, clinical Systemic Lupus Erythematosus Disease Activity Index 2000; DNA, anti-double stranded (ds)DNA; PFLC, polyclonal free light chains of kappa and lambda type; RF, rheumatoid factor; SM, anti-Smith; SSA52, anti-SSA/Ro52; SSA60, anti-SSA/Ro60; SSB, anti-SSB/La.

**Figures 4A-D** shows the relationship between positivity for selected autoantibodies, low levels of C3c, or C4 and different clinical manifestations as determined by the cSLEDAI-2K organ domains. Overall, the occurrence of serological markers was similar across cSLEDAI-2K organ domains, and correlations between serological markers and activity in the different cSLEDAI-2K organ domains were weak (**Figure 5A**; **Supplementary Tables 12–19**). The strongest correlations, although still weak, were observed between anti-Sm antibody positivity and disease activity in the SLEDAI-2K musculoskeletal domain (φ=0.17, *p*=0.002), anti-chromatin antibody positivity and disease activity in the SLEDAI-2K renal domain (φ=0.19, *p*=0.001), and anti-SSA/Ro52 antibody positivity and fever as per SLEDAI-2K (φ=0.17, *p*=0.004).

Overall weak correlations were also seen for serological markers in relation to history of or current involvement of various organ systems, as per the CRF of the PRECISESADS study protocol or as determined by the cSLEDAI-2K organ domains (**Figure 5B**; **Supplementary Tables 20–25**). Notably, a correlation was seen between high PFLC and history of or current serositis (φ=0.28, *p*<0.001), as well as a correlation between anti-dsDNA antibody positivity and history of or current serositis (φ=0.18, *p*=0.003).

## Discussion

In the present study, we investigated serum levels of autoimmunity-related cytokines and autoantibodies in patients with SLE compared with HC or patients with other AIDs. We found that several IFN-inducible proteins were elevated in SLE patients compared with HC. Additionally, serum levels of CCL8, CXCL13, and IL-1RA were higher in patients with SLE compared with patients with other AIDs, and they correlated with global SLE disease activity. The occurrence of routine serological markers was in general similar across organ manifestations of SLE, and correlations between markers and organ-specific disease activity were weak.

Among other markers, serum levels of CCL8 (also known as monocyte chemoattractant protein 2 [MCP-2]) were elevated in patients with SLE compared with HC. CCL8 is an IFN-inducible protein involved in the chemotaxis of T cells and dendritic cells and has previously been reported to be elevated in SLE patients compared with HC (5). Furthermore, the gene expression of CCL8 has been reported to be higher in bone marrow mesenchymal stem cells from patients with SLE than in cells from HC (26). We also found CCL8 levels to be elevated in patients with SLE compared with other AIDs, including pAPS when analysed separately, which suggests disease specificity for this chemokine. Moreover, CCL8 levels were higher in SLE patients with active disease, but not in patients with inactive disease, compared with HC, and the levels of this cytokine correlated with global SLE disease activity. Similarly, urine CCL8 has previously been reported to be elevated in active LN patients compared with non-LN SLE patients (27). However, a study by Petrackova et al. using a proximity extension immunoassay (PEA) did not detect a difference in serum levels of in SLE patients compared with HC, nor any relationship between serum CCL8 levels and SLEDAI (28). Thus, while our findings and other evidence point to an SLE-specific signal, further validation studies are needed to confirm the clinical utility of CCL8 in patients with SLE.

The B cell-attracting chemokine CXCL13 exhibited a similar pattern to CCL8 in patients with SLE compared with controls and in relation to SLE disease activity, which is consistent with previous literature (29). CXCL13 has been implicated in the pathogenesis of several autoimmune diseases and even suggested as a potential therapeutic target for autoimmune disorders, including SLE (29, 30). It was recently found to be associated with an inflammatory cluster in an integrative analysis of whole-blood transcriptome and methylome data in patients with systemic autoimmune diseases (6). In the case of SLE, CXCL13 has been shown to cause B cell infiltration in kidneys, worsening murine LN (31). Moreover, *in vitro* experiments have demonstrated that CXCL13 stimulation triggers a proinflammatory response in human podocytes (32). While CXCL13 is a stable serum marker (33), it is worth noting that it can also be induced by bacterial exposure (30).

Interferon signalling is of known importance in SLE pathogenesis (3). We herein identified several IFN-inducible proteins that were elevated in SLE patients compared with HC, including BAFF, CCL8, CXCL10, and CXCL11, consistent with previous findings (5). Moreover, serum BAFF levels have previously been reported to be associated with an interferon cluster in an integrative analysis of transcriptome and methylome data in patients with systemic autoimmune diseases (6), as well as an interferon gene module in a whole-blood transcriptome analysis in patients with SLE (8). Notably, the addition of the anti-type I IFN receptor monoclonal anifrolumab to standard therapy yielded greater suppression of elevated serum levels of the aforementioned cytokines in active SLE patients (SLEDAI-2K scores ≥10) than placebo in the phase II MUSE (NCT01438489) trial (34, 35). Similarly, addition of deucravacitinib, a selective tyrosine kinase 2 (TYK2) inhibitor, but not addition of placebo to standard therapy was recently shown to reduce serum levels of BAFF, CCL8, and CXCL10 in patients with active SLE in the phase II PAISLEY (NCT03252587) trial (36, 37).

Serum levels of the anti-inflammatory cytokine IL-1RA were herein found to be elevated in patients with SLE compared with HC or patients with other AIDs. Additionally, IL-1RA levels showed the strongest, albeit moderate, correlation with global SLE disease activity. Elevated serum levels of IL-1RA have been reported in various rheumatic diseases, including SLE (38), and were recently also associated with an inflammatory pattern based on gene expression and methylome data across seven different autoimmune disorders (6). A study by Suzuki et al. reported serum IL-1RA levels to be higher in active SLE patients compared with HC or patients with other rheumatic diseases (39). Furthermore, genetic studies have shown an association between IL-1RA gene polymorphisms and susceptibility to SLE in European and Asian populations (40). A mass cytometry analysis by O’Gorman et al. revealed a distinct monocyte signature with increased levels of IL-1RA in newly diagnosed and untreated paediatric SLE patients compared with HC (41). However, the potential correlation between IL-1RA and global SLE disease activity remains controversial due to conflicting reports (39, 42–46). Although recombinant IL-1RA (anakinra) has shown efficacy in patients with RA (47), data in SLE are scarce. Notably, small studies have shown effectiveness of IL-1 inhibition to alleviate arthritis in patients with SLE (48, 49), supporting the anti-inflammatory role of IL-1RA.

Occurrence of selected autoantibodies and low levels of C3c or C4 were similar across different organ manifestations of SLE, and correlations between these serological markers and organ-specific disease activity were weak. These findings support the notion that serological markers typically used in routine clinical practice have limited ability to assess disease activity in SLE, particularly organ-specific activity (50). However, Lewis et al. identified autoantibody clusters associated with different patterns of organ involvement and a panel of 26 autoantibodies that improved the diagnostic accuracy of SLE when using an advanced protein microarray that screened for 1543 different autoantibodies (51). In the present study, we also found weak correlations for serological markers in relation to the history of or current involvement of various organ systems. Our findings suggest that autoantibodies commonly used in routine clinical practice may serve as stable disease markers rather than sensitive indicators of the disease course in patients with SLE.

PRECISESADS is a multicentre project with participants of European origin, which presumably limits the generalisability of the results to non-European populations. Analyses were not adjusted for multiple testing due to the limited numbers of study participants; results should therefore be interpreted with caution. Numbers of patients across treatment groups limited us from accounting for treatments in analyses. Additionally, the cross-sectional design of the study precluded the analysis of changes in serological markers over time and in response to treatment, which nevertheless was beyond the scope of the present study. Nonetheless, the overall large sample size and the inclusion of patients with SLE, HC, and other AIDs constituted significant strengths of the study. Further validation of CCL8, CXCL13, and IL-1RA as biomarkers in SLE across multiple cohorts and diverse populations is warranted, as is standardisation of their quantification and establishment of appropriate cut-offs.

To conclude, analysis of a broad selection of autoimmunity-related cytokines and autoantibodies measured in serum revealed that several IFN-inducible proteins were elevated in SLE patients compared with HC. Furthermore, serum levels of CCL8, CXCL13, and IL-1RA were also higher in patients with SLE compared with patients with other autoimmune diseases and were moderately correlated with global SLE disease activity. Our findings suggest that, upon validation in other cohorts, CCL8, CXCL13, and IL-1RA may have merit as useful serum biomarkers of activity in SLE. Further investigation of the dynamics of these biomarkers over time is warranted to establish their utility in tracking disease activity in patients with SLE and in guiding therapeutic decisions.

## Data availability statement

Raw data is the property of the PRECISESADS consortium and is protected under the European General Data Protection Regulation (GDPR). Metadata and aggregated processed data are available upon reasonable request from the corresponding author and from the EGA (European Genome-phenome Archive).

## Ethics statement

The study complied with the ethical principles of the Declaration of Helsinki. Written informed consent was obtained from all study participants prior to enrolment in the PRECISESADS project. The PRECISESADS project was approved by regional ethics review boards for all participating centres, and the study protocol for the present analysis was reviewed and approved by the Swedish Ethical Review Authority (2022-03907-01).

## Author contributions

JL: Conceptualization, Formal Analysis, Methodology, Visualization, Writing – original draft, Writing – review & editing. LB: Resources, Writing – review & editing. MB: Resources, Writing – review & editing. MA-R: Funding acquisition, Resources, Writing – review & editing. IP: Conceptualization, Funding acquisition, Methodology, Resources, Supervision, Writing – original draft, Writing – review & editing.

## PRECISESADS clinical consortium

Lorenzo Beretta, Barbara Vigone, Jacques-Olivier Pers, Alain Saraux, Valérie Devauchelle-Pensec, Divi Cornec, Sandrine Jousse-Joulin, Bernard Lauwerys, Julie Ducreux, Anne-Lise Maudoux, Carlos Vasconcelos, Ana Tavares, Esmeralda Neves, Raquel Faria, Mariana Brandão, Ana Campar, António Marinho, Fátima Farinha, Isabel Almeida, Miguel Angel Gonzalez-Gay Mantecón, Ricardo Blanco Alonso, Alfonso Corrales Martínez, Ricard Cervera, Ignasi Rodríguez-Pintó, Gerard Espinosa, Rik Lories, Ellen De Langhe, Nicolas Hunzelmann, Doreen Belz, Torsten Witte, Niklas Baerlecken, Georg Stummvoll, Michael Zauner, Michaela Lehner, Eduardo Collantes, Rafaela Ortega-Castro, M^a^Angeles Aguirre-Zamorano, Alejandro Escudero-Contreras, M^a^Carmen Castro-Villegas, Norberto Ortego, María Concepción Fernández Roldán, Enrique Raya, Inmaculada Jiménez Moleón, Enrique de Ramon, Isabel Díaz Quintero, Pier Luigi Meroni, Maria Gerosa, Tommaso Schioppo, Carolina Artusi, Carlo Chizzolini, Aleksandra Zuber, Donatienne Wynar, Laszló Kovács, Attila Balog, Magdolna Deák, Márta Bocskai, Sonja Dulic, Gabriella Kádár, Falk Hiepe, Velia Gerl, Silvia Thiel, Manuel Rodriguez Maresca, Antonio López-Berrio, Rocío Aguilar-Quesada, and Héctor Navarro-Linares.
