# Supplementary material for: Serum profiling identifies CCL8, CXCL13, and IL-1RA as markers of active disease in patients with systemic lupus erythematosus
Source: Front Immunol. 2023 Nov 30;14:1257085. doi: 10.3389/fimmu.2023.1257085 (PMC10720584; doi:10.3389/fimmu.2023.1257085)
Supplement: Supplementary file 1 [file DataSheet_1.pdf]

Supplementary Material

Table of contents

1 Supplementary Table 1. Main inclusion and exclusion criteria 3

2 List of local investigators from the participating clinical sites..4

3 Supplementary Table 2. Serum levels of selected autoimmunity-associated cytokines in patients with SLE versus healthy controls in the PRECISESADS study population 5

4 Supplementary Table 3. Serum levels of selected autoimmunity-associated cytokines in patients with active versus inactive SLE in the PRECISESADS study population 7

5 Supplementary Table 4. Serum levels of selected autoimmunity-associated cytokines in patients with active SLE versus healthy controls in the PRECISESADS study population 9

6 Supplementary Table 5. Serum levels of selected autoimmunity-associated cytokines in patients with inactive SLE versus healthy controls in the PRECISESADS study population 11

7 Supplementary Table 6. Serum levels of selected autoimmunity-associated cytokines in patients with autoimmune diseases* versus healthy controls in the PRECISESADS study population 13

8 Supplementary Table 7. Serum levels of selected autoimmunity-associated cytokines in patients with SLE versus other autoimmune diseases* in the PRECISESADS study population 16

9 Supplementary Table 8. Serum levels of selected autoimmunity-associated cytokines in patients with pAPS versus healthy controls in the PRECISESADS study population.....................................19

10 Supplementary Table 9. Serum levels of selected autoimmunity-associated cytokines in patients with SLE versus pAPS in the PRECISESADS study population..21

11 Supplementary Table 10. Serum levels of selected autoimmunity-associated cytokines in patients with pAPS versus other autoimmune diseases* in the PRECISESADS study population.....23

12 Supplementary Table 11. Correlations between cytokine levels and disease activity in patients with SLE..26

13 Supplementary Table 12. Correlations between positivity for selected serological markers and the cSLEDAI-2K CNS domain 28

14 Supplementary Table 13. Correlations between positivity for selected serological markers and the cSLEDAI-2K vascular domain...29

15 Supplementary Table 14. Correlations between positivity for selected serological markers and the cSLEDAI-2K musculoskeletal domain 30

16 Supplementary Table 15. Correlations between positivity for selected serological markers and the cSLEDAI-2K renal domain 31

17 Supplementary Table 16. Correlations between positivity for selected serological markers and the cSLEDAI-2K mucocutaneous domain 32

18 Supplementary Table 17. Correlations between positivity for selected serological markers and the cSLEDAI-2K serosal domain 33

19 Supplementary Table 18. Correlations between positivity for selected serological markers and the cSLEDAI-2K constitutional domain 34

20 Supplementary Table 19. Correlations between positivity for selected serological markers and the cSLEDAI-2K haematological domain..35

21 Supplementary Table 20. Correlations between positivity for selected serological markers and history of or current mucocutaneous manifestations 36

22 Supplementary Table 21. Correlations between positivity for selected serological markers and history of or current arthritis...37

23 Supplementary Table 22. Correlations between positivity for selected serological markers and history of or current serositis 38

24 Supplementary Table 23. Correlations between positivity for selected serological markers and history of or current lupus nephritis 39

25 Supplementary Table 24. Correlations between positivity for selected serological markers and history of or current neuropsychiatric systemic lupus erythematosus...40

26 Supplementary Table 25. Correlations between positivity for selected serological markers and history of or current haematological manifestations 41

Supplementary Table 1. Main inclusion and exclusion criteria

Inclusion criteria	Exclusion criteria
**For all patients**	**For all patients**
- Age ≥18 years<br>- Diagnosed according to the prevailing criteria for systemic lupus erythematosus (SLE)<br>- Informed consent signed	- Pediatric lupus<br>- Drug-induced lupus<br>- Severe nephrotic syndrome with proteinuria ≥3.5 g/day<br>- Patients with stable doses of prednisone equivalent >15 mg/day for the last 3 months or with IV corticosteroids in the last 3 months<br>- Patients under immunosuppressant treatment in the last 3 months prior to recruitment and patients with combined therapy using two or more immunosuppressants:<br>• Methotrexate ≥25mg/week<br>• Azathioprine ≥2.5mg/kg/day<br>• Cyclosporine A >3mg/kg/day<br>• Mycophenolate mofetil >2g/day<br>- Chronic HBV or HCV infection<br>- Patients who are also diagnosed according to the prevailing criteria for one of the following autoimmune diseases:<br>• Rheumatoid arthritis (RA)<br>• Scleroderma or systemic sclerosis (SSc)<br>• Primary Sjögren’s syndrome (pSjS)<br>• Primary antiphospholipid syndrome (pAPS)<br>• Mixed connective tissue disease (MCTD)<br>• Patients with undifferentiated connective tissue disease (UCTD) for over 1 year and that do not fulfill the diagnosis of any of the above diseases or SLE
	**For controls**
	- Individuals on chronic medication<br>- Individuals suffering from any inflammatory autoimmune, allergic or infectious condition, and with a history of autoimmune disease, particularly thyroid disease or other diseases that may modify cellular profiles in blood

Adapted from “Integrative Analysis Reveals a Molecular Stratification of Systemic Autoimmune Diseases.” Arthritis Rheum. 2021;73(6):1073-1085. Copyright 2021 by the American College of Rheumatology. Adapted with permission. IV: intravenous, HBV: hepatitis B virus, HCV: hepatitis C virus.

2 List of local investigators from the participating clinical sites

Lorenzo Beretta[1], Barbara Vigone[1], Jacques-Olivier Pers[2], Alain Saraux[2], Valérie Devauchelle-Pensec[2], Divi Cornec[2], Sandrine Jousse-Joulin[2], Bernard Lauwerys[3], Julie Ducreux[3], Anne-Lise Maudoux[3], Carlos Vasconcelos[4], Ana Tavares[4], Esmeralda Neves[4], Raquel Faria[4], Mariana Brandão[4], Ana Campar[4], António Marinho[4], Fátima Farinha[4], Isabel Almeida[4], Miguel Angel Gonzalez-Gay Mantecón[5], Ricardo Blanco Alonso[5], Alfonso Corrales Martínez[5], Ricard Cervera[6], Ignasi Rodríguez-Pintó[6], Gerard Espinosa[6], Rik Lories[7], Ellen De Langhe[7], Nicolas Hunzelmann[8], Doreen Belz[8], Torsten Witte[9], Niklas Baerlecken[9], Georg Stummvoll[10], Michael Zauner[10], Michaela Lehner[10], Eduardo Collantes[11], Rafaela Ortega-Castro[11], Mª Angeles Aguirre-Zamorano[11], Alejandro Escudero-Contreras[11], Mª Carmen Castro-Villegas[11], Norberto Ortego[12], María Concepción Fernández Roldán[12], Enrique Raya[13], Inmaculada Jiménez Moleón[13], Enrique de Ramon[14], Isabel Díaz Quintero[14], Pier Luigi Meroni[15], Maria Gerosa[15], Tommaso Schioppo[15], Carolina Artusi[15], Carlo Chizzolini[16], Aleksandra Zuber[16], Donatienne Wynar[16], Laszló Kovács[17], Attila Balog[17], Magdolna Deák[17], Márta Bocskai[17], Sonja Dulic[17], Gabriella Kádár[17], Falk Hiepe[18], Velia Gerl[18], Silvia Thiel[18], Manuel Rodriguez Maresca[19], Antonio López-Berrio[19], Rocío Aguilar-Quesada[19], Héctor Navarro-Linares[19]

Affiliations

1. Referral Center for Systemic Autoimmune Diseases, Fondazione IRCCS Ca' Granda Ospedale Maggiore Policlinico di Milano, Italy.
2. Centre Hospitalier Universitaire de Brest, Hospital de la Cavale Blanche, Brest, France.
3. Pôle de pathologies rhumatismales systémiques et inflammatoires, Institut de Recherche Expérimentale et Clinique, Université catholique de Louvain, Brussels, Belgium.
4. Centro Hospitalar do Porto, Portugal.
5. Servicio Cantabro de Salud, Hospital Universitario Marqués de Valdecilla, Santander, Spain.
6. Hospital Clinic I Provicia, Institut d'Investigacions Biomèdiques August Pi i Sunyer, Barcelona, Spain.
7. Katholieke Universiteit Leuven, Belgium.
8. Klinikum der Universitaet zu Koeln, Cologne, Germany.
9. Medizinische Hochschule Hannover, Germany.
10. Medical University Vienna, Vienna, Austria.
11. Servicio Andaluz de Salud, Hospital Universitario Reina Sofía Córdoba, Spain.
12. Servicio Andaluz de Salud, Complejo hospitalario Universitario de Granada (Hospital Universitario San Cecilio), Spain.
13. Servicio Andaluz de Salud, Complejo hospitalario Universitario de Granada (Hospital Virgen de las Nieves), Spain.
14. Servicio Andaluz de Salud, Hospital Regional Universitario de Málaga, Spain
15. Università degli studi di Milano, Milan, Italy.
16. Hospitaux Universitaires de Genève, Switzerland.
17. University of Szeged, Szeged, Hungary.
18. Charite, Berlin, Germany.
19. Andalusian Public Health System Biobank, Granada, Spain

3 **Supplementary Table 2.** Serum levels of selected autoimmunity-associated cytokines in patients with SLE versus healthy controls in the PRECISESADS study population

**Cytokines**	**SLE**		**HC**		***p* value**
	**n/N**	**Serum levels (pg/mL)**	**n/N**	**Serum levels (pg/mL)**	
**BAFF**	258/422	6.04 (5.84–6.17)	295/546	5.97 (5.87–6.05)	**<0.001**
**CCL13**	194/422	2.16 (2.01–2.27)	178/546	2.15 (2.04–2.26)	0.608
**CCL17**	194/422	2.80 (2.62–3.02)	178/546	2.71 (2.50–2.87)	**<0.001**
**CCL18**	45/422	4.60 (4.49–4.68)	48/546	4.49 (4.38–4.55)	**0.018**
**CCL2**	45/422	2.65 (2.50–2.71)	48/546	2.61 (2.55–2.70)	0.851
**CCL20**	45/422	1.68 (1.48–1.86)	48/546	1.58 (1.46–1.78)	0.267
**CCL3**	38/422	2.31 (2.15–2.47)	42/546	2.30 (2.13–2.46)	0.992
**CCL4**	184/422	2.23 (2.08–2.49)	160/546	2.20 (1.95–2.52)	0.229
**CCL5**	45/422	4.67 (4.46–4.81)	48/546	4.69 (4.55–4.84)	0.569
**CCL7**	44/422	1.99 (1.81–2.30)	45/546	1.93 (1.62–2.21)	0.188
**CCL8**	194/422	1.92 (1.76–2.05)	178/546	1.82 (1.72–1.94)	**<0.001**
**CRP**	162/422	6.16 (5.61–6.57)	143/546	5.97 (5.42–6.36)	**0.005**
**CXCL1**	25/422	2.09 (1.94–2.20)	27/546	2.04 (1.91–2.30)	0.956
**CXCL10**	194/422	1.45 (1.29–1.77)	178/546	1.28 (1.13–1.40)	**<0.001**
**CXCL11**	45/422	2.46 (2.29–2.55)	48/546	2.36 (2.27–2.46)	**0.017**
**CXCL13**	194/422	2.02 (1.85–2.24)	175/546	1.78 (1.69–1.89)	**<0.001**
**CXCL16**	45/422	3.11 (3.04–3.19)	48/546	3.07 (3.02–3.10)	**0.004**
**CXCL5**	45/422	3.06 (2.87–3.27)	48/546	3.15 (3.04–3.38)	0.124
**CXCL9**	45/422	2.78 (2.67–3.00)	47/546	2.78 (2.67–2.86)	0.125
**DcR1**	45/422	3.47 (3.33–3.64)	48/546	3.53 (3.39–3.60)	0.643
**DcR3**	45/422	2.64 (2.49–2.73)	47/546	2.64 (2.48–2.75)	0.582
**E-selectin**	45/422	4.40 (4.26–4.49)	48/546	4.38 (4.21–4.50)	0.845
**EGF**	45/422	2.27 (2.06–2.44)	48/546	2.30 (2.04–2.39)	0.906
**Fas**	45/422	3.89 (3.83–4.02)	48/546	3.86 (3.82–3.90)	0.059
**FasL**	194/422	1.65 (1.49–1.78)	178/546	1.62 (1.54–1.73)	0.628
**FGF2**	45/422	2.18 (2.06–2.26)	48/546	2.12 (2.06–2.24)	0.389
**FGF21**	44/422	3.33 (3.18–3.45)	48/546	3.35 (3.17–3.46)	0.897
**G-CSF**	45/422	1.48 (1.41–1.55)	47/546	1.39 (1.31–1.46)	**0.003**
**Galactin-3**	45/422	3.89 (3.79–3.98)	48/546	3.85 (3.78–3.91)	0.113
**GDF15**	194/422	2.89 (2.70–3.11)	178/546	2.70 (2.57–2.84)	**<0.001**
**GH**	45/422	3.43 (2.96–3.62)	48/546	3.02 (2.61–3.42)	**0.034**
**HGF**	45/422	2.26 (2.14–2.41)	48/546	2.32 (2.23–2.40)	0.385
**ICAM-1**	45/422	5.39 (5.24–5.61)	48/546	5.40 (5.18–5.52)	0.498
**IFN-γ**	32/422	26.09 (21.99–32.58)	39/546	17.51 (13.79–20.56)	**<0.001**
**IFNGR1**	45/422	1.88 (1.84–2.00)	48/546	1.88 (1.79–1.92)	0.070
**IL-10**	23/422	0.54 (0.22–1.12)	15/546	0.28 (0.19–0.56)	0.244
**IL-12**	13/422	10.70 (6.58–28.37)	19/546	14.81 (6.11–30.33)	0.939
**IL-13**	22/422	2.21 (2.05–2.30)	24/546	2.33 (1.89–2.44)	0.261
**IL-17A**	18/422	1.94 (0.62–3.35)	15/546	2.99 (1.86–4.99)	0.158
**IL-17F**	18/422	1.11 (0.76–1.52)	12/546	0.94 (0.25–1.18)	0.305
**IL-18BP**	45/422	2.43 (2.32–2.64)	48/546	2.28 (2.05–2.44)	**0.002**
**IL-1α**	45/422	1.61 (1.44–1.72)	48/546	1.52 (1.38–1.78)	0.258
**IL-1β**	25/422	4.49 (3.21–7.15)	31/546	3.17 (1.89–4.81)	**0.015**
**IL-1R1**	45/422	3.17 (3.06–3.23)	48/546	3.11 (3.07–3.16)	0.194
**IL-1R2**	194/422	3.84 (3.74–3.90)	178/546	3.83 (3.77–3.89)	0.896
**IL-1RA**	192/422	3.01 (2.89–3.19)	178/546	2.92 (2.78–3.05)	**<0.001**
**IL-2**	32/422	1.98 (1.82–2.09)	28/546	1.88 (1.66–2.06)	0.159
**IL-22**	7/422	1.24 (0.96–1.30)	7/546	1.31 (0.84–1.46)	0.620
**IL-23**	45/422	2.61 (2.50–2.71)	48/546	2.56 (2.45–2.65)	0.257
**IL-27**	21/422	185.83 (99.39–281.72)	18/546	117.50 (48.72–188.24)	0.088
**IL-28A**	26/422	2.38 (1.86–2.68)	27/546	2.05 (1.80–2.16)	**0.027**
**IL-2Rα**	45/422	2.72 (2.62–2.89)	48/546	2.63 (2.53–2.72)	**<0.001**

**IL-31**	38/422	2.10 (2.02–2.17)	43/546	2.06 (1.93–2.21)	0.508
**IL-33**	29/422	3.48 (2.19–5.21)	33/546	3.07 (2.25–5.80)	0.767
**IL-5**	32/422	3.72 (3.10–4.16)	38/546	3.90 (3.47–4.50)	0.179
**IL-6**	256/422	3.33 (2.90–3.60)	290/546	3.18 (2.91–3.38)	**<0.001**
**IL-6Rα**	45/422	4.08 (4.01–4.11)	48/546	4.07 (4.03–4.12)	0.948
**IL-8**	45/422	1.28 (1.16–1.41)	48/546	1.29 (1.20–1.36)	0.531
**IL-9**	7/422	302.42 (194.84–529.25)	6/546	263.29 (52.12–558.71)	0.720
**M-CSF**	13/422	55.62 (31.77–67.73)	16/546	58.42 (29.66–166.84)	0.380
**MDC**	45/422	2.87 (2.70–2.96)	48/546	2.83 (2.70–2.91)	0.292
**MIF**	44/422	3.79 (3.55–3.95)	48/546	3.60 (3.44–3.83)	**0.020**
**MMP-1**	45/422	3.29 (3.02–3.49)	48/546	3.30 (3.12–3.45)	1.000
**MMP-12**	5/422	107.31 (98.79–130.23)	7/546	53.62 (35.45–91.67)	0.415
**MMP-13**	41/422	2.58 (2.39–2.73)	40/546	2.54 (2.35–2.60)	0.221
**MMP-2**	164/422	5.33 (5.26–5.42)	143/546	5.35 (5.27–5.44)	0.344
**MMP-3**	45/422	4.22 (3.99–4.45)	47/546	4.09 (3.98–4.19)	**0.021**
**MMP-7**	45/422	3.40 (3.24–3.55)	48/546	3.32 (3.18–3.41)	0.078
**MMP-8**	193/422	3.51 (3.26–3.78)	178/546	3.57 (3.35–3.80)	0.125
**MMP-9**	45/422	5.28 (5.02–5.44)	48/546	5.35 (5.24–5.47)	**0.037**
**MPO**	45/422	5.12 (4.81–5.29)	48/546	5.08 (4.95–5.32)	0.881
**P-selectin**	45/422	4.60 (4.54–4.68)	48/546	4.60 (4.55–4.66)	0.985
**PF4**	44/422	6.87 (6.79–6.97)	45/546	6.92 (6.87–7.05)	**0.016**
**PTX3**	45/422	2.97 (2.80–3.33)	48/546	3.04 (2.92–3.22)	0.758
**TACI**	43/422	1.20 (0.92–1.28)	45/546	1.04 (0.91–1.20)	0.167
**TGF-β**	258/422	8.01 (7.78–8.15)	295/546	8.07 (7.71–8.20)	**0.015**
**TNF-α**	258/422	2.95 (2.63–3.15)	294/546	2.86 (2.63–2.98)	**<0.001**
**TNFR1**	194/422	3.72 (3.62–3.81)	178/546	3.63 (3.57–3.71)	**<0.001**
**TNFR2**	45/422	3.40 (3.19–3.58)	48/546	3.32 (3.18–3.36)	**0.005**
**VCAM-1**	45/422	6.05 (5.95–6.16)	48/546	5.86 (5.79–5.94)	**<0.001**
**VEGF**	45/422	1.77 (1.65–2.00)	48/546	1.84 (1.72–2.10)	0.081
**VEGF-C**	41/422	3.00 (2.80–3.16)	47/546	3.14 (2.92–3.26)	**0.041**
**VEGFR3**	45/422	2.14 (1.75–2.36)	48/546	2.14 (1.86–2.39)	0.513

Data are presented as medians (interquartile range). In case of missing values, the total number of patients with available data is indicated. All *p* values are derived from non-parametrical Mann-Whitney *U* tests. Statistically significant *p* values are in bold. N/A indicates that the comparison could not be conducted owing to low numbers of observations (n<4).

BAFF: B cell activating factor belonging to the tumour necrosis factor ligand family; CCL13: C-C motif chemokine ligand 13; CCL17: C-C motif chemokine ligand 17; CCL18; C-C motif chemokine ligand 18; CCL2; C-C motif chemokine ligand 2; CCL20; C-C motif chemokine ligand 20; CCL3; C-C motif chemokine ligand 3; CCL4; C-C motif chemokine ligand 4; CCL5; C-C motif chemokine ligand 5; CCL7; C-C motif chemokine ligand 7; CCL8; C-C motif chemokine ligand 8; CRP: C-reactive protein: CXCL1: C-X-C motif ligand 1; CXCL10: C-X-C motif ligand 10; CXCL11: C-X-C motif ligand 11; CXCL13: C-X-C motif ligand 13: CXCL16: C-X-C motif ligand 16; CXCL5: C-X-C motif ligand 5; CXCL9: C-X-C motif ligand 9; DcR1: decoy receptor 1; DcR3: decoy receptor 3; EGF: epidermal growth factor; FasL; Fas ligand; FGF2: fibroblast growth factor 2; FGF21: fibroblast growth factor 21; G-CSF: granulocyte colony-stimulating factor; GDF15: growth differentiation factor 15; GH: growth hormone; HC: healthy controls; HGF: hepatocyte growth factor; ICAM-1: intercellular adhesion molecule 1; IFN-γ: interferon gamma; IFNGR1: interferon gamma receptor 1; IL-10: interleukin 10; IL-12: interleukin 12; IL-13: interleukin 13; IL-17A: interleukin 17A; IL-17F: interleukin 17F; IL-18BP: interleukin 18 binding protein; IL-1α: interleukin 1 α; IL-1β: interleukin 1β; IL-1R1: interleukin 1 receptor type 1; IL-1R2: interleukin 1 receptor type 2; IL-1RA: interleukin 1 receptor antagonist; IL-2: interleukin 2; IL-22: interleukin 22; IL-23: interleukin 23; IL-27: interleukin 27; IL-28A: interleukin 28A; IL-2Rα: interleukin 2 receptor α; IL-31: interleukin 31; IL-33: interleukin 33; IL-5: interleukin 5; IL-6: interleukin 6; IL-6Rα: interleukin 6 receptor α; IL-8: interleukin 8; IL-9; interleukin 9; M-CSF: macrophage colony-stimulating factor; MDC: macrophage-derived chemokine; MIF: macrophage migration inhibitory factor; MMP-1: matrix metalloproteinase 1; MMP-12: matrix metalloproteinase 12; MMP-13: matrix metalloproteinase 13; MMP-2: matrix metalloproteinase 12; MMP-3: matrix metalloproteinase 3; MMP-7: matrix metalloproteinase 7; MMP-8: matrix metalloproteinase 8; MMP-9: matrix metalloproteinase 9; MPO: myeloperoxidase; PF4: platelet factor 4; PTX3: pentraxin 3; SLE: systemic lupus erythematosus; TACI: transmembrane activator and calcium modulator and cyclophilin ligand interactor; TGF-β: transforming growth factor β; TNF-α: tumour necrosis factor α; TNFR1: tumour necrosis factor receptor 1; TNFR2: tumour necrosis factor receptor 2; VCAM-1: vascular cell adhesion protein 1; VEGF: vascular endothelial growth factor; VEGF-C: vascular endothelial growth factor C; VEGFR3: vascular endothelial growth factor receptor 3.

Supplementary Table 3. Serum levels of selected autoimmunity-associated cytokines in patients with active versus inactive SLE in the PRECISESADS study population

Cytokines	Active SLE		Inactive SLE		*p* value
	n/N	Serum levels (pg/mL)	n/N	Serum levels (pg/mL)	
**BAFF**	230/348	6.05 (5.88–6.17)	21/45	5.90 (5.81–6.10)	0.059
**CCL13**	148/348	2.15 (2.02–2.28)	20/45	2.17 (2.02–2.31)	0.858
**CCL17**	148/348	2.81 (2.62–3.02)	20/45	2.75 (2.59–2.87)	0.374
**CCL18**	31/348	4.59 (4.38–4.64)	4/45	4.71 (4.69–4.73)	**0.027**
**CCL2**	31/348	2.67 (2.51–2.72)	4/45	2.62 (2.55–2.74)	0.980
**CCL20**	31/348	1.68 (1.48–1.88)	4/45	1.64 (1.58–1.72)	0.897
**CCL3**	25/348	2.27 (2.14–2.41)	4/45	2.30 (2.22–2.37)	0.874
**CCL4**	141/348	2.23 (2.08–2.44)	19/45	2.17 (2.09–2.39)	0.525
**CCL5**	31/348	4.72 (4.46–4.87)	4/45	4.61 (4.56–4.68)	0.531
**CCL7**	30/348	1.99 (1.82–2.23)	4/45	1.81 (1.73–1.94)	0.349
**CCL8**	148/348	1.93 (1.76–2.04)	20/45	1.80 (1.70–1.97)	0.102
**CRP**	130/348	6.19 (5.75–6.59)	16/45	5.79 (5.31–6.18)	**0.036**
**CXCL1**	17/348	2.01 (1.83–2.20)	2/45	2.19 (2.18–2.19)	N/A
**CXCL10**	148/348	1.45 (1.28–1.77)	20/45	1.33 (1.19–1.51)	0.069
**CXCL11**	31/348	2.46 (2.32–2.54)	4/45	2.40 (2.34–2.43)	0.337
**CXCL13**	148/348	2.03 (1.88–2.27)	20/45	1.83 (1.75–1.99)	**0.004**
**CXCL16**	31/348	3.11 (3.05–3.17)	4/45	3.18 (3.14–3.21)	0.352
**CXCL5**	31/348	3.07 (2.90–3.28)	4/45	3.27 (3.10–3.42)	0.326
**CXCL9**	31/348	2.78 (2.68–2.96)	4/45	2.64 (2.59–2.76)	0.276
**DcR1**	31/348	3.45 (3.30–3.68)	4/45	3.42 (3.33–3.53)	0.940
**DcR3**	31/348	2.63 (2.48–2.73)	4/45	2.66 (2.48–2.80)	0.876
**E-selectin**	31/348	4.37 (4.26–4.46)	4/45	4.37 (4.24–4.48)	1.000
**EGF**	31/348	2.17 (1.93–2.41)	4/45	2.37 (2.30–2.45)	0.254
**Fas**	31/348	3.89 (3.81–4.01)	4/45	3.93 (3.88–3.99)	0.565
**FasL**	148/348	1.67 (1.47–1.79)	20/45	1.59 (1.51–1.64)	0.175
**FGF2**	31/348	2.16 (2.05–2.28)	4/45	2.21 (2.18–2.24)	0.717
**FGF21**	30/348	3.31 (3.01–3.40)	4/45	3.39 (3.31–3.49)	0.262
**G-CSF**	31/348	1.46 (1.36–1.54)	4/45	1.56 (1.50–1.62)	0.102
**Galactin-3**	31/348	3.91 (3.77–3.99)	4/45	3.85 (3.81–3.90)	0.671
**GDF15**	148/348	2.86 (2.70–3.11)	20/45	2.91 (2.64–3.16)	0.881
**GH**	31/348	3.49 (3.15–3.70)	4/45	3.29 (2.99–3.46)	0.301
**HGF**	31/348	2.25 (2.14–2.40)	4/45	2.31 (2.08–2.61)	0.708
**ICAM-1**	31/348	5.39 (5.25–5.66)	4/45	5.47 (5.32–5.63)	0.940
**IFN-γ**	29/348	26.00 (21.99–32.86)	3/45	30.96 (26.61–31.72)	N/A
**IFNGR1**	31/348	1.91 (1.83–2.00)	4/45	1.88 (1.87–1.96)	0.783
**IL-10**	21/348	0.54 (0.17–1.20)	2/45	0.74 (0.59–0.89)	N/A
**IL-12**	12/348	15.91 (6.58–28.55)	1/45	0.18 (0.18–0.18)	N/A
**IL-13**	15/348	2.20 (2.01–2.30)	3/45	2.12 (2.00–2.17)	N/A
**IL-17A**	16/348	1.94 (0.80–3.33)	2/45	2.04 (1.06–3.01)	N/A
**IL-17F**	15/348	1.30 (0.81–1.56)	2/45	0.92 (0.79–1.05)	N/A
**IL-18BP**	31/348	2.41 (2.32–2.60)	4/45	2.32 (2.08–2.56)	0.708
**IL-1α**	31/348	1.57 (1.47–1.85)	4/45	1.69 (1.62–1.72)	0.531
**IL-1β**	22/348	4.79 (2.78–7.64)	3/45	3.75 (3.49–3.88)	N/A
**IL-1R1**	31/348	3.17 (3.03–3.24)	4/45	3.17 (3.10–3.21)	0.940
**IL-1R2**	148/348	3.84 (3.75–3.91)	20/45	3.83 (3.76–3.90)	0.908
**IL-1RA**	146/348	3.00 (2.89–3.20)	20/45	2.97 (2.83–3.09)	0.053
**IL-2**	19/348	1.98 (1.80–2.08)	3/45	1.95 (1.83–2.02)	N/A
**IL-22**	5/348	1.24 (0.69–1.24)	1/45	1.28 (1.28–1.28)	N/A
**IL-23**	31/348	2.56 (2.42–2.68)	4/45	2.74 (2.69–2.75)	0.055
**IL-27**	19/348	180.41 (82.90–283.65)	2/45	243.83 (231.24–256.42)	N/A
**IL-28A**	17/348	2.51 (2.12–2.75)	4/45	2.13 (1.84–2.44)	0.244
**IL-2Rα**	31/348	2.70 (2.60–2.87)	4/45	2.73 (2.66–2.93)	0.783

**IL-31**	25/348	2.09 (2.00–2.13)	4/45	2.13 (2.01–2.24)	0.448
**IL-33**	26/348	3.64 (2.34–5.13)	3/45	3.48 (2.66–4.92)	N/A
**IL-5**	29/348	3.70 (2.97–4.16)	3/45	3.75 (3.72–3.96)	N/A
**IL-6**	228/348	3.34 (2.91–3.64)	21/45	3.20 (3.03–3.49)	0.141
**IL-6Rα**	31/348	4.07 (4.01–4.10)	4/45	4.06 (4.02–4.11)	1.000
**IL-8**	31/348	1.26 (1.13–1.40)	4/45	1.42 (1.40–1.52)	0.052
**IL-9**	6/348	287.00 (156.47–405.85)	1/45	736.41 (736.41–736.41)	N/A
**M-CSF**	10/348	55.82 (22.96–65.87)	3/45	55.62 (46.33–63.61)	N/A
**MDC**	31/348	2.87 (2.70–2.93)	4/45	2.87 (2.80–2.96)	0.635
**MIF**	30/348	3.75 (3.51–3.88)	4/45	3.70 (3.53–3.92)	0.777
**MMP-1**	31/348	3.31 (3.12–3.47)	4/45	3.23 (3.14–3.34)	0.783
**MMP-12**	5/348	107.31 (98.79–130.23)	0/45	N/A	N/A
**MMP-13**	28/348	2.53 (2.39–2.73)	4/45	2.53 (2.38–2.65)	0.588
**MMP-2**	131/348	5.33 (5.25–5.44)	17/45	5.36 (5.32–5.41)	0.200
**MMP-3**	31/348	4.29 (4.10–4.48)	4/45	3.95 (3.92–4.09)	0.064
**MMP-7**	31/348	3.37 (3.13–3.50)	4/45	3.63 (3.57–3.68)	**0.049**
**MMP-8**	147/348	3.53 (3.30–3.78)	20/45	3.47 (3.37–3.63)	0.596
**MMP-9**	31/348	5.25 (5.01–5.43)	4/45	5.22 (5.04–5.43)	0.861
**MPO**	31/348	5.11 (4.80–5.22)	4/45	5.13 (4.87–5.33)	0.980
**P-selectin**	31/348	4.58 (4.53–4.67)	4/45	4.70 (4.57–4.81)	0.233
**PF4**	30/348	6.87 (6.80–6.98)	4/45	6.93 (6.88–6.98)	0.661
**PTX3**	31/348	2.93 (2.82–3.27)	4/45	3.07 (2.81–3.35)	0.856
**TACI**	29/348	1.21 (0.97–1.28)	4/45	1.18 (1.01–1.39)	0.651
**TGF-β**	230/348	8.03 (7.83–8.15)	21/45	7.96 (7.78–8.05)	0.197
**TNF-α**	230/348	2.95 (2.67–3.16)	21/45	2.96 (2.57–3.05)	0.124
**TNFR1**	148/348	3.72 (3.62–3.81)	20/45	3.68 (3.62–3.81)	0.605
**TNFR2**	31/348	3.41 (3.22–3.58)	4/45	3.39 (3.30–3.53)	1.000
**VCAM-1**	31/348	6.04 (5.95–6.08)	4/45	5.92 (5.80–6.12)	0.499
**VEGF**	31/348	1.72 (1.63–1.99)	4/45	1.88 (1.72–2.03)	0.352
**VEGF-C**	28/348	2.95 (2.79–3.08)	4/45	3.17 (3.11–3.20)	0.093
**VEGFR3**	31/348	2.14 (1.81–2.35)	4/45	2.38 (2.18–2.47)	0.337

Data are presented as medians (interquartile range). In case of missing values, the total number of patients with available data is indicated. All *p* values are derived from non-parametrical Mann-Whitney *U* tests. Statistically significant *p* values are in bold. N/A indicates that the comparison could not be conducted owing to low numbers of observations (n<4).

BAFF: B cell activating factor belonging to the tumour necrosis factor ligand family; CCL13: C-C motif chemokine ligand 13; CCL17: C-C motif chemokine ligand 17; CCL18; C-C motif chemokine ligand 18; CCL2; C-C motif chemokine ligand 2; CCL20; C-C motif chemokine ligand 20; CCL3; C-C motif chemokine ligand 3; CCL4; C-C motif chemokine ligand 4; CCL5; C-C motif chemokine ligand 5; CCL7; C-C motif chemokine ligand 7; CCL8; C-C motif chemokine ligand 8; CRP: C-reactive protein: CXCL1: C-X-C motif ligand 1; CXCL10: C-X-C motif ligand 10; CXCL11: C-X-C motif ligand 11; CXCL13: C-X-C motif ligand 13: CXCL16: C-X-C motif ligand 16; CXCL5: C-X-C motif ligand 5; CXCL9: C-X-C motif ligand 9; DcR1: decoy receptor 1; DcR3: decoy receptor 3; EGF: epidermal growth factor; FasL; Fas ligand; FGF2: fibroblast growth factor 2; FGF21: fibroblast growth factor 21; G-CSF: granulocyte colony-stimulating factor; GDF15: growth differentiation factor 15; GH: growth hormone; HGF: hepatocyte growth factor; ICAM-1: intercellular adhesion molecule 1; IFN-γ: interferon gamma; IFNGR1: interferon gamma receptor 1; IL-10: interleukin 10; IL-12: interleukin 12; IL-13: interleukin 13; IL-17A: interleukin 17A; IL-17F: interleukin 17F; IL-18BP: interleukin 18 binding protein; IL-1α: interleukin 1 α; IL-1β: interleukin 1β; IL-1R1: interleukin 1 receptor type 1; IL-1R2: interleukin 1 receptor type 2; IL-1RA: interleukin 1 receptor antagonist; IL-2: interleukin 2; IL-22: interleukin 22; IL-23: interleukin 23; IL-27: interleukin 27; IL-28A: interleukin 28A; IL-2Rα: interleukin 2 receptor α; IL-31: interleukin 31; IL-33: interleukin 33; IL-5: interleukin 5; IL-6: interleukin 6; IL-6Rα: interleukin 6 receptor α; IL-8: interleukin 8; IL-9; interleukin 9; M-CSF: macrophage colony-stimulating factor; MDC: macrophage-derived chemokine; MIF: macrophage migration inhibitory factor; MMP-1: matrix metalloproteinase 1; MMP-12: matrix metalloproteinase 12; MMP-13: matrix metalloproteinase 13; MMP-2: matrix metalloproteinase 12; MMP-3: matrix metalloproteinase 3; MMP-7: matrix metalloproteinase 7; MMP-8: matrix metalloproteinase 8; MMP-9: matrix metalloproteinase 9; MPO: myeloperoxidase; N/A: not applicable; PF4: platelet factor 4; PTX3: pentraxin 3; SLE: systemic lupus erythematosus; TACI: transmembrane activator and calcium modulator and cyclophilin ligand interactor; TGF-β: transforming growth factor β; TNF-α: tumour necrosis factor α; TNFR1: tumour necrosis factor receptor 1; TNFR2: tumour necrosis factor receptor 2; VCAM-1: vascular cell adhesion protein 1; VEGF: vascular endothelial growth factor; VEGF-C: vascular endothelial growth factor C; VEGFR3: vascular endothelial growth factor receptor 3.

Supplementary Table 4. Serum levels of selected autoimmunity-associated cytokines in patients with active SLE versus healthy controls in the PRECISESADS study population

Cytokines	Active SLE		HC		*p* value
	n/N	Serum levels (pg/mL)	n/N	Serum levels (pg/mL)	
**BAFF**	230/348	6.05 (5.88–6.17)	295/546	5.97 (5.87–6.05)	**<0.001**
**CCL13**	148/348	2.15 (2.02–2.28)	178/546	2.15 (2.04–2.26)	0.652
**CCL17**	148/348	2.81 (2.62–3.02)	178/546	2.71 (2.50–2.87)	**<0.001**
**CCL18**	31/348	4.59 (4.38–4.64)	48/546	4.49 (4.38–4.55)	0.131
**CCL2**	31/348	2.67 (2.51–2.72)	48/546	2.61 (2.55–2.70)	0.463
**CCL20**	31/348	1.68 (1.48–1.88)	48/546	1.58 (1.46–1.78)	0.248
**CCL3**	25/348	2.27 (2.14–2.41)	42/546	2.30 (2.13–2.46)	0.464
**CCL4**	141/348	2.23 (2.08–2.44)	160/546	2.20 (1.95–2.52)	0.404
**CCL5**	31/348	4.72 (4.46–4.87)	48/546	4.69 (4.55–4.84)	0.693
**CCL7**	30/348	1.99 (1.82–2.23)	45/546	1.93 (1.62–2.21)	0.275
**CCL8**	148/348	1.93 (1.76–2.04)	178/546	1.82 (1.72–1.94)	**<0.001**
**CRP**	130/348	6.19 (5.75–6.59)	143/546	5.97 (5.42–6.36)	**0.001**
**CXCL1**	17/348	2.01 (1.83–2.20)	27/546	2.04 (1.91–2.30)	0.588
**CXCL10**	148/348	1.45 (1.28–1.77)	178/546	1.28 (1.13–1.40)	**<0.001**
**CXCL11**	31/348	2.46 (2.32–2.54)	48/546	2.36 (2.27–2.46)	**0.031**
**CXCL13**	148/348	2.03 (1.88–2.27)	175/546	1.78 (1.69–1.89)	**<0.001**
**CXCL16**	31/348	3.11 (3.05–3.17)	48/546	3.07 (3.02–3.10)	**0.025**
**CXCL5**	31/348	3.07 (2.90–3.28)	48/546	3.15 (3.04–3.38)	0.376
**CXCL9**	31/348	2.78 (2.68–2.96)	47/546	2.78 (2.67–2.86)	0.195
**DcR1**	31/348	3.45 (3.30–3.68)	48/546	3.53 (3.39–3.60)	0.753
**DcR3**	31/348	2.63 (2.48–2.73)	47/546	2.64 (2.48–2.75)	0.854
**E-selectin**	31/348	4.37 (4.26–4.46)	48/546	4.38 (4.21–4.50)	0.909
**EGF**	31/348	2.17 (1.93–2.41)	48/546	2.30 (2.04–2.39)	0.404
**Fas**	31/348	3.89 (3.81–4.01)	48/546	3.86 (3.82–3.90)	0.213
**FasL**	148/348	1.67 (1.47–1.79)	178/546	1.62 (1.54–1.73)	0.539
**FGF2**	31/348	2.16 (2.05–2.28)	48/546	2.12 (2.06–2.24)	0.511
**FGF21**	30/348	3.31 (3.01–3.40)	48/546	3.35 (3.17–3.46)	0.363
**G-CSF**	31/348	1.46 (1.36–1.54)	47/546	1.39 (1.31–1.46)	0.060
**Galactin-3**	31/348	3.91 (3.77–3.99)	48/546	3.85 (3.78–3.91)	0.119
**GDF15**	148/348	2.86 (2.70–3.11)	178/546	2.70 (2.57–2.84)	**<0.001**
**GH**	31/348	3.49 (3.15–3.70)	48/546	3.02 (2.61–3.42)	**0.009**
**HGF**	31/348	2.25 (2.14–2.40)	48/546	2.32 (2.23–2.40)	0.185
**ICAM-1**	31/348	5.39 (5.25–5.66)	48/546	5.40 (5.18–5.52)	0.421
**IFN-γ**	29/348	26.00 (21.99–32.86)	39/546	17.51 (13.79–20.56)	**<0.001**
**IFNGR1**	31/348	1.91 (1.83–2.00)	48/546	1.88 (1.79–1.92)	0.131
**IL-10**	21/348	0.54 (0.17–1.20)	15/546	0.28 (0.19–0.56)	0.304
**IL-12**	12/348	15.91 (6.58–28.55)	19/546	14.81 (6.11–30.33)	0.823
**IL-13**	15/348	2.20 (2.01–2.30)	24/546	2.33 (1.89–2.44)	0.265
**IL-17A**	16/348	1.94 (0.80–3.33)	15/546	2.99 (1.86–4.99)	0.166
**IL-17F**	15/348	1.30 (0.81–1.56)	12/546	0.94 (0.25–1.18)	0.152
**IL-18BP**	31/348	2.41 (2.32–2.60)	48/546	2.28 (2.05–2.44)	**0.009**
**IL-1α**	31/348	1.57 (1.47–1.85)	48/546	1.52 (1.38–1.78)	0.163
**IL-1β**	22/348	4.79 (2.78–7.64)	31/546	3.17 (1.89–4.81)	**0.017**
**IL-1R1**	31/348	3.17 (3.03–3.24)	48/546	3.11 (3.07–3.16)	0.269
**IL-1R2**	148/348	3.84 (3.75–3.91)	178/546	3.83 (3.77–3.89)	0.632
**IL-1RA**	146/348	3.00 (2.89–3.20)	178/546	2.92 (2.78–3.05)	**<0.001**
**IL-2**	19/348	1.98 (1.80–2.08)	28/546	1.88 (1.66–2.06)	0.313
**IL-22**	5/348	1.24 (0.69–1.24)	7/546	1.31 (0.84–1.46)	0.343
**IL-23**	31/348	2.56 (2.42–2.68)	48/546	2.56 (2.45–2.65)	0.740
**IL-27**	19/348	180.41 (82.90–283.65)	18/546	117.50 (48.72–188.24)	0.158
**IL-28A**	17/348	2.51 (2.12–2.75)	27/546	2.05 (1.80–2.16)	**0.006**
**IL-2Rα**	31/348	2.70 (2.60–2.87)	48/546	2.63 (2.53–2.72)	**0.014**

**IL-31**	25/348	2.09 (2.00–2.13)	43/546	2.06 (1.93–2.21)	0.884
**IL-33**	26/348	3.64 (2.34–5.13)	33/546	3.07 (2.25–5.80)	0.760
**IL-5**	29/348	3.70 (2.97–4.16)	38/546	3.90 (3.47–4.50)	0.156
**IL-6**	228/348	3.34 (2.91–3.64)	290/546	3.18 (2.91–3.38)	**<0.001**
**IL-6Rα**	31/348	4.07 (4.01–4.10)	48/546	4.07 (4.03–4.12)	0.791
**IL-8**	31/348	1.26 (1.13–1.40)	48/546	1.29 (1.20–1.36)	0.928
**IL-9**	6/348	287.00 (156.47–405.85)	6/546	263.29 (52.12–558.71)	0.936
**M-CSF**	10/348	55.82 (22.96–65.87)	16/546	58.42 (29.66–166.84)	0.343
**MDC**	31/348	2.87 (2.70–2.93)	48/546	2.83 (2.70–2.91)	0.433
**MIF**	30/348	3.75 (3.51–3.88)	48/546	3.60 (3.44–3.83)	0.159
**MMP-1**	31/348	3.31 (3.12–3.47)	48/546	3.30 (3.12–3.45)	0.854
**MMP-12**	5/348	107.31 (98.79–130.23)	7/546	53.62 (35.45–91.67)	0.415
**MMP-13**	28/348	2.53 (2.39–2.73)	40/546	2.54 (2.35–2.60)	0.319
**MMP-2**	131/348	5.33 (5.25–5.44)	143/546	5.35 (5.27–5.44)	0.280
**MMP-3**	31/348	4.29 (4.10–4.48)	47/546	4.09 (3.98–4.19)	**0.001**
**MMP-7**	31/348	3.37 (3.13–3.50)	48/546	3.32 (3.18–3.41)	0.498
**MMP-8**	147/348	3.53 (3.30–3.78)	178/546	3.57 (3.35–3.80)	0.212
**MMP-9**	31/348	5.25 (5.01–5.43)	48/546	5.35 (5.24–5.47)	**0.020**
**MPO**	31/348	5.11 (4.80–5.22)	48/546	5.08 (4.95–5.32)	0.784
**P-selectin**	31/348	4.58 (4.53–4.67)	48/546	4.60 (4.55–4.66)	0.566
**PF4**	30/348	6.87 (6.80–6.98)	45/546	6.92 (6.87–7.05)	0.060
**PTX3**	31/348	2.93 (2.82–3.27)	48/546	3.04 (2.92–3.22)	0.345
**TACI**	29/348	1.21 (0.97–1.28)	45/546	1.04 (0.91–1.20)	0.115
**TGF-β**	230/348	8.03 (7.83–8.15)	295/546	8.07 (7.71–8.20)	0.060
**TNF-α**	230/348	2.95 (2.67–3.16)	294/546	2.86 (2.63–2.98)	**<0.001**
**TNFR1**	148/348	3.72 (3.62–3.81)	178/546	3.63 (3.57–3.71)	**<0.001**
**TNFR2**	31/348	3.41 (3.22–3.58)	48/546	3.32 (3.18–3.36)	**0.013**
**VCAM-1**	31/348	6.04 (5.95–6.08)	48/546	5.86 (5.79–5.94)	**<0.001**
**VEGF**	31/348	1.72 (1.63–1.99)	48/546	1.84 (1.72–2.10)	0.054
**VEGF-C**	28/348	2.95 (2.79–3.08)	47/546	3.14 (2.92–3.26)	**0.013**
**VEGFR3**	31/348	2.14 (1.81–2.35)	48/546	2.14 (1.86–2.39)	0.677

Data are presented as medians (interquartile range). In case of missing values, the total number of patients with available data is indicated. All *p* values are derived from non-parametrical Mann-Whitney *U* tests. Statistically significant *p* values are in bold. N/A indicates that the comparison could not be conducted owing to low numbers of observations (n<4).

BAFF: B cell activating factor belonging to the tumour necrosis factor ligand family; CCL13: C-C motif chemokine ligand 13; CCL17: C-C motif chemokine ligand 17; CCL18; C-C motif chemokine ligand 18; CCL2; C-C motif chemokine ligand 2; CCL20; C-C motif chemokine ligand 20; CCL3; C-C motif chemokine ligand 3; CCL4; C-C motif chemokine ligand 4; CCL5; C-C motif chemokine ligand 5; CCL7; C-C motif chemokine ligand 7; CCL8; C-C motif chemokine ligand 8; CRP: C-reactive protein: CXCL1: C-X-C motif ligand 1; CXCL10: C-X-C motif ligand 10; CXCL11: C-X-C motif ligand 11; CXCL13: C-X-C motif ligand 13: CXCL16: C-X-C motif ligand 16; CXCL5: C-X-C motif ligand 5; CXCL9: C-X-C motif ligand 9; DcR1: decoy receptor 1; DcR3: decoy receptor 3; EGF: epidermal growth factor; FasL; Fas ligand; FGF2: fibroblast growth factor 2; FGF21: fibroblast growth factor 21; G-CSF: granulocyte colony-stimulating factor; GDF15: growth differentiation factor 15; GH: growth hormone; HC: healthy controls; HGF: hepatocyte growth factor; ICAM-1: intercellular adhesion molecule 1; IFN-γ: interferon γ; IFNGR1: interferon γ receptor 1; IL-10: interleukin 10; IL-12: interleukin 12; IL-13: interleukin 13; IL-17A: interleukin 17A; IL-17F: interleukin 17F; IL-18BP: interleukin 18 binding protein; IL-1α: interleukin 1 α; IL-1β: interleukin 1β; IL-1R1: interleukin 1 receptor type 1; IL-1R2: interleukin 1 receptor type 2; IL-1RA: interleukin 1 receptor antagonist; IL-2: interleukin 2; IL-22: interleukin 22; IL-23: interleukin 23; IL-27: interleukin 27; IL-28A: interleukin 28A; IL-2Rα: interleukin 2 receptor α; IL-31: interleukin 31; IL-33: interleukin 33; IL-5: interleukin 5; IL-6: interleukin 6; IL-6Rα: interleukin 6 receptor α; IL-8: interleukin 8; IL-9; interleukin 9; M-CSF: macrophage colony-stimulating factor; MDC: macrophage-derived chemokine; MIF: macrophage migration inhibitory factor; MMP-1: matrix metalloproteinase 1; MMP-12: matrix metalloproteinase 12; MMP-13: matrix metalloproteinase 13; MMP-2: matrix metalloproteinase 12; MMP-3: matrix metalloproteinase 3; MMP-7: matrix metalloproteinase 7; MMP-8: matrix metalloproteinase 8; MMP-9: matrix metalloproteinase 9; MPO: myeloperoxidase; N/A: not applicable; PF4: platelet factor 4; PTX3: pentraxin 3; SLE: systemic lupus erythematosus; TACI: transmembrane activator and calcium modulator and cyclophilin ligand interactor; TGF-β: transforming growth factor β; TNF-α: tumour necrosis factor α; TNFR1: tumour necrosis factor receptor 1; TNFR2: tumour necrosis factor receptor 2; VCAM-1: vascular cell adhesion protein 1; VEGF: vascular endothelial growth factor; VEGF-C: vascular endothelial growth factor C; VEGFR3: vascular endothelial growth factor receptor 3.

Supplementary Table 5. Serum levels of selected autoimmunity-associated cytokines in patients with inactive SLE versus healthy controls in the PRECISESADS study population

Cytokines	Inactive SLE		HC		*p* value
	n/N	Serum levels (pg/mL)	n/N	Serum levels (pg/mL)	
**BAFF**	21/45	5.90 (5.81–6.10)	295/546	5.97 (5.87–6.05)	0.685
**CCL13**	20/45	2.17 (2.02–2.31)	178/546	2.15 (2.04–2.26)	0.903
**CCL17**	20/45	2.75 (2.59–2.87)	178/546	2.71 (2.50–2.87)	0.407
**CCL18**	4/45	4.71 (4.69–4.73)	48/546	4.49 (4.38–4.55)	**0.014**
**CCL2**	4/45	2.62 (2.55–2.74)	48/546	2.61 (2.55–2.70)	0.778
**CCL20**	4/45	1.64 (1.58–1.72)	48/546	1.58 (1.46–1.78)	0.492
**CCL3**	4/45	2.30 (2.22–2.37)	42/546	2.30 (2.13–2.46)	0.984
**CCL4**	19/45	2.17 (2.09–2.39)	160/546	2.20 (1.95–2.52)	0.873
**CCL5**	4/45	4.61 (4.56–4.68)	48/546	4.69 (4.55–4.84)	0.455
**CCL7**	4/45	1.81 (1.73–1.94)	45/546	1.93 (1.62–2.21)	0.728
**CCL8**	20/45	1.80 (1.70–1.97)	178/546	1.82 (1.72–1.94)	0.995
**CRP**	16/45	5.79 (5.31–6.18)	143/546	5.97 (5.42–6.36)	0.448
**CXCL1**	2/45	2.19 (2.18–2.19)	27/546	2.04 (1.91–2.30)	N/A
**CXCL10**	20/45	1.33 (1.19–1.51)	178/546	1.28 (1.13–1.40)	0.104
**CXCL11**	4/45	2.40 (2.34–2.43)	48/546	2.36 (2.27–2.46)	0.770
**CXCL13**	20/45	1.83 (1.75–1.99)	175/546	1.78 (1.69–1.89)	0.150
**CXCL16**	4/45	3.18 (3.14–3.21)	48/546	3.07 (3.02–3.10)	**0.042**
**CXCL5**	4/45	3.27 (3.10–3.42)	48/546	3.15 (3.04–3.38)	0.655
**CXCL9**	4/45	2.64 (2.59–2.76)	47/546	2.78 (2.67–2.86)	0.483
**DcR1**	4/45	3.42 (3.33–3.53)	48/546	3.53 (3.39–3.60)	0.496
**DcR3**	4/45	2.66 (2.48–2.80)	47/546	2.64 (2.48–2.75)	0.726
**E-selectin**	4/45	4.37 (4.24–4.48)	48/546	4.38 (4.21–4.50)	0.987
**EGF**	4/45	2.37 (2.30–2.45)	48/546	2.30 (2.04–2.39)	0.377
**Fas**	4/45	3.93 (3.88–3.99)	48/546	3.86 (3.82–3.90)	0.182
**FasL**	20/45	1.59 (1.51–1.64)	178/546	1.62 (1.54–1.73)	0.165
**FGF2**	4/45	2.21 (2.18–2.24)	48/546	2.12 (2.06–2.24)	0.243
**FGF21**	4/45	3.39 (3.31–3.49)	48/546	3.35 (3.17–3.46)	0.279
**G-CSF**	4/45	1.56 (1.50–1.62)	47/546	1.39 (1.31–1.46)	**0.014**
**Galactin-3**	4/45	3.85 (3.81–3.90)	48/546	3.85 (3.78–3.91)	0.679
**GDF15**	20/45	2.91 (2.64–3.16)	178/546	2.70 (2.57–2.84)	**0.004**
**GH**	4/45	3.29 (2.99–3.46)	48/546	3.02 (2.61–3.42)	0.607
**HGF**	4/45	2.31 (2.08–2.61)	48/546	2.32 (2.23–2.40)	0.882
**ICAM-1**	4/45	5.47 (5.32–5.63)	48/546	5.40 (5.18–5.52)	0.539
**IFN-γ**	3/45	30.96 (26.61–31.72)	39/546	17.51 (13.79–20.56)	N/A
**IFNGR1**	4/45	1.88 (1.87–1.96)	48/546	1.88 (1.79–1.92)	0.496
**IL-10**	2/45	0.74 (0.59–0.89)	15/546	0.28 (0.19–0.56)	N/A
**IL-12**	1/45	0.18 (0.18–0.18)	19/546	14.81 (6.11–30.33)	N/A
**IL-13**	3/45	2.12 (2.00–2.17)	24/546	2.33 (1.89–2.44)	N/A
**IL-17A**	2/45	2.04 (1.06–3.01)	15/546	2.99 (1.86–4.99)	N/A
**IL-17F**	2/45	0.92 (0.79–1.05)	12/546	0.94 (0.25–1.18)	N/A
**IL-18BP**	4/45	2.32 (2.08–2.56)	48/546	2.28 (2.05–2.44)	0.655
**IL-1α**	4/45	1.69 (1.62–1.72)	48/546	1.52 (1.38–1.78)	0.328
**IL-1β**	3/45	3.75 (3.49–3.88)	31/546	3.17 (1.89–4.81)	N/A
**IL-1R1**	4/45	3.17 (3.10–3.21)	48/546	3.11 (3.07–3.16)	0.435
**IL-1R2**	20/45	3.83 (3.76–3.90)	178/546	3.83 (3.77–3.89)	0.941
**IL-1RA**	20/45	2.97 (2.83–3.09)	178/546	2.92 (2.78–3.05)	0.600
**IL-2**	3/45	1.95 (1.83–2.02)	28/546	1.88 (1.66–2.06)	N/A
**IL-22**	1/45	1.28 (1.28–1.28)	7/546	1.31 (0.84–1.46)	N/A
**IL-23**	4/45	2.74 (2.69–2.75)	48/546	2.56 (2.45–2.65)	**0.048**
**IL-27**	2/45	243.83 (231.24–256.42)	18/546	117.50 (48.72–188.24)	N/A
**IL-28A**	4/45	2.13 (1.84–2.44)	27/546	2.05 (1.80–2.16)	0.723
**IL-2Rα**	4/45	2.73 (2.66–2.93)	48/546	2.63 (2.53–2.72)	0.219

**IL-31**	4/45	2.13 (2.01–2.24)	43/546	2.06 (1.93–2.21)	0.634
**IL-33**	3/45	3.48 (2.66–4.92)	33/546	3.07 (2.25–5.80)	N/A
**IL-5**	3/45	3.75 (3.72–3.96)	38/546	3.90 (3.47–4.50)	N/A
**IL-6**	21/45	3.20 (3.03–3.49)	290/546	3.18 (2.91–3.38)	0.568
**IL-6Rα**	4/45	4.06 (4.02–4.11)	48/546	4.07 (4.03–4.12)	0.855
**IL-8**	4/45	1.42 (1.40–1.52)	48/546	1.29 (1.20–1.36)	**0.009**
**IL-9**	1/45	736.41 (736.41–736.41)	6/546	263.29 (52.12–558.71)	N/A
**M-CSF**	3/45	55.62 (46.33–63.61)	16/546	58.42 (29.66–166.84)	N/A
**MDC**	4/45	2.87 (2.80–2.96)	48/546	2.83 (2.70–2.91)	0.396
**MIF**	4/45	3.70 (3.53–3.92)	48/546	3.60 (3.44–3.83)	0.539
**MMP-1**	4/45	3.23 (3.14–3.34)	48/546	3.30 (3.12–3.45)	0.753
**MMP-12**	0/45	N/A	7/546	53.62 (35.45–91.67)	N/A
**MMP-13**	4/45	2.53 (2.38–2.65)	40/546	2.54 (2.35–2.60)	0.854
**MMP-2**	17/45	5.36 (5.32–5.41)	143/546	5.35 (5.27–5.44)	0.482
**MMP-3**	4/45	3.95 (3.92–4.09)	47/546	4.09 (3.98–4.19)	0.386
**MMP-7**	4/45	3.63 (3.57–3.68)	48/546	3.32 (3.18–3.41)	**0.008**
**MMP-8**	20/45	3.47 (3.37–3.63)	178/546	3.57 (3.35–3.80)	0.280
**MMP-9**	4/45	5.22 (5.04–5.43)	48/546	5.35 (5.24–5.47)	0.396
**MPO**	4/45	5.13 (4.87–5.33)	48/546	5.08 (4.95–5.32)	0.882
**P-selectin**	4/45	4.70 (4.57–4.81)	48/546	4.60 (4.55–4.66)	0.377
**PF4**	4/45	6.93 (6.88–6.98)	45/546	6.92 (6.87–7.05)	0.738
**PTX3**	4/45	3.07 (2.81–3.35)	48/546	3.04 (2.92–3.22)	0.959
**TACI**	4/45	1.18 (1.01–1.39)	45/546	1.04 (0.91–1.20)	0.315
**TGF-β**	21/45	7.96 (7.78–8.05)	295/546	8.07 (7.71–8.20)	0.043
**TNF-α**	21/45	2.96 (2.57–3.05)	294/546	2.86 (2.63–2.98)	0.669
**TNFR1**	20/45	3.68 (3.62–3.81)	178/546	3.63 (3.57–3.71)	**0.034**
**TNFR2**	4/45	3.39 (3.30–3.53)	48/546	3.32 (3.18–3.36)	0.292
**VCAM-1**	4/45	5.92 (5.80–6.12)	48/546	5.86 (5.79–5.94)	0.518
**VEGF**	4/45	1.88 (1.72–2.03)	48/546	1.84 (1.72–2.10)	0.855
**VEGF-C**	4/45	3.17 (3.11–3.20)	47/546	3.14 (2.92–3.26)	0.723
**VEGFR3**	4/45	2.38 (2.18–2.47)	48/546	2.14 (1.86–2.39)	0.420

Data are presented as medians (interquartile range). In case of missing values, the total number of patients with available data is indicated. All *p* values are derived from non-parametrical Mann-Whitney *U* tests. Statistically significant *p* values are in bold. N/A indicates that the comparison could not be conducted owing to low numbers of observations (n<4).

BAFF: B cell activating factor belonging to the tumour necrosis factor ligand family; CCL13: C-C motif chemokine ligand 13; CCL17: C-C motif chemokine ligand 17; CCL18; C-C motif chemokine ligand 18; CCL2; C-C motif chemokine ligand 2; CCL20; C-C motif chemokine ligand 20; CCL3; C-C motif chemokine ligand 3; CCL4; C-C motif chemokine ligand 4; CCL5; C-C motif chemokine ligand 5; CCL7; C-C motif chemokine ligand 7; CCL8; C-C motif chemokine ligand 8; CRP: C-reactive protein: CXCL1: C-X-C motif ligand 1; CXCL10: C-X-C motif ligand 10; CXCL11: C-X-C motif ligand 11; CXCL13: C-X-C motif ligand 13: CXCL16: C-X-C motif ligand 16; CXCL5: C-X-C motif ligand 5; CXCL9: C-X-C motif ligand 9; DcR1: decoy receptor 1; DcR3: decoy receptor 3; EGF: epidermal growth factor; FasL; Fas ligand; FGF2: fibroblast growth factor 2; FGF21: fibroblast growth factor 21; G-CSF: granulocyte colony-stimulating factor; GDF15: growth differentiation factor 15; GH: growth hormone; HC: healthy controls; HGF: hepatocyte growth factor; ICAM-1: intercellular adhesion molecule 1; IFN-γ: interferon γ; IFNGR1: interferon γ receptor 1; IL-10: interleukin 10; IL-12: interleukin 12; IL-13: interleukin 13; IL-17A: interleukin 17A; IL-17F: interleukin 17F; IL-18BP: interleukin 18 binding protein; IL-1α: interleukin 1 α; IL-1β: interleukin 1β; IL-1R1: interleukin 1 receptor type 1; IL-1R2: interleukin 1 receptor type 2; IL-1RA: interleukin 1 receptor antagonist; IL-2: interleukin 2; IL-22: interleukin 22; IL-23: interleukin 23; IL-27: interleukin 27; IL-28A: interleukin 28A; IL-2Rα: interleukin 2 receptor α; IL-31: interleukin 31; IL-33: interleukin 33; IL-5: interleukin 5; IL-6: interleukin 6; IL-6Rα: interleukin 6 receptor α; IL-8: interleukin 8; IL-9; interleukin 9; M-CSF: macrophage colony-stimulating factor; MDC: macrophage-derived chemokine; MIF: macrophage migration inhibitory factor; MMP-1: matrix metalloproteinase 1; MMP-12: matrix metalloproteinase 12; MMP-13: matrix metalloproteinase 13; MMP-2: matrix metalloproteinase 12; MMP-3: matrix metalloproteinase 3; MMP-7: matrix metalloproteinase 7; MMP-8: matrix metalloproteinase 8; MMP-9: matrix metalloproteinase 9; MPO: myeloperoxidase; N/A: not applicable; PF4: platelet factor 4; PTX3: pentraxin 3; SLE: systemic lupus erythematosus; TACI: transmembrane activator and calcium modulator and cyclophilin ligand interactor; TGF-β: transforming growth factor β; TNF-α: tumour necrosis factor α; TNFR1: tumour necrosis factor receptor 1; TNFR2: tumour necrosis factor receptor 2; VCAM-1: vascular cell adhesion protein 1; VEGF: vascular endothelial growth factor; VEGF-C: vascular endothelial growth factor C; VEGFR3: vascular endothelial growth factor receptor 3.

Supplementary Table 6. Serum levels of selected autoimmunity-associated cytokines in patients with autoimmune diseases* versus healthy controls in the PRECISESADS study population

Cytokines	AID		HC		*p* value
	n/N	Serum levels (pg/mL)	n/N	Serum levels (pg/mL)	
**BAFF**	735/1223	6.02 (5.89–6.13)	295/546	5.97 (5.87–6.05)	**<0.001**
**CCL13**	707/1223	2.19 (2.07–2.34)	178/546	2.15 (2.04–2.26)	**0.013**
**CCL17**	705/1223	2.80 (2.58–2.99)	178/546	2.71 (2.50–2.87)	**<0.001**
**CCL18**	158/1223	4.66 (4.51–4.75)	48/546	4.49 (4.38–4.55)	**<0.001**
**CCL2**	158/1223	2.66 (2.56–2.79)	48/546	2.61 (2.55–2.70)	**0.027**
**CCL20**	158/1223	1.71 (1.51–1.91)	48/546	1.58 (1.46–1.78)	**0.024**
**CCL3**	139/1223	2.32 (2.20–2.49)	42/546	2.30 (2.13–2.46)	0.626
**CCL4**	640/1223	2.17 (2.02–2.54)	160/546	2.20 (1.95–2.52)	0.717
**CCL5**	158/1223	4.66 (4.54–4.78)	48/546	4.69 (4.55–4.84)	0.511
**CCL7**	157/1223	2.07 (1.85–2.31)	45/546	1.93 (1.62–2.21)	**0.046**
**CCL8**	707/1223	1.88 (1.73–2.01)	178/546	1.82 (1.72–1.94)	**0.004**
**CRP**	602/1223	6.22 (5.75–6.58)	143/546	5.97 (5.42–6.36)	**<0.001**
**CXCL1**	91/1223	2.17 (2.00–2.32)	27/546	2.04 (1.91–2.30)	0.176
**CXCL10**	707/1223	1.45 (1.25–1.68)	178/546	1.28 (1.13–1.40)	**<0.001**
**CXCL11**	158/1223	2.45 (2.36–2.54)	48/546	2.36 (2.27–2.46)	**<0.001**
**CXCL13**	705/1223	1.95 (1.78–2.10)	175/546	1.78 (1.69–1.89)	**<0.001**
**CXCL16**	158/1223	3.05 (2.99–3.13)	48/546	3.07 (3.02–3.10)	0.606
**CXCL5**	158/1223	3.17 (3.02–3.33)	48/546	3.15 (3.04–3.38)	0.917
**CXCL9**	149/1223	2.85 (2.75–2.96)	47/546	2.78 (2.67–2.86)	**0.001**
**DcR1**	158/1223	3.51 (3.37–3.67)	48/546	3.53 (3.39–3.60)	0.990
**DcR3**	157/1223	2.72 (2.58–2.91)	47/546	2.64 (2.48–2.75)	**0.001**
**E-selectin**	158/1223	4.40 (4.27–4.52)	48/546	4.38 (4.21–4.50)	0.182
**EGF**	158/1223	2.23 (2.04–2.36)	48/546	2.30 (2.04–2.39)	0.339
**Fas**	158/1223	3.89 (3.77–3.97)	48/546	3.86 (3.82–3.90)	0.218
**FasL**	704/1223	1.60 (1.45–1.73)	178/546	1.62 (1.54–1.73)	**0.006**
**FGF2**	158/1223	2.15 (2.07–2.28)	48/546	2.12 (2.06–2.24)	0.255
**FGF21**	157/1223	3.41 (3.30–3.53)	48/546	3.35 (3.17–3.46)	**0.003**
**G-CSF**	155/1223	1.48 (1.36–1.58)	47/546	1.39 (1.31–1.46)	**0.001**
**Galactin-3**	158/1223	3.90 (3.83–3.98)	48/546	3.85 (3.78–3.91)	**0.005**
**GDF15**	705/1223	3.00 (2.80–3.19)	178/546	2.70 (2.57–2.84)	**<0.001**
**GH**	157/1223	3.10 (2.72–3.52)	48/546	3.02 (2.61–3.42)	0.516
**HGF**	158/1223	2.35 (2.24–2.48)	48/546	2.32 (2.23–2.40)	0.116
**ICAM-1**	158/1223	5.34 (5.18–5.60)	48/546	5.40 (5.18–5.52)	0.926
**IFN-γ**	111/1223	21.99 (15.85–30.58)	39/546	17.51 (13.79–20.56)	**0.002**
**IFNGR1**	158/1223	1.87 (1.80–1.97)	48/546	1.88 (1.79–1.92)	0.445
**IL-10**	65/1223	0.48 (0.20–0.84)	15/546	0.28 (0.19–0.56)	0.333
**IL-12**	53/1223	35.22 (18.86–70.29)	19/546	14.81 (6.11–30.33)	**0.001**
**IL-13**	72/1223	2.30 (2.12–2.44)	24/546	2.33 (1.89–2.44)	0.741
**IL-17A**	57/1223	2.28 (0.67–4.96)	15/546	2.99 (1.86–4.99)	0.375
**IL-17F**	50/1223	1.02 (0.52–1.35)	12/546	0.94 (0.25–1.18)	0.418
**IL-18BP**	158/1223	2.49 (2.24–2.62)	48/546	2.28 (2.05–2.44)	**<0.001**
**IL-1α**	158/1223	1.56 (1.43–1.81)	48/546	1.52 (1.38–1.78)	0.204
**IL-1β**	84/1223	3.21 (1.85–4.88)	31/546	3.17 (1.89–4.81)	0.564
**IL-1R1**	158/1223	3.12 (3.03–3.18)	48/546	3.11 (3.07–3.16)	0.687
**IL-1R2**	707/1223	3.81 (3.73–3.89)	178/546	3.83 (3.77–3.89)	**0.008**
**IL-1RA**	707/1223	2.96 (2.82–3.12)	178/546	2.92 (2.78–3.05)	**0.011**
**IL-2**	115/1223	1.99 (1.82–2.13)	28/546	1.88 (1.66–2.06)	0.083
**IL-22**	30/1223	1.46 (0.99–1.72)	7/546	1.31 (0.84–1.46)	0.506
**IL-23**	158/1223	2.58 (2.45–2.70)	48/546	2.56 (2.45–2.65)	0.259
**IL-27**	32/1223	161.14 (62.70–211.88)	18/546	117.50 (48.72–188.24)	0.467
**IL-28A**	91/1223	2.12 (1.80–2.56)	27/546	2.05 (1.80–2.16)	0.177

**IL-2Rα**	158/1223	2.73 (2.60–2.86)	48/546	2.63 (2.53–2.72)	**<0.001**
**IL-31**	145/1223	2.06 (1.95–2.18)	43/546	2.06 (1.93–2.21)	0.905
**IL-33**	109/1223	6.13 (2.89–12.73)	33/546	3.07 (2.25–5.80)	**0.004**
**IL-5**	111/1223	3.78 (2.88–4.61)	38/546	3.90 (3.47–4.50)	0.275
**IL-6**	730/1223	3.42 (3.06–3.72)	290/546	3.18 (2.91–3.38)	**<0.001**
**IL-6Rα**	158/1223	4.08 (4.03–4.13)	48/546	4.07 (4.03–4.12)	0.444
**IL-8**	158/1223	1.34 (1.22–1.48)	48/546	1.29 (1.20–1.36)	**0.007**
**IL-9**	33/1223	470.60 (241.68–1432.94)	6/546	263.29 (52.12–558.71)	0.302
**M-CSF**	60/1223	104.13 (43.40–172.14)	16/546	58.42 (29.66–166.84)	0.268
**MDC**	158/1223	2.86 (2.74–2.95)	48/546	2.83 (2.70–2.91)	0.190
**MIF**	157/1223	3.75 (3.58–3.95)	48/546	3.60 (3.44–3.83)	**<0.001**
**MMP-1**	158/1223	3.41 (3.25–3.59)	48/546	3.30 (3.12–3.45)	**0.009**
**MMP-12**	20/1223	103.16 (49.27–157.49)	7/546	53.62 (35.45–91.67)	0.280
**MMP-13**	128/1223	2.51 (2.33–2.65)	40/546	2.54 (2.35–2.60)	0.874
**MMP-2**	605/1223	5.35 (5.27–5.44)	143/546	5.35 (5.27–5.44)	0.792
**MMP-3**	158/1223	4.14 (3.95–4.37)	47/546	4.09 (3.98–4.19)	0.167
**MMP-7**	157/1223	3.46 (3.32–3.65)	48/546	3.32 (3.18–3.41)	**<0.001**
**MMP-8**	705/1223	3.49 (3.25–3.76)	178/546	3.57 (3.35–3.80)	**0.012**
**MMP-9**	158/1223	5.31 (5.12–5.53)	48/546	5.35 (5.24–5.47)	0.262
**MPO**	158/1223	5.11 (4.87–5.31)	48/546	5.08 (4.95–5.32)	0.710
**P-selectin**	158/1223	4.56 (4.46–4.65)	48/546	4.60 (4.55–4.66)	0.053
**PF4**	156/1223	6.95 (6.85–7.05)	45/546	6.92 (6.87–7.05)	0.888
**PTX3**	158/1223	3.01 (2.83–3.24)	48/546	3.04 (2.92–3.22)	0.669
**TACI**	149/1223	1.04 (0.85–1.21)	45/546	1.04 (0.91–1.20)	0.941
**TGF-β**	735/1223	8.00 (7.52–8.17)	295/546	8.07 (7.71–8.20)	**0.002**
**TNF-α**	734/1223	2.96 (2.65–3.14)	294/546	2.86 (2.63–2.98)	**<0.001**
**TNFR1**	707/1223	3.69 (3.60–3.79)	178/546	3.63 (3.57–3.71)	**<0.001**
**TNFR2**	157/1223	3.35 (3.21–3.55)	48/546	3.32 (3.18–3.36)	**0.009**
**VCAM-1**	158/1223	5.99 (5.86–6.12)	48/546	5.86 (5.79–5.94)	**<0.001**
**VEGF**	158/1223	1.94 (1.71–2.07)	48/546	1.84 (1.72–2.10)	0.811
**VEGF-C**	151/1223	2.94 (2.73–3.13)	47/546	3.14 (2.92–3.26)	**0.001**
**VEGFR3**	158/1223	2.00 (1.63–2.31)	48/546	2.14 (1.86–2.39)	**0.018**

Data are presented as medians (interquartile range). In case of missing values, the total number of patients with available data is indicated. All *p* values are derived from non-parametrical Mann-Whitney *U* tests. Statistically significant *p* values are in bold.

BAFF: B cell activating factor belonging to the tumour necrosis factor ligand family; CCL13: C-C motif chemokine ligand 13; CCL17: C-C motif chemokine ligand 17; CCL18; C-C motif chemokine ligand 18; CCL2; C-C motif chemokine ligand 2; CCL20; C-C motif chemokine ligand 20; CCL3; C-C motif chemokine ligand 3; CCL4; C-C motif chemokine ligand 4; CCL5; C-C motif chemokine ligand 5; CCL7; C-C motif chemokine ligand 7; CCL8; C-C motif chemokine ligand 8; CRP: C-reactive protein: CXCL1: C-X-C motif ligand 1; CXCL10: C-X-C motif ligand 10; CXCL11: C-X-C motif ligand 11; CXCL13: C-X-C motif ligand 13: CXCL16: C-X-C motif ligand 16; CXCL5: C-X-C motif ligand 5; CXCL9: C-X-C motif ligand 9; DcR1: decoy receptor 1; DcR3: decoy receptor 3; EGF: epidermal growth factor; FasL; Fas ligand; FGF2: fibroblast growth factor 2; FGF21: fibroblast growth factor 21; G-CSF: granulocyte colony-stimulating factor; GDF15: growth differentiation factor 15; GH: growth hormone; HC: healthy controls; HGF: hepatocyte growth factor; ICAM-1: intercellular adhesion molecule 1; IFN-γ: interferon γ; IFNGR1: interferon γ receptor 1; IL-10: interleukin 10; IL-12: interleukin 12; IL-13: interleukin 13; IL-17A: interleukin 17A; IL-17F: interleukin 17F; IL-18BP: interleukin 18 binding protein; IL-1α: interleukin 1 α; IL-1β: interleukin 1β; IL-1R1: interleukin 1 receptor type 1; IL-1R2: interleukin 1 receptor type 2; IL-1RA: interleukin 1 receptor antagonist; IL-2: interleukin 2; IL-22: interleukin 22; IL-23: interleukin 23; IL-27: interleukin 27; IL-28A: interleukin 28A; IL-2Rα: interleukin 2 receptor α; IL-31: interleukin 31; IL-33: interleukin 33; IL-5: interleukin 5; IL-6: interleukin 6; IL-6Rα: interleukin 6 receptor α; IL-8: interleukin 8; IL-9; interleukin 9; M-CSF: macrophage colony-stimulating factor; MDC: macrophage-derived chemokine; MIF: macrophage migration inhibitory factor; MMP-1: matrix metalloproteinase 1; MMP-12: matrix metalloproteinase 12; MMP-13: matrix metalloproteinase 13; MMP-2: matrix metalloproteinase 12; MMP-3: matrix metalloproteinase 3; MMP-7: matrix metalloproteinase 7; MMP-8: matrix metalloproteinase 8; MMP-9: matrix metalloproteinase 9; MPO: myeloperoxidase; PF4: platelet factor 4; PTX3: pentraxin 3; TACI: transmembrane activator and calcium modulator and cyclophilin ligand interactor; TGF-β: transforming growth factor β; TNF-α: tumour necrosis factor α; TNFR1: tumour necrosis factor receptor 1; TNFR2: tumour necrosis factor receptor 2; VCAM-1: vascular cell adhesion protein 1; VEGF: vascular endothelial growth factor; VEGF-C: vascular endothelial growth factor C; VEGFR3: vascular endothelial growth factor receptor 3.

* Mixed connective tissue disease, primary antiphospholipid syndrome, primary Sjögren’s syndrome, rheumatoid arthritis, and systemic sclerosis.

Supplementary Table 7. Serum levels of selected autoimmunity-associated cytokines in patients with SLE versus other autoimmune diseases* in the PRECISESADS study population

Cytokines	SLE		AID		*p* value
	**n/N**	**Serum levels (pg/mL)**	**n/N**	**Serum levels (pg/mL)**	
**BAFF**	258/422	6.04 (5.84–6.17)	735/1223	6.02 (5.89–6.13)	0.176
**CCL13**	194/422	2.16 (2.01–2.27)	707/1223	2.19 (2.07–2.34)	**0.001**
**CCL17**	194/422	2.80 (2.62–3.02)	705/1223	2.80 (2.58–2.99)	0.562
**CCL18**	45/422	4.60 (4.49–4.68)	158/1223	4.66 (4.51–4.75)	**0.046**
**CCL2**	45/422	2.65 (2.50–2.71)	158/1223	2.66 (2.56–2.79)	0.088
**CCL20**	45/422	1.68 (1.48–1.86)	158/1223	1.71 (1.51–1.91)	0.404
**CCL3**	38/422	2.31 (2.15–2.47)	139/1223	2.32 (2.20–2.49)	0.500
**CCL4**	184/422	2.23 (2.08–2.49)	640/1223	2.17 (2.02–2.54)	0.179
**CCL5**	45/422	4.67 (4.46–4.81)	158/1223	4.66 (4.54–4.78)	0.833
**CCL7**	44/422	1.99 (1.81–2.30)	157/1223	2.07 (1.85–2.31)	0.573
**CCL8**	194/422	1.92 (1.76–2.05)	707/1223	1.88 (1.73–2.01)	**0.007**
**CRP**	162/422	6.16 (5.61–6.57)	602/1223	6.22 (5.75–6.58)	0.468
**CXCL1**	25/422	2.09 (1.94–2.20)	91/1223	2.17 (2.00–2.32)	0.155
**CXCL10**	194/422	1.45 (1.29–1.77)	707/1223	1.45 (1.25–1.68)	0.125
**CXCL11**	45/422	2.46 (2.29–2.55)	158/1223	2.45 (2.36–2.54)	0.826
**CXCL13**	194/422	2.02 (1.85–2.24)	705/1223	1.95 (1.78–2.10)	**<0.001**
**CXCL16**	45/422	3.11 (3.04–3.19)	158/1223	3.05 (2.99–3.13)	**0.001**
**CXCL5**	45/422	3.06 (2.87–3.27)	158/1223	3.17 (3.02–3.33)	**0.040**
**CXCL9**	45/422	2.78 (2.67–3.00)	149/1223	2.85 (2.75–2.96)	0.197
**DcR1**	45/422	3.47 (3.33–3.64)	158/1223	3.51 (3.37–3.67)	0.610
**DcR3**	45/422	2.64 (2.49–2.73)	157/1223	2.72 (2.58–2.91)	**0.018**
**E-selectin**	45/422	4.40 (4.26–4.49)	158/1223	4.40 (4.27–4.52)	0.386
**EGF**	45/422	2.27 (2.06–2.44)	158/1223	2.23 (2.04–2.36)	0.462
**Fas**	45/422	3.89 (3.83–4.02)	158/1223	3.89 (3.77–3.97)	0.162
**FasL**	194/422	1.65 (1.49–1.78)	704/1223	1.60 (1.45–1.73)	**0.004**
**FGF2**	45/422	2.18 (2.06–2.26)	158/1223	2.15 (2.07–2.28)	0.889
**FGF21**	44/422	3.33 (3.18–3.45)	157/1223	3.41 (3.30–3.53)	**0.004**
**G-CSF**	45/422	1.48 (1.41–1.55)	155/1223	1.48 (1.36–1.58)	0.917
**Galactin-3**	45/422	3.89 (3.79–3.98)	158/1223	3.90 (3.83–3.98)	0.714
**GDF15**	194/422	2.89 (2.70–3.11)	705/1223	3.00 (2.80–3.19)	**<0.001**
**GH**	45/422	3.43 (2.96–3.62)	157/1223	3.10 (2.72–3.52)	**0.037**
**HGF**	45/422	2.26 (2.14–2.41)	158/1223	2.35 (2.24–2.48)	**0.015**
**ICAM-1**	45/422	5.39 (5.24–5.61)	158/1223	5.34 (5.18–5.60)	0.376
**IFN-γ**	32/422	26.09 (21.99–32.58)	111/1223	21.99 (15.85–30.58)	0.051
**IFNGR1**	45/422	1.88 (1.84–2.00)	158/1223	1.87 (1.80–1.97)	0.161
**IL-10**	23/422	0.54 (0.22–1.12)	65/1223	0.48 (0.20–0.84)	0.638
**IL-12**	13/422	10.70 (6.58–28.37)	53/1223	35.22 (18.86–70.29)	**0.005**
**IL-13**	22/422	2.21 (2.05–2.30)	72/1223	2.30 (2.12–2.44)	**0.038**
**IL-17A**	18/422	1.94 (0.62–3.35)	57/1223	2.28 (0.67–4.96)	0.539
**IL-17F**	18/422	1.11 (0.76–1.52)	50/1223	1.02 (0.52–1.35)	0.483
**IL-18BP**	45/422	2.43 (2.32–2.64)	158/1223	2.49 (2.24–2.62)	0.733
**IL-1α**	45/422	1.61 (1.44–1.72)	158/1223	1.56 (1.43–1.81)	0.921
**IL-1β**	25/422	4.49 (3.21–7.15)	84/1223	3.21 (1.85–4.88)	**0.012**
**IL-1R1**	45/422	3.17 (3.06–3.23)	158/1223	3.12 (3.03–3.18)	**0.049**
**IL-1R2**	194/422	3.84 (3.74–3.90)	707/1223	3.81 (3.73–3.89)	**0.017**
**IL-1RA**	192/422	3.01 (2.89–3.19)	707/1223	2.96 (2.82–3.12)	**0.001**
**IL-2**	32/422	1.98 (1.82–2.09)	115/1223	1.99 (1.82–2.13)	0.987
**IL-22**	7/422	1.24 (0.96–1.30)	30/1223	1.46 (0.99–1.72)	0.276
**IL-23**	45/422	2.61 (2.50–2.71)	158/1223	2.58 (2.45–2.70)	0.723
**IL-27**	21/422	185.83 (99.39–281.72)	32/1223	161.14 (62.70–211.88)	0.291
**IL-28A**	26/422	2.38 (1.86–2.68)	91/1223	2.12 (1.80–2.56)	0.343
**IL-2Rα**	45/422	2.72 (2.62–2.89)	158/1223	2.73 (2.60–2.86)	0.688

**IL-31**	38/422	2.10 (2.02–2.17)	145/1223	2.06 (1.95–2.18)	0.470
**IL-33**	29/422	3.48 (2.19–5.21)	109/1223	6.13 (2.89–12.73)	**0.013**
**IL-5**	32/422	3.72 (3.10–4.16)	111/1223	3.78 (2.88–4.61)	0.720
**IL-6**	256/422	3.33 (2.90–3.60)	730/1223	3.42 (3.06–3.72)	**0.006**
**IL-6Rα**	45/422	4.08 (4.01–4.11)	158/1223	4.08 (4.03–4.13)	0.457
**IL-8**	45/422	1.28 (1.16–1.41)	158/1223	1.34 (1.22–1.48)	0.068
**IL-9**	7/422	302.42 (194.84–529.25)	33/1223	470.60 (241.68–1432.94)	0.255
**M-CSF**	13/422	55.62 (31.77–67.73)	60/1223	104.13 (43.40–172.14)	**0.011**
**MDC**	45/422	2.87 (2.70–2.96)	158/1223	2.86 (2.74–2.95)	0.983
**MIF**	44/422	3.79 (3.55–3.95)	157/1223	3.75 (3.58–3.95)	0.755
**MMP-1**	45/422	3.29 (3.02–3.49)	158/1223	3.41 (3.25–3.59)	**0.030**
**MMP-12**	5/422	107.31 (98.79–130.23)	20/1223	103.16 (49.27–157.49)	0.760
**MMP-13**	41/422	2.58 (2.39–2.73)	128/1223	2.51 (2.33–2.65)	0.227
**MMP-2**	164/422	5.33 (5.26–5.42)	605/1223	5.35 (5.27–5.44)	0.150
**MMP-3**	45/422	4.22 (3.99–4.45)	158/1223	4.14 (3.95–4.37)	0.092
**MMP-7**	45/422	3.40 (3.24–3.55)	157/1223	3.46 (3.32–3.65)	**0.045**
**MMP-8**	193/422	3.51 (3.26–3.78)	705/1223	3.49 (3.25–3.76)	0.470
**MMP-9**	45/422	5.28 (5.02–5.44)	158/1223	5.31 (5.12–5.53)	0.182
**MPO**	45/422	5.12 (4.81–5.29)	158/1223	5.11 (4.87–5.31)	0.951
**P-selectin**	45/422	4.60 (4.54–4.68)	158/1223	4.56 (4.46–4.65)	0.121
**PF4**	44/422	6.87 (6.79–6.97)	156/1223	6.95 (6.85–7.05)	**0.004**
**PTX3**	45/422	2.97 (2.80–3.33)	158/1223	3.01 (2.83–3.24)	0.922
**TACI**	43/422	1.20 (0.92–1.28)	149/1223	1.04 (0.85–1.21)	0.106
**TGF-β**	258/422	8.01 (7.78–8.15)	735/1223	8.00 (7.52–8.17)	0.660
**TNF-α**	258/422	2.95 (2.63–3.15)	734/1223	2.96 (2.65–3.14)	0.863
**TNFR1**	194/422	3.72 (3.62–3.81)	707/1223	3.69 (3.60–3.79)	**0.029**
**TNFR2**	45/422	3.40 (3.19–3.58)	157/1223	3.35 (3.21–3.55)	0.500
**VCAM-1**	45/422	6.05 (5.95–6.16)	158/1223	5.99 (5.86–6.12)	0.110
**VEGF**	45/422	1.77 (1.65–2.00)	158/1223	1.94 (1.71–2.07)	**0.025**
**VEGF-C**	41/422	3.00 (2.80–3.16)	151/1223	2.94 (2.73–3.13)	0.372
**VEGFR3**	45/422	2.14 (1.75–2.36)	158/1223	2.00 (1.63–2.31)	0.169

Data are presented as medians (interquartile range). In case of missing values, the total number of patients with available data is indicated. All *p* values are derived from non-parametrical Mann-Whitney *U* tests. Statistically significant *p* values are in bold.

BAFF: B cell activating factor belonging to the tumour necrosis factor ligand family; CCL13: C-C motif chemokine ligand 13; CCL17: C-C motif chemokine ligand 17; CCL18; C-C motif chemokine ligand 18; CCL2; C-C motif chemokine ligand 2; CCL20; C-C motif chemokine ligand 20; CCL3; C-C motif chemokine ligand 3; CCL4; C-C motif chemokine ligand 4; CCL5; C-C motif chemokine ligand 5; CCL7; C-C motif chemokine ligand 7; CCL8; C-C motif chemokine ligand 8; CRP: C-reactive protein: CXCL1: C-X-C motif ligand 1; CXCL10: C-X-C motif ligand 10; CXCL11: C-X-C motif ligand 11; CXCL13: C-X-C motif ligand 13: CXCL16: C-X-C motif ligand 16; CXCL5: C-X-C motif ligand 5; CXCL9: C-X-C motif ligand 9; DcR1: decoy receptor 1; DcR3: decoy receptor 3; EGF: epidermal growth factor; FasL; Fas ligand; FGF2: fibroblast growth factor 2; FGF21: fibroblast growth factor 21; G-CSF: granulocyte colony-stimulating factor; GDF15: growth differentiation factor 15; GH: growth hormone; HGF: hepatocyte growth factor; ICAM-1: intercellular adhesion molecule 1; IFN-γ: interferon γ; IFNGR1: interferon γ receptor 1; IL-10: interleukin 10; IL-12: interleukin 12; IL-13: interleukin 13; IL-17A: interleukin 17A; IL-17F: interleukin 17F; IL-18BP: interleukin 18 binding protein; IL-1α: interleukin 1 α; IL-1β: interleukin 1β; IL-1R1: interleukin 1 receptor type 1; IL-1R2: interleukin 1 receptor type 2; IL-1RA: interleukin 1 receptor antagonist; IL-2: interleukin 2; IL-22: interleukin 22; IL-23: interleukin 23; IL-27: interleukin 27; IL-28A: interleukin 28A; IL-2Rα: interleukin 2 receptor α; IL-31: interleukin 31; IL-33: interleukin 33; IL-5: interleukin 5; IL-6: interleukin 6; IL-6Rα: interleukin 6 receptor α; IL-8: interleukin 8; IL-9; interleukin 9; M-CSF: macrophage colony-stimulating factor; MDC: macrophage-derived chemokine; MIF: macrophage migration inhibitory factor; MMP-1: matrix metalloproteinase 1; MMP-12: matrix metalloproteinase 12; MMP-13: matrix metalloproteinase 13; MMP-2: matrix metalloproteinase 12; MMP-3: matrix metalloproteinase 3; MMP-7: matrix metalloproteinase 7; MMP-8: matrix metalloproteinase 8; MMP-9: matrix metalloproteinase 9; MPO: myeloperoxidase; PF4: platelet factor 4; N/A: not applicable; N/A: not applicable; PTX3: pentraxin 3; SLE: systemic lupus erythematosus; TACI: transmembrane activator and calcium modulator and cyclophilin ligand interactor; TGF-β: transforming growth factor β; TNF-α: tumour necrosis factor α; TNFR1: tumour necrosis factor receptor 1; TNFR2: tumour necrosis factor receptor 2; VCAM-1: vascular cell adhesion protein 1; VEGF: vascular endothelial growth factor; VEGF-C: vascular endothelial growth factor C; VEGFR3: vascular endothelial growth factor receptor 3.

* Mixed connective tissue disease, primary antiphospholipid syndrome, primary Sjögren's syndrome, rheumatoid arthritis, and systemic sclerosis.

9 **Supplementary Table 8.** Serum levels of selected autoimmunity-associated cytokines in patients with pAPS versus healthy controls in the PRECISESADS study population

Cytokines	pAPS		HC		*p* value
	n/N	Serum levels (pg/mL)	n/N	Serum levels (pg/mL)	
**BAFF**	34/97	5.96 (5.68–6.03)	295/546	5.97 (5.87–6.05)	0.466
**CCL13**	57/97	2.07 (1.93–2.21)	178/546	2.15 (2.04–2.26)	**0.012**
**CCL17**	57/97	2.57 (2.34–2.90)	178/546	2.71 (2.50–2.87)	0.080
**CCL18**	7/97	4.53 (4.45–4.60)	48/546	4.49 (4.38–4.55)	0.279
**CCL2**	7/97	2.52 (2.46–2.53)	48/546	2.61 (2.55–2.70)	**0.003**
**CCL20**	7/97	1.77 (1.62–2.11)	48/546	1.58 (1.46–1.78)	0.114
**CCL3**	7/97	2.30 (2.22–2.43)	42/546	2.30 (2.13–2.46)	0.989
**CCL4**	50/97	2.18 (2.06–2.32)	160/546	2.20 (1.95–2.52)	0.967
**CCL5**	7/97	4.66 (4.58–4.72)	48/546	4.69 (4.55–4.84)	0.465
**CCL7**	7/97	1.92 (1.72–1.95)	45/546	1.93 (1.62–2.21)	0.788
**CCL8**	57/97	1.74 (1.66–1.87)	178/546	1.82 (1.72–1.94)	**0.010**
**CRP**	52/97	6.22 (5.91–6.36)	143/546	5.97 (5.42–6.36)	**0.015**
**CXCL1**	5/97	2.30 (2.09–2.46)	27/546	2.04 (1.91–2.30)	0.082
**CXCL10**	57/97	1.31 (1.19–1.51)	178/546	1.28 (1.13–1.40)	0.050
**CXCL11**	7/97	2.39 (2.34–2.49)	48/546	2.36 (2.27–2.46)	0.331
**CXCL13**	57/97	1.82 (1.74–1.95)	175/546	1.78 (1.69–1.89)	0.073
**CXCL16**	7/97	3.01 (2.98–3.09)	48/546	3.07 (3.02–3.10)	0.392
**CXCL5**	7/97	3.09 (3.02–3.25)	48/546	3.15 (3.04–3.38)	0.795
**CXCL9**	6/97	2.72 (2.71–2.77)	47/546	2.78 (2.67–2.86)	0.407
**DcR1**	7/97	3.61 (3.52–3.77)	48/546	3.53 (3.39–3.60)	0.088
**DcR3**	7/97	2.71 (2.65–2.82)	47/546	2.64 (2.48–2.75)	0.110
**E-selectin**	7/97	4.37 (4.28–4.51)	48/546	4.38 (4.21–4.50)	0.739
**EGF**	7/97	2.31 (2.22–2.34)	48/546	2.30 (2.04–2.39)	0.757
**Fas**	7/97	3.86 (3.80–3.92)	48/546	3.86 (3.82–3.90)	0.912
**FasL**	57/97	1.70 (1.55–1.80)	178/546	1.62 (1.54–1.73)	**0.043**
**FGF2**	7/97	2.14 (2.10–2.17)	48/546	2.12 (2.06–2.24)	0.771
**FGF21**	7/97	3.35 (3.20–3.47)	48/546	3.35 (3.17–3.46)	0.791
**G-CSF**	7/97	1.44 (1.32–1.52)	47/546	1.39 (1.31–1.46)	0.652
**Galactin-3**	7/97	3.86 (3.83–3.89)	48/546	3.85 (3.78–3.91)	0.683
**GDF15**	57/97	2.74 (2.67–2.98)	178/546	2.70 (2.57–2.84)	**0.005**
**GH**	7/97	3.10 (2.93–3.31)	48/546	3.02 (2.61–3.42)	0.762
**HGF**	7/97	2.35 (2.33–2.49)	48/546	2.32 (2.23–2.40)	0.216
**ICAM-1**	7/97	5.34 (5.31–5.52)	48/546	5.40 (5.18–5.52)	0.853
**IFN-γ**	4/97	48.08 (30.91–223.32)	39/546	17.51 (13.79–20.56)	**0.008**
**IFNGR1**	7/97	1.97 (1.91–2.01)	48/546	1.88 (1.79–1.92)	**0.022**
**IL-10**	4/97	0.37 (0.19–0.81)	15/546	0.28 (0.19–0.56)	0.841
**IL-12**	3/97	15.89 (10.20–16.37)	19/546	14.81 (6.11–30.33)	N/A
**IL-13**	5/97	2.29 (2.29–2.29)	24/546	2.33 (1.89–2.44)	0.706
**IL-17A**	4/97	1.11 (0.46–13.72)	15/546	2.99 (1.86–4.99)	0.395
**IL-17F**	3/97	1.17 (0.82–1.56)	12/546	0.94 (0.25–1.18)	N/A
**IL-18BP**	7/97	2.45 (2.42–2.58)	48/546	2.28 (2.05–2.44)	**0.017**
**IL-1α**	7/97	1.54 (1.47–1.65)	48/546	1.52 (1.38–1.78)	0.810
**IL-1β**	3/97	3.90 (3.56–5.34)	31/546	3.17 (1.89–4.81)	N/A
**IL-1R1**	7/97	3.16 (3.10–3.22)	48/546	3.11 (3.07–3.16)	0.365
**IL-1R2**	57/97	3.85 (3.78–3.89)	178/546	3.83 (3.77–3.89)	0.513
**IL-1RA**	57/97	2.97 (2.89–3.13)	178/546	2.92 (2.78–3.05)	**0.012**
**IL-2**	6/97	1.92 (1.85–2.14)	28/546	1.88 (1.66–2.06)	0.259
**IL-22**	1/97	0.81 (0.81–0.81)	7/546	1.31 (0.84–1.46)	N/A
**IL-23**	7/97	2.57 (2.52–2.63)	48/546	2.56 (2.45–2.65)	0.791
**IL-27**	3/97	162.33 (108.01–959.04)	18/546	117.50 (48.72–188.24)	N/A
**IL-28A**	3/97	2.35 (2.07–2.64)	27/546	2.05 (1.80–2.16)	N/A
**IL-2Rα**	7/97	2.72 (2.66–2.88)	48/546	2.63 (2.53–2.72)	**0.007**

**IL-31**	6/97	2.13 (1.80–2.19)	43/546	2.06 (1.93–2.21)	0.939
**IL-33**	4/97	22.42 (4.52–70.84)	33/546	3.07 (2.25–5.80)	0.117
**IL-5**	4/97	4.25 (3.61–4.53)	38/546	3.90 (3.47–4.50)	0.881
**IL-6**	33/97	3.24 (2.70–3.42)	290/546	3.18 (2.91–3.38)	0.588
**IL-6Rα**	7/97	4.09 (4.08–4.13)	48/546	4.07 (4.03–4.12)	0.216
**IL-8**	7/97	1.48 (1.37–1.62)	48/546	1.29 (1.20–1.36)	**0.007**
**IL-9**	2/97	2319.28 (1424.38–3214.17)	6/546	263.29 (52.12–558.71)	N/A
**M-CSF**	3/97	354.61 (193.93–578.10)	16/546	58.42 (29.66–166.84)	N/A
**MDC**	7/97	2.83 (2.79–2.90)	48/546	2.83 (2.70–2.91)	0.776
**MIF**	7/97	3.69 (3.51–3.91)	48/546	3.60 (3.44–3.83)	0.378
**MMP-1**	7/97	3.32 (3.16–3.43)	48/546	3.30 (3.12–3.45)	0.931
**MMP-12**	2/97	76.15 (56.19–96.11)	7/546	53.62 (35.45–91.67)	N/A
**MMP-13**	6/97	2.61 (2.32–2.69)	40/546	2.54 (2.35–2.60)	0.557
**MMP-2**	52/97	5.28 (5.23–5.33)	143/546	5.35 (5.27–5.44)	**<0.001**
**MMP-3**	7/97	4.10 (3.84–4.31)	47/546	4.09 (3.98–4.19)	0.980
**MMP-7**	7/97	3.38 (3.34–3.57)	48/546	3.32 (3.18–3.41)	0.181
**MMP-8**	57/97	3.57 (3.37–3.84)	178/546	3.57 (3.35–3.80)	0.770
**MMP-9**	7/97	5.35 (5.24–5.49)	48/546	5.35 (5.24–5.47)	0.931
**MPO**	7/97	5.44 (5.26–5.50)	48/546	5.08 (4.95–5.32)	0.059
**P-selectin**	7/97	4.63 (4.60–4.66)	48/546	4.60 (4.55–4.66)	0.544
**PF4**	7/97	6.86 (6.82–7.03)	45/546	6.92 (6.87–7.05)	0.461
**PTX3**	7/97	3.48 (3.04–3.52)	48/546	3.04 (2.92–3.22)	0.067
**TACI**	7/97	1.08 (0.86–1.21)	45/546	1.04 (0.91–1.20)	0.862
**TGF-β**	34/97	8.13 (7.55–8.20)	295/546	8.07 (7.71–8.20)	0.899
**TNF-α**	34/97	2.87 (1.80–3.08)	294/546	2.86 (2.63–2.98)	0.490
**TNFR1**	57/97	3.67 (3.63–3.78)	178/546	3.63 (3.57–3.71)	**0.019**
**TNFR2**	6/97	3.35 (3.24–3.42)	48/546	3.32 (3.18–3.36)	0.426
**VCAM-1**	7/97	5.96 (5.84–6.08)	48/546	5.86 (5.79–5.94)	0.116
**VEGF**	7/97	1.76 (1.74–1.91)	48/546	1.84 (1.72–2.10)	0.378
**VEGF-C**	7/97	2.72 (2.60–3.10)	47/546	3.14 (2.92–3.26)	0.067
**VEGFR3**	7/97	2.48 (1.84–2.50)	48/546	2.14 (1.86–2.39)	0.587

Data are presented as medians (interquartile range). In case of missing values, the total number of patients with available data is indicated. All *p* values are derived from non-parametrical Mann-Whitney *U* tests. Statistically significant *p* values are in bold.

BAFF: B cell activating factor belonging to the tumour necrosis factor ligand family; CCL13: C-C motif chemokine ligand 13; CCL17: C-C motif chemokine ligand 17; CCL18; C-C motif chemokine ligand 18; CCL2; C-C motif chemokine ligand 2; CCL20; C-C motif chemokine ligand 20; CCL3; C-C motif chemokine ligand 3; CCL4; C-C motif chemokine ligand 4; CCL5; C-C motif chemokine ligand 5; CCL7; C-C motif chemokine ligand 7; CCL8; C-C motif chemokine ligand 8; CRP: C-reactive protein: CXCL1: C-X-C motif ligand 1; CXCL10: C-X-C motif ligand 10; CXCL11: C-X-C motif ligand 11; CXCL13: C-X-C motif ligand 13: CXCL16: C-X-C motif ligand 16; CXCL5: C-X-C motif ligand 5; CXCL9: C-X-C motif ligand 9; DcR1: decoy receptor 1; DcR3: decoy receptor 3; EGF: epidermal growth factor; FasL; Fas ligand; FGF2: fibroblast growth factor 2; FGF21: fibroblast growth factor 21; G-CSF: granulocyte colony-stimulating factor; GDF15: growth differentiation factor 15; GH: growth hormone; HC: healthy controls; HGF: hepatocyte growth factor; ICAM-1: intercellular adhesion molecule 1; IFN-γ: interferon γ; IFNGR1: interferon γ receptor 1; IL-10: interleukin 10; IL-12: interleukin 12; IL-13: interleukin 13; IL-17A: interleukin 17A; IL-17F: interleukin 17F; IL-18BP: interleukin 18 binding protein; IL-1α: interleukin 1 α; IL-1β: interleukin 1β; IL-1R1: interleukin 1 receptor type 1; IL-1R2: interleukin 1 receptor type 2; IL-1RA: interleukin 1 receptor antagonist; IL-2: interleukin 2; IL-22: interleukin 22; IL-23: interleukin 23; IL-27: interleukin 27; IL-28A: interleukin 28A; IL-2Rα: interleukin 2 receptor α; IL-31: interleukin 31; IL-33: interleukin 33; IL-5: interleukin 5; IL-6: interleukin 6; IL-6Rα: interleukin 6 receptor α; IL-8: interleukin 8; IL-9; interleukin 9; M-CSF: macrophage colony-stimulating factor; MDC: macrophage-derived chemokine; MIF: macrophage migration inhibitory factor; MMP-1: matrix metalloproteinase 1; MMP-12: matrix metalloproteinase 12; MMP-13: matrix metalloproteinase 13; MMP-2: matrix metalloproteinase 12; MMP-3: matrix metalloproteinase 3; MMP-7: matrix metalloproteinase 7; MMP-8: matrix metalloproteinase 8; MMP-9: matrix metalloproteinase 9; MPO: myeloperoxidase; N/A: not applicable; pAPS: primary antiphospholipid syndrome; PF4: platelet factor 4; PTX3: pentraxin 3; TACI: transmembrane activator and calcium modulator and cyclophilin ligand interactor; TGF-β: transforming growth factor β; TNF-α: tumour necrosis factor α; TNFR1: tumour necrosis factor receptor 1; TNFR2: tumour necrosis factor receptor 2; VCAM-1: vascular cell adhesion protein 1; VEGF: vascular endothelial growth factor; VEGF-C: vascular endothelial growth factor C; VEGFR3: vascular endothelial growth factor receptor 3.

Supplementary Table 9. Serum levels of selected autoimmunity-associated cytokines in patients with SLE versus pAPS in the PRECISESADS study population

Cytokines	SLE		pAPS		*p* value
	n/N	Serum levels (pg/mL)	n/N	Serum levels (pg/mL)	
**BAFF**	258/422	6.04 (5.84–6.17)	34/97	5.96 (5.68–6.03)	**0.006**
**CCL13**	194/422	2.16 (2.01–2.27)	57/97	2.07 (1.93–2.21)	**0.040**
**CCL17**	194/422	2.80 (2.62–3.02)	57/97	2.57 (2.34–2.90)	**<0.001**
**CCL18**	45/422	4.60 (4.49–4.68)	7/97	4.53 (4.45–4.60)	0.545
**CCL2**	45/422	2.65 (2.50–2.71)	7/97	2.52 (2.46–2.53)	**0.028**
**CCL20**	45/422	1.68 (1.48–1.86)	7/97	1.77 (1.62–2.11)	0.228
**CCL3**	38/422	2.31 (2.15–2.47)	7/97	2.30 (2.22–2.43)	0.863
**CCL4**	184/422	2.23 (2.08–2.49)	50/97	2.18 (2.06–2.32)	0.314
**CCL5**	45/422	4.67 (4.46–4.81)	7/97	4.66 (4.58–4.72)	0.694
**CCL7**	44/422	1.99 (1.81–2.30)	7/97	1.92 (1.72–1.95)	0.298
**CCL8**	194/422	1.92 (1.76–2.05)	57/97	1.74 (1.66–1.87)	**<0.001**
**CRP**	162/422	6.16 (5.61–6.57)	52/97	6.22 (5.91–6.36)	0.774
**CXCL1**	25/422	2.09 (1.94–2.20)	5/97	2.30 (2.09–2.46)	0.106
**CXCL10**	194/422	1.45 (1.29–1.77)	57/97	1.31 (1.19–1.51)	**0.001**
**CXCL11**	45/422	2.46 (2.29–2.55)	7/97	2.39 (2.34–2.49)	0.668
**CXCL13**	194/422	2.02 (1.85–2.24)	57/97	1.82 (1.74–1.95)	**<0.001**
**CXCL16**	45/422	3.11 (3.04–3.19)	7/97	3.01 (2.98–3.09)	0.097
**CXCL5**	45/422	3.06 (2.87–3.27)	7/97	3.09 (3.02–3.25)	0.563
**CXCL9**	45/422	2.78 (2.67–3.00)	6/97	2.72 (2.71–2.77)	0.236
**DcR1**	45/422	3.47 (3.33–3.64)	7/97	3.61 (3.52–3.77)	0.097
**DcR3**	45/422	2.64 (2.49–2.73)	7/97	2.71 (2.65–2.82)	0.249
**E-selectin**	45/422	4.40 (4.26–4.49)	7/97	4.37 (4.28–4.51)	0.793
**EGF**	45/422	2.27 (2.06–2.44)	7/97	2.31 (2.22–2.34)	0.599
**Fas**	45/422	3.89 (3.83–4.02)	7/97	3.86 (3.80–3.92)	0.461
**FasL**	194/422	1.65 (1.49–1.78)	57/97	1.70 (1.55–1.80)	0.156
**FGF2**	45/422	2.18 (2.06–2.26)	7/97	2.14 (2.10–2.17)	0.658
**FGF21**	44/422	3.33 (3.18–3.45)	7/97	3.35 (3.20–3.47)	0.841
**G-CSF**	45/422	1.48 (1.41–1.55)	7/97	1.44 (1.32–1.52)	0.362
**Galactin-3**	45/422	3.89 (3.79–3.98)	7/97	3.86 (3.83–3.89)	0.618
**GDF15**	194/422	2.89 (2.70–3.11)	57/97	2.74 (2.67–2.98)	**0.036**
**GH**	45/422	3.43 (2.96–3.62)	7/97	3.10 (2.93–3.31)	0.356
**HGF**	45/422	2.26 (2.14–2.41)	7/97	2.35 (2.33–2.49)	0.143
**ICAM-1**	45/422	5.39 (5.24–5.61)	7/97	5.34 (5.31–5.52)	0.937
**IFN-γ**	32/422	26.09 (21.99–32.58)	4/97	48.08 (30.91–223.32)	0.144
**IFNGR1**	45/422	1.88 (1.84–2.00)	7/97	1.97 (1.91–2.01)	0.316
**IL-10**	23/422	0.54 (0.22–1.12)	4/97	0.37 (0.19–0.81)	0.785
**IL-12**	13/422	10.70 (6.58–28.37)	3/97	15.89 (10.20–16.37)	N/A
**IL-13**	22/422	2.21 (2.05–2.30)	5/97	2.29 (2.29–2.29)	0.471
**IL-17A**	18/422	1.94 (0.62–3.35)	4/97	1.11 (0.46–13.72)	0.798
**IL-17F**	18/422	1.11 (0.76–1.52)	3/97	1.17 (0.82–1.56)	N/A
**IL-18BP**	45/422	2.43 (2.32–2.64)	7/97	2.45 (2.42–2.58)	0.461
**IL-1α**	45/422	1.61 (1.44–1.72)	7/97	1.54 (1.47–1.65)	0.655
**IL-1β**	25/422	4.49 (3.21–7.15)	3/97	3.90 (3.56–5.34)	N/A
**IL-1R1**	45/422	3.17 (3.06–3.23)	7/97	3.16 (3.10–3.22)	0.854
**IL-1R2**	194/422	3.84 (3.74–3.90)	57/97	3.85 (3.78–3.89)	0.631
**IL-1RA**	192/422	3.01 (2.89–3.19)	57/97	2.97 (2.89–3.13)	0.388
**IL-2**	32/422	1.98 (1.82–2.09)	6/97	1.92 (1.85–2.14)	0.779
**IL-22**	7/422	1.24 (0.96–1.30)	1/97	0.81 (0.81–0.81)	N/A
**IL-23**	45/422	2.61 (2.50–2.71)	7/97	2.57 (2.52–2.63)	0.629
**IL-27**	21/422	185.83 (99.39–281.72)	3/97	162.33 (108.01–959.04)	N/A
**IL-28A**	26/422	2.38 (1.86–2.68)	3/97	2.35 (2.07–2.64)	N/A
**IL-2Rα**	45/422	2.72 (2.62–2.89)	7/97	2.72 (2.66–2.88)	0.581

**IL-31**	38/422	2.10 (2.02–2.17)	6/97	2.13 (1.80–2.19)	0.771
**IL-33**	29/422	3.48 (2.19–5.21)	4/97	22.42 (4.52–70.84)	0.116
**IL-5**	32/422	3.72 (3.10–4.16)	4/97	4.25 (3.61–4.53)	0.465
**IL-6**	256/422	3.33 (2.90–3.60)	33/97	3.24 (2.70–3.42)	0.142
**IL-6Rα**	45/422	4.08 (4.01–4.11)	7/97	4.09 (4.08–4.13)	0.329
**IL-8**	45/422	1.28 (1.16–1.41)	7/97	1.48 (1.37–1.62)	**0.021**
**IL-9**	7/422	302.42 (194.84–529.25)	2/97	2319.28 (1424.38–3214.17)	N/A
**M-CSF**	13/422	55.62 (31.77–67.73)	3/97	354.61 (193.93–578.10)	N/A
**MDC**	45/422	2.87 (2.70–2.96)	7/97	2.83 (2.79–2.90)	0.753
**MIF**	44/422	3.79 (3.55–3.95)	7/97	3.69 (3.51–3.91)	0.904
**MMP-1**	45/422	3.29 (3.02–3.49)	7/97	3.32 (3.16–3.43)	0.854
**MMP-12**	5/422	107.31 (98.79–130.23)	2/97	76.15 (56.19–96.11)	N/A
**MMP-13**	41/422	2.58 (2.39–2.73)	6/97	2.61 (2.32–2.69)	0.848
**MMP-2**	164/422	5.33 (5.26–5.42)	52/97	5.28 (5.23–5.33)	**0.001**
**MMP-3**	45/422	4.22 (3.99–4.45)	7/97	4.10 (3.84–4.31)	0.213
**MMP-7**	45/422	3.40 (3.24–3.55)	7/97	3.38 (3.34–3.57)	0.655
**MMP-8**	193/422	3.51 (3.26–3.78)	57/97	3.57 (3.37–3.84)	0.184
**MMP-9**	45/422	5.28 (5.02–5.44)	7/97	5.35 (5.24–5.49)	0.290
**MPO**	45/422	5.12 (4.81–5.29)	7/97	5.44 (5.26–5.50)	**0.049**
**P-selectin**	45/422	4.60 (4.54–4.68)	7/97	4.63 (4.60–4.66)	0.581
**PF4**	44/422	6.87 (6.79–6.97)	7/97	6.86 (6.82–7.03)	0.564
**PTX3**	45/422	2.97 (2.80–3.33)	7/97	3.48 (3.04–3.52)	0.068
**TACI**	43/422	1.20 (0.92–1.28)	7/97	1.08 (0.86–1.21)	0.485
**TGF-β**	258/422	8.01 (7.78–8.15)	34/97	8.13 (7.55–8.20)	0.324
**TNF-α**	258/422	2.95 (2.63–3.15)	34/97	2.87 (1.80–3.08)	0.211
**TNFR1**	194/422	3.72 (3.62–3.81)	57/97	3.67 (3.63–3.78)	0.135
**TNFR2**	45/422	3.40 (3.19–3.58)	6/97	3.35 (3.24–3.42)	0.658
**VCAM-1**	45/422	6.05 (5.95–6.16)	7/97	5.96 (5.84–6.08)	0.356
**VEGF**	45/422	1.77 (1.65–2.00)	7/97	1.76 (1.74–1.91)	1.000
**VEGF-C**	41/422	3.00 (2.80–3.16)	7/97	2.72 (2.60–3.10)	0.293
**VEGFR3**	45/422	2.14 (1.75–2.36)	7/97	2.48 (1.84–2.50)	0.272

Data are presented as medians (interquartile range). In case of missing values, the total number of patients with available data is indicated. All *p* values are derived from non-parametrical Mann-Whitney *U* tests. Statistically significant *p* values are in bold.

BAFF: B cell activating factor belonging to the tumour necrosis factor ligand family; CCL13: C-C motif chemokine ligand 13; CCL17: C-C motif chemokine ligand 17; CCL18; C-C motif chemokine ligand 18; CCL2; C-C motif chemokine ligand 2; CCL20; C-C motif chemokine ligand 20; CCL3; C-C motif chemokine ligand 3; CCL4; C-C motif chemokine ligand 4; CCL5; C-C motif chemokine ligand 5; CCL7; C-C motif chemokine ligand 7; CCL8; C-C motif chemokine ligand 8; CRP: C-reactive protein: CXCL1: C-X-C motif ligand 1; CXCL10: C-X-C motif ligand 10; CXCL11: C-X-C motif ligand 11; CXCL13: C-X-C motif ligand 13: CXCL16: C-X-C motif ligand 16; CXCL5: C-X-C motif ligand 5; CXCL9: C-X-C motif ligand 9; DcR1: decoy receptor 1; DcR3: decoy receptor 3; EGF: epidermal growth factor; FasL; Fas ligand; FGF2: fibroblast growth factor 2; FGF21: fibroblast growth factor 21; G-CSF: granulocyte colony-stimulating factor; GDF15: growth differentiation factor 15; GH: growth hormone; HC: healthy controls; HGF: hepatocyte growth factor; ICAM-1: intercellular adhesion molecule 1; IFN-γ: interferon γ; IFNGR1: interferon γ receptor 1; IL-10: interleukin 10; IL-12: interleukin 12; IL-13: interleukin 13; IL-17A: interleukin 17A; IL-17F: interleukin 17F; IL-18BP: interleukin 18 binding protein; IL-1α: interleukin 1 α; IL-1β: interleukin 1β; IL-1R1: interleukin 1 receptor type 1; IL-1R2: interleukin 1 receptor type 2; IL-1RA: interleukin 1 receptor antagonist; IL-2: interleukin 2; IL-22: interleukin 22; IL-23: interleukin 23; IL-27: interleukin 27; IL-28A: interleukin 28A; IL-2Rα: interleukin 2 receptor α; IL-31: interleukin 31; IL-33: interleukin 33; IL-5: interleukin 5; IL-6: interleukin 6; IL-6Rα: interleukin 6 receptor α; IL-8: interleukin 8; IL-9; interleukin 9; M-CSF: macrophage colony-stimulating factor; MDC: macrophage-derived chemokine; MIF: macrophage migration inhibitory factor; MMP-1: matrix metalloproteinase 1; MMP-12: matrix metalloproteinase 12; MMP-13: matrix metalloproteinase 13; MMP-2: matrix metalloproteinase 12; MMP-3: matrix metalloproteinase 3; MMP-7: matrix metalloproteinase 7; MMP-8: matrix metalloproteinase 8; MMP-9: matrix metalloproteinase 9; MPO: myeloperoxidase; N/A: not applicable; pAPS: primary antiphospholipid syndrome; PF4: platelet factor 4; PTX3: pentraxin 3; SLE: systemic lupus erythematosus; TACI: transmembrane activator and calcium modulator and cyclophilin ligand interactor; TGF-β: transforming growth factor β; TNF-α: tumour necrosis factor α; TNFR1: tumour necrosis factor receptor 1; TNFR2: tumour necrosis factor receptor 2; VCAM-1: vascular cell adhesion protein 1; VEGF: vascular endothelial growth factor; VEGF-C: vascular endothelial growth factor C; VEGFR3: vascular endothelial growth factor receptor 3.

Supplementary Table 10. Serum levels of selected autoimmunity-associated cytokines in patients with pAPS versus other autoimmune diseases* in the PRECISESADS study population

Cytokines	pAPS		AID		*p* value
	n/N	Serum levels (pg/mL)	n/N	Serum levels (pg/mL)	
**BAFF**	34/97	5.96 (5.68–6.03)	701/1126	6.03 (5.89–6.13)	**0.009**
**CCL13**	57/97	2.07 (1.93–2.21)	650/1126	2.21 (2.08–2.34)	**<0.001**
**CCL17**	57/97	2.57 (2.34–2.90)	648/1126	2.81 (2.60–3.00)	**<0.001**
**CCL18**	7/97	4.53 (4.45–4.60)	151/1126	4.67 (4.51–4.75)	0.216
**CCL2**	7/97	2.52 (2.46–2.53)	151/1126	2.67 (2.58–2.80)	**0.001**
**CCL20**	7/97	1.77 (1.62–2.11)	151/1126	1.70 (1.51–1.90)	0.473
**CCL3**	7/97	2.30 (2.22–2.43)	132/1126	2.32 (2.20–2.50)	0.859
**CCL4**	50/97	2.18 (2.06–2.32)	590/1126	2.17 (2.02–2.55)	0.982
**CCL5**	7/97	4.66 (4.58–4.72)	151/1126	4.66 (4.54–4.78)	0.627
**CCL7**	7/97	1.92 (1.72–1.95)	150/1126	2.09 (1.85–2.31)	0.126
**CCL8**	57/97	1.74 (1.66–1.87)	650/1126	1.89 (1.74–2.01)	**<0.001**
**CRP**	52/97	6.22 (5.91–6.36)	550/1126	6.22 (5.75–6.59)	0.818
**CXCL1**	5/97	2.30 (2.09–2.46)	86/1126	2.17 (1.99–2.30)	0.216
**CXCL10**	57/97	1.31 (1.19–1.51)	650/1126	1.47 (1.26–1.69)	**0.002**
**CXCL11**	7/97	2.39 (2.34–2.49)	151/1126	2.45 (2.36–2.54)	0.415
**CXCL13**	57/97	1.82 (1.74–1.95)	648/1126	1.96 (1.80–2.12)	**<0.001**
**CXCL16**	7/97	3.01 (2.98–3.09)	151/1126	3.06 (2.99–3.13)	0.720
**CXCL5**	7/97	3.09 (3.02–3.25)	151/1126	3.17 (3.03–3.34)	0.670
**CXCL9**	6/97	2.72 (2.71–2.77)	143/1126	2.86 (2.76–2.97)	**0.020**
**DcR1**	7/97	3.61 (3.52–3.77)	151/1126	3.51 (3.35–3.66)	0.086
**DcR3**	7/97	2.71 (2.65–2.82)	150/1126	2.72 (2.58–2.92)	0.966
**E-selectin**	7/97	4.37 (4.28–4.51)	151/1126	4.40 (4.27–4.52)	0.829
**EGF**	7/97	2.31 (2.22–2.34)	151/1126	2.23 (2.04–2.35)	0.213
**Fas**	7/97	3.86 (3.80–3.92)	151/1126	3.89 (3.77–3.97)	0.720
**FasL**	57/97	1.70 (1.55–1.80)	647/1126	1.59 (1.45–1.72)	**<0.001**
**FGF2**	7/97	2.14 (2.10–2.17)	151/1126	2.16 (2.07–2.29)	0.710
**FGF21**	7/97	3.35 (3.20–3.47)	150/1126	3.41 (3.31–3.53)	0.262
**G-CSF**	7/97	1.44 (1.32–1.52)	148/1126	1.48 (1.37–1.58)	0.259
**Galactin-3**	7/97	3.86 (3.83–3.89)	151/1126	3.90 (3.83–3.98)	0.368
**GDF15**	57/97	2.74 (2.67–2.98)	648/1126	3.01 (2.83–3.20)	**<0.001**
**GH**	7/97	3.10 (2.93–3.31)	150/1126	3.10 (2.71–3.53)	0.993
**HGF**	7/97	2.35 (2.33–2.49)	151/1126	2.36 (2.23–2.48)	0.540
**ICAM-1**	7/97	5.34 (5.31–5.52)	151/1126	5.34 (5.18–5.61)	0.645
**IFN-γ**	4/97	48.08 (30.91–223.32)	107/1126	21.98 (15.79–29.00)	0.051
**IFNGR1**	7/97	1.97 (1.91–2.01)	151/1126	1.87 (1.80–1.96)	0.067
**IL-10**	4/97	0.37 (0.19–0.81)	61/1126	0.48 (0.20–0.84)	0.859
**IL-12**	3/97	15.89 (10.20–16.37)	50/1126	37.17 (23.16–77.15)	N/A
**IL-13**	5/97	2.29 (2.29–2.29)	67/1126	2.30 (2.12–2.44)	0.723
**IL-17A**	4/97	1.11 (0.46–13.72)	53/1126	2.34 (0.72–4.96)	0.650
**IL-17F**	3/97	1.17 (0.82–1.56)	47/1126	1.02 (0.52–1.34)	N/A
**IL-18BP**	7/97	2.45 (2.42–2.58)	151/1126	2.49 (2.23–2.62)	0.642
**IL-1α**	7/97	1.54 (1.47–1.65)	151/1126	1.57 (1.43–1.82)	0.771
**IL-1β**	3/97	3.90 (3.56–5.34)	81/1126	3.18 (1.74–4.81)	N/A
**IL-1R1**	7/97	3.16 (3.10–3.22)	151/1126	3.12 (3.03–3.18)	0.325
**IL-1R2**	57/97	3.85 (3.78–3.89)	650/1126	3.80 (3.72–3.88)	**0.018**
**IL-1RA**	57/97	2.97 (2.89–3.13)	650/1126	2.95 (2.81–3.12)	0.256
**IL-2**	6/97	1.92 (1.85–2.14)	109/1126	1.99 (1.79–2.13)	0.739
**IL-22**	1/97	0.81 (0.81–0.81)	29/1126	1.46 (1.01–1.73)	N/A
**IL-23**	7/97	2.57 (2.52–2.63)	151/1126	2.58 (2.45–2.70)	0.774
**IL-27**	3/97	162.33 (108.01–959.04)	29/1126	161.14 (65.70–211.40)	N/A
**IL-28A**	3/97	2.35 (2.07–2.64)	88/1126	2.12 (1.80–2.56)	N/A
**IL-2Rα**	7/97	2.72 (2.66–2.88)	151/1126	2.74 (2.59–2.86)	0.529

**IL-31**	6/97	2.13 (1.80–2.19)	139/1126	2.06 (1.95–2.18)	0.816
**IL-33**	4/97	22.42 (4.52–70.84)	105/1126	6.13 (2.89–12.04)	0.318
**IL-5**	4/97	4.25 (3.61–4.53)	107/1126	3.67 (2.88–4.61)	0.681
**IL-6**	33/97	3.24 (2.70–3.42)	697/1126	3.42 (3.07–3.73)	**0.004**
**IL-6Rα**	7/97	4.09 (4.08–4.13)	151/1126	4.08 (4.03–4.13)	0.373
**IL-8**	7/97	1.48 (1.37–1.62)	151/1126	1.33 (1.22–1.48)	0.094
**IL-9**	2/97	2319.28 (1424.38–3214.17)	31/1126	417.44 (241.68–1409.85)	N/A
**M-CSF**	3/97	354.61 (193.93–578.10)	57/1126	104.13 (43.52–162.59)	N/A
**MDC**	7/97	2.83 (2.79–2.90)	151/1126	2.86 (2.74–2.96)	0.701
**MIF**	7/97	3.69 (3.51–3.91)	150/1126	3.75 (3.59–3.95)	0.610
**MMP-1**	7/97	3.32 (3.16–3.43)	151/1126	3.41 (3.25–3.59)	0.170
**MMP-12**	2/97	76.15 (56.19–96.11)	18/1126	103.16 (57.08–160.43)	N/A
**MMP-13**	6/97	2.61 (2.32–2.69)	122/1126	2.51 (2.33–2.64)	0.668
**MMP-2**	52/97	5.28 (5.23–5.33)	553/1126	5.37 (5.28–5.45)	**<0.001**
**MMP-3**	7/97	4.10 (3.84–4.31)	151/1126	4.15 (3.95–4.37)	0.670
**MMP-7**	7/97	3.38 (3.34–3.57)	150/1126	3.46 (3.32–3.65)	0.734
**MMP-8**	57/97	3.57 (3.37–3.84)	648/1126	3.48 (3.23–3.75)	**0.048**
**MMP-9**	7/97	5.35 (5.24–5.49)	151/1126	5.30 (5.12–5.54)	0.563
**MPO**	7/97	5.44 (5.26–5.50)	151/1126	5.08 (4.86–5.27)	**0.025**
**P-selectin**	7/97	4.63 (4.60–4.66)	151/1126	4.56 (4.45–4.65)	0.281
**PF4**	7/97	6.86 (6.82–7.03)	149/1126	6.95 (6.85–7.05)	0.439
**PTX3**	7/97	3.48 (3.04–3.52)	151/1126	3.01 (2.82–3.23)	**0.035**
**TACI**	7/97	1.08 (0.86–1.21)	142/1126	1.04 (0.86–1.21)	0.914
**TGF-β**	34/97	8.13 (7.55–8.20)	701/1126	8.00 (7.52–8.16)	0.299
**TNF-α**	34/97	2.87 (1.80–3.08)	700/1126	2.96 (2.65–3.14)	0.169
**TNFR1**	57/97	3.67 (3.63–3.78)	650/1126	3.69 (3.60–3.80)	0.839
**TNFR2**	6/97	3.35 (3.24–3.42)	151/1126	3.36 (3.20–3.56)	0.913
**VCAM-1**	7/97	5.96 (5.84–6.08)	151/1126	5.99 (5.86–6.12)	0.764
**VEGF**	7/97	1.76 (1.74–1.91)	151/1126	1.95 (1.71–2.09)	0.206
**VEGF-C**	7/97	2.72 (2.60–3.10)	144/1126	2.94 (2.75–3.13)	0.496
**VEGFR3**	7/97	2.48 (1.84–2.50)	151/1126	1.99 (1.60–2.30)	0.122

Data are presented as medians (interquartile range). In case of missing values, the total number of patients with available data is indicated. All *p* values are derived from non-parametrical Mann-Whitney *U* tests. Statistically significant *p* values are in bold.

BAFF: B cell activating factor belonging to the tumour necrosis factor ligand family; CCL13: C-C motif chemokine ligand 13; CCL17: C-C motif chemokine ligand 17; CCL18; C-C motif chemokine ligand 18; CCL2; C-C motif chemokine ligand 2; CCL20; C-C motif chemokine ligand 20; CCL3; C-C motif chemokine ligand 3; CCL4; C-C motif chemokine ligand 4; CCL5; C-C motif chemokine ligand 5; CCL7; C-C motif chemokine ligand 7; CCL8; C-C motif chemokine ligand 8; CRP: C-reactive protein: CXCL1: C-X-C motif ligand 1; CXCL10: C-X-C motif ligand 10; CXCL11: C-X-C motif ligand 11; CXCL13: C-X-C motif ligand 13: CXCL16: C-X-C motif ligand 16; CXCL5: C-X-C motif ligand 5; CXCL9: C-X-C motif ligand 9; DcR1: decoy receptor 1; DcR3: decoy receptor 3; EGF: epidermal growth factor; FasL; Fas ligand; FGF2: fibroblast growth factor 2; FGF21: fibroblast growth factor 21; G-CSF: granulocyte colony-stimulating factor; GDF15: growth differentiation factor 15; GH: growth hormone; HGF: hepatocyte growth factor; ICAM-1: intercellular adhesion molecule 1; IFN-γ: interferon γ; IFNGR1: interferon γ receptor 1; IL-10: interleukin 10; IL-12: interleukin 12; IL-13: interleukin 13; IL-17A: interleukin 17A; IL-17F: interleukin 17F; IL-18BP: interleukin 18 binding protein; IL-1α: interleukin 1 α; IL-1β: interleukin 1β; IL-1R1: interleukin 1 receptor type 1; IL-1R2: interleukin 1 receptor type 2; IL-1RA: interleukin 1 receptor antagonist; IL-2: interleukin 2; IL-22: interleukin 22; IL-23: interleukin 23; IL-27: interleukin 27; IL-28A: interleukin 28A; IL-2Rα: interleukin 2 receptor α; IL-31: interleukin 31; IL-33: interleukin 33; IL-5: interleukin 5; IL-6: interleukin 6; IL-6Rα: interleukin 6 receptor α; IL-8: interleukin 8; IL-9; interleukin 9; M-CSF: macrophage colony-stimulating factor; MDC: macrophage-derived chemokine; MIF: macrophage migration inhibitory factor; MMP-1: matrix metalloproteinase 1; MMP-12: matrix metalloproteinase 12; MMP-13: matrix metalloproteinase 13; MMP-2: matrix metalloproteinase 12; MMP-3: matrix metalloproteinase 3; MMP-7: matrix metalloproteinase 7; MMP-8: matrix metalloproteinase 8; MMP-9: matrix metalloproteinase 9; MPO: myeloperoxidase; N/A: not applicable; pAPS: primary antiphospholipid syndrome; PF4: platelet factor 4; PTX3: pentraxin 3; TACI: transmembrane activator and calcium modulator and cyclophilin ligand interactor; TGF-β: transforming growth factor β; TNF-α: tumour necrosis factor α; TNFR1: tumour necrosis factor receptor 1; TNFR2: tumour necrosis factor receptor 2; VCAM-1: vascular cell adhesion protein 1; VEGF: vascular endothelial growth factor; VEGF-C: vascular endothelial growth factor C; VEGFR3: vascular endothelial growth factor receptor 3.

* Mixed connective tissue disease, primary Sjögren's syndrome, rheumatoid arthritis, and systemic sclerosis.

12 **Supplementary Table 11.** Correlations between cytokine levels and disease activity in patients with SLE

Cytokines	n/N	Coefficient	*p* value
**BAFF**	32/297	0.15	**0.019**
**CCL13**	23/297	0.01	0.848
**CCL17**	13/297	0.08	0.312
**CCL18**	18/297	-0.21	0.221
**CCL2**	25/297	0.15	0.401
**CCL20**	21/297	0.32	0.059
**CCL3**	29/297	-0.24	0.201
**CCL4**	32/297	-0.09	0.264
**CCL5**	13/297	0.15	0.402
**CCL7**	251/297	0.26	0.142
**CCL8**	168/297	0.21	**0.005**
**CRP**	146/297	0.11	0.176
**CXCL1**	35/297	-0.36	0.133
**CXCL10**	35/297	0.04	0.595
**CXCL11**	35/297	0.18	0.290
**CXCL13**	35/297	0.18	**0.021**
**CXCL16**	35/297	-0.05	0.769
**CXCL5**	35/297	-0.22	0.208
**CXCL9**	168/297	0.25	0.152
**DcR1**	34/297	0.20	0.248
**DcR3**	35/297	0.02	0.893
**E-selectin**	35/297	-0.03	0.883
**EGF**	35/297	-0.09	0.613
**Fas**	168/297	0.12	0.492
**FasL**	19/297	-0.01	0.849
**FGF2**	35/297	-0.02	0.907
**FGF21**	35/297	0.00	0.994
**G-CSF**	35/297	-0.11	0.535
**Galactin-3**	35/297	0.12	0.505
**GDF15**	35/297	0.12	0.137
**GH**	166/297	0.07	0.667
**HGF**	35/297	0.19	0.280
**ICAM-1**	168/297	0.02	0.908
**IFN-γ**	18/297	0.00	0.995
**IFNGR1**	35/297	0.22	0.202
**IL-10**	35/297	0.18	0.403
**IL-12**	22/297	0.44	0.133
**IL-13**	35/297	0.36	0.141
**IL-17A**	35/297	0.29	0.241
**IL-17F**	21/297	0.35	0.170
**IL-18BP**	29/297	0.21	0.230
**IL-1α**	17/297	-0.01	0.977
**IL-1β**	168/297	0.20	0.330
**IL-1R1**	249/297	0.38	**0.023**
**IL-1R2**	35/297	0.01	0.942
**IL-1RA**	35/297	0.19	**0.015**
**IL-23**	168/297	0.06	0.742
**IL-27**	34/297	0.08	0.721
**IL-28A**	168/297	0.27	0.230
**IL-2Rα**	35/297	0.11	0.516
**IL-31**	34/297	-0.13	0.503
**IL-33**	35/297	0.19	0.321
**IL-5**	29/297	0.09	0.631
**IL-6**	160/297	0.12	0.062

**IL-6Rα**	35/297	0.06	0.718
**IL-8**	35/297	-0.01	0.961
**M-CSF**	32/297	0.28	0.347
**MDC**	148/297	-0.11	0.518
**MIF**	35/297	0.19	0.293
**MMP-1**	35/297	0.25	0.146
**MMP-13**	167/297	0.11	0.536
**MMP-2**	35/297	0.01	0.879
**MMP-3**	35/297	0.33	0.053
**MMP-7**	35/297	0.04	0.800
**MMP-8**	35/297	0.16	**0.035**
**MMP-9**	35/297	0.16	0.352
**MPO**	34/297	0.32	0.061
**P-selectin**	35/297	-0.35	**0.040**
**PF4**	33/297	-0.06	0.750
**PTX3**	168/297	0.38	**0.023**
**TACI**	251/297	-0.17	0.337
**TGF-β**	251/297	0.07	0.246
**TNF-α**	168/297	0.12	0.061
**TNFR1**	35/297	0.10	0.188
**TNFR2**	35/297	0.00	0.981
**VCAM-1**	35/297	0.27	0.118
**VEGF**	35/297	-0.21	0.234
**VEGF-C**	32/297	-0.20	0.279
**VEGFR3**	35/297	-0.16	0.348

Spearman's rank correlation coefficients of correlations between levels of different cytokines and SLEDAI-2K scores. In case of missing values, the total number of patients with available data is indicated. Statistically significant *p* values are in bold. Only comparisons with sufficient numbers of observations ($n \geq 10$) are included.

BAFF: B cell activating factor belonging to the tumour necrosis factor ligand family; CCL13: C-C motif chemokine ligand 13; CCL17: C-C motif chemokine ligand 17; CCL18: C-C motif chemokine ligand 18; CCL2: C-C motif chemokine ligand 2; CCL20: C-C motif chemokine ligand 20; CCL3: C-C motif chemokine ligand 3; CCL4: C-C motif chemokine ligand 4; CCL5: C-C motif chemokine ligand 5; CCL7: C-C motif chemokine ligand 7; CCL8: C-C motif chemokine ligand 8; CRP: C-reactive protein: CXCL1: C-X-C motif ligand 1; CXCL10: C-X-C motif ligand 10; CXCL11: C-X-C motif ligand 11; CXCL13: C-X-C motif ligand 13: CXCL16: C-X-C motif ligand 16; CXCL5: C-X-C motif ligand 5; CXCL9: C-X-C motif ligand 9; DcR1: decoy receptor 1; DcR3: decoy receptor 3; EGF: epidermal growth factor; FasL; Fas ligand; FGF2: fibroblast growth factor 2; FGF21: fibroblast growth factor 21; G-CSF: granulocyte colony-stimulating factor; GDF15: growth differentiation factor 15; GH: growth hormone; HGF: hepatocyte growth factor; ICAM-1: intercellular adhesion molecule 1; IFN-γ: interferon γ; IFNGR1: interferon γ receptor 1; IL-12: interleukin 12; IL-13: interleukin 13; IL-17A: interleukin 17A; IL-17F: interleukin 17F; IL-18BP: interleukin 18 binding protein; IL-1α: interleukin 1 α; IL-1β: interleukin 1β; IL-1R1: interleukin 1 receptor type 1; IL-1R2: interleukin 1 receptor type 2; IL-1RA: interleukin 1 receptor antagonist; IL-23: interleukin 23; IL-27: interleukin 27; IL-28A: interleukin 28A; IL-2Rα: interleukin 2 receptor α; IL-31: interleukin 31; IL-33: interleukin 33; IL-5: interleukin 5; IL-6: interleukin 6; IL-6Rα: interleukin 6 receptor α; IL-8: interleukin 8; IL-9; interleukin 9; M-CSF: macrophage colony-stimulating factor; MDC: macrophage-derived chemokine; MIF: macrophage migration inhibitory factor; MMP-1: matrix metalloproteinase 1; MMP-12: matrix metalloproteinase 12; MMP-13: matrix metalloproteinase 13; MMP-2: matrix metalloproteinase 12; MMP-3: matrix metalloproteinase 3; MMP-7: matrix metalloproteinase 7; MMP-8: matrix metalloproteinase 8; MMP-9: matrix metalloproteinase 9; PF4: platelet factor 4; PTX3: pentraxin 3; SLEDAI-2K: Systemic Lupus Erythematosus Disease Activity Index 2000; TACI: transmembrane activator and calcium modulator and cyclophilin ligand interactor; TGF-β: transforming growth factor β; TNF-α: tumour necrosis factor α; TNFR1: tumour necrosis factor receptor 1; TNFR2: tumour necrosis factor receptor 2; VCAM-1: vascular cell adhesion protein 1; VEGF: vascular endothelial growth factor; VEGF-C: vascular endothelial growth factor C; VEGFR3: vascular endothelial growth factor receptor 3.

Supplementary Table 12. Correlations between positivity for selected serological markers and the cSLEDAI-2K CNS domain

Serological marker	n/N	Coefficient	*p* value
**B2G**	338/347	0.01	0.801
**B2M**	338/347	0.01	0.808
**C3C**	347/347	-0.06	0.285
**C4**	347/347	0.05	0.342
**CHROMATIN**	312/347	0.02	0.671
**CLG**	342/347	0.01	0.861
**CLM**	342/347	0.04	0.505
**DNA**	342/347	-0.07	0.198
**PFLC**	220/347	0.05	0.468
**RF**	346/347	0.07	0.202
**SM**	314/347	-0.06	0.266
**SSA52**	304/347	-0.02	0.760
**SSA60**	305/347	0.01	0.829
**SSB**	315/347	0.02	0.739
**U1RNP**	315/347	-0.04	0.518

Phi (φ) correlations between autoantibody positivity or low levels of C3c or C4 and the cSLEDAI-2K CNS domain. In case of missing values, the total number of patients with available data is indicated. Statistically significant *p* values are in bold. B2G: anti-β_2 glycoprotein I IgG; B2M: anti-β_2 glycoprotein I IgM; C3c: complement component 3c; C4: complement component 4; CLG: anti-cardiolipin IgG; CLM: anti-cardiolipin IgM; CNS: central nervous system; cSLEDAI-2K: clinical Systemic Lupus Erythematosus Disease Activity Index 2000; DNA: anti-double stranded (ds)DNA; PFLC: polyclonal free light chains of kappa and lambda type; RF: rheumatoid factor; SM: anti-Smith; SSA52: anti-SSA/Ro52; SSA60: anti-SSA/Ro60; SSB: anti-SSB/La.

14 **Supplementary Table 13.** Correlations between positivity for selected serological markers and the cSLEDAI-2K vascular domain

Serological marker	n/N	Coefficient	*p* value
**B2G**	338/347	-0.09	0.116
**B2M**	338/347	-0.07	0.216
**C3C**	347/347	0.01	0.810
**C4**	347/347	0.02	0.704
**CHROMATIN**	312/347	-0.01	0.845
**CLG**	342/347	-0.09	0.108
**CLM**	342/347	-0.08	0.165
**DNA**	342/347	-0.01	0.911
**PFLC**	220/347	0.04	0.550
**RF**	346/347	0.15	**0.007**
**SM**	314/347	-0.05	0.344
**SSA52**	304/347	0.08	0.163
**SSA60**	305/347	0.05	0.403
**SSB**	315/347	0.04	0.428
**U1RNP**	315/347	-0.06	0.264

Phi (φ) correlations between autoantibody positivity or low levels of C3c or C4 and the cSLEDAI-2K vascular domain. In case of missing values, the total number of patients with available data is indicated. Statistically significant *p* values are in bold.

B2G: anti-β_2 glycoprotein I IgG; B2M: anti-β_2 glycoprotein I IgM; C3c: complement component 3c; C4: complement component 4CLG: anti-cardiolipin IgG; CLM: anti-cardiolipin IgM; cSLEDAI-2K: clinical Systemic Lupus Erythematosus Disease Activity Index 2000; DNA: anti-double stranded (ds)DNA; PFLC: polyclonal free light chains of kappa and lambda type; RF: rheumatoid factor; SM: anti-Smith; SSA52: anti-SSA/Ro52; SSA60: anti-SSA/Ro60; SSB: anti-SSB/La.

Supplementary Table 14. Correlations between positivity for selected serological markers and the cSLEDAI-2K musculoskeletal domain

Serological marker	n/N	Coefficient	*p* value
**B2G**	338/347	-0.09	0.089
**B2M**	338/347	-0.05	0.357
**C3C**	347/347	0.05	0.376
**C4**	347/347	0.04	0.470
**CHROMATIN**	312/347	0.06	0.276
**CLG**	342/347	-0.07	0.180
**CLM**	342/347	-0.07	0.185
**DNA**	342/347	0.06	0.287
**PFLC**	220/347	0.00	0.995
**RF**	346/347	0.06	0.276
**SM**	314/347	0.17	**0.002**
**SSA52**	304/347	0.06	0.289
**SSA60**	305/347	0.14	**0.014**
**SSB**	315/347	0.07	0.200
**U1RNP**	315/347	0.03	0.648

Phi (φ) correlations between autoantibody positivity or low levels of C3c or C4 and the cSLEDAI-2K musculoskeletal domain. In case of missing values, the total number of patients with available data is indicated. Statistically significant *p* values are in bold.

B2G: anti-β_2 glycoprotein I IgG; B2M: anti-β_2 glycoprotein I IgM; C3c: complement component 3c; C4: complement component 4; CLG: anti-cardiolipin IgG; CLM: anti-cardiolipin IgM; cSLEDAI-2K: clinical Systemic Lupus Erythematosus Disease Activity Index 2000; DNA: anti-double stranded (ds)DNA; PFLC: polyclonal free light chains of kappa and lambda type; RF: rheumatoid factor; SM: anti-Smith; SSA52: anti-SSA/Ro52; SSA60: anti-SSA/Ro60; SSB: anti-SSB/La.

Supplementary Table 15. Correlations between positivity for selected serological markers and the cSLEDAI-2K renal domain

Serological marker	n/N	Coefficient	*p* value
**B2G**	338/347	0.02	0.734
**B2M**	338/347	0.01	0.867
**C3C**	347/347	-0.01	0.900
**C4**	347/347	-0.03	0.562
**CHROMATIN**	312/347	0.19	**0.001**
**CLG**	342/347	0.01	0.878
**CLM**	342/347	-0.02	0.674
**DNA**	342/347	0.14	**0.008**
**PFLC**	220/347	0.04	0.524
**RF**	346/347	0.02	0.681
**SM**	314/347	0.05	0.425
**SSA52**	304/347	-0.01	0.835
**SSA60**	305/347	-0.03	0.644
**SSB**	315/347	-0.04	0.527
**U1RNP**	315/347	-0.03	0.611

Phi (φ) correlations between autoantibody positivity or low levels of C3c or C4 and the cSLEDAI-2K renal domain. In case of missing values, the total number of patients with available data is indicated. Statistically significant *p* values are in bold. B2G: anti-β_2 glycoprotein I IgG; B2M: anti-β_2 glycoprotein I IgM; C3c: complement component 3c; C4: complement component 4; CLG: anti-cardiolipin IgG; CLM: anti-cardiolipin IgM; cSLEDAI-2K: clinical Systemic Lupus Erythematosus Disease Activity Index 2000; DNA: anti-double stranded (ds)DNA; PFLC: polyclonal free light chains of kappa and lambda type; RF: rheumatoid factor; SM: anti-Smith; SSA52: anti-SSA/Ro52; SSA60: anti-SSA/Ro60; SSB: anti-SSB/La.

17 **Supplementary Table 16.** Correlations between positivity for selected serological markers and the cSLEDAI-2K mucocutaneous domain

Serological marker	n/N	Coefficient	*p* value
**B2G**	338/347	0.03	0.636
**B2M**	338/347	0.00	0.948
**C3C**	347/347	0.00	0.937
**C4**	347/347	0.05	0.352
**CHROMATIN**	312/347	0.15	**0.007**
**CLG**	342/347	0.04	0.420
**CLM**	342/347	-0.04	0.486
**DNA**	342/347	0.07	0.228
**PFLC**	220/347	0.16	**0.018**
**RF**	346/347	-0.01	0.900
**SM**	314/347	0.02	0.769
**SSA52**	304/347	-0.01	0.902
**SSA60**	305/347	0.03	0.564
**SSB**	315/347	0.09	0.111
**U1RNP**	315/347	0.08	0.164

Phi (φ) correlations between autoantibody positivity or low levels of C3c or C4 and the cSLEDAI-2K mucocutaneous domain. In case of missing values, the total number of patients with available data is indicated. Statistically significant *p* values are in bold.

B2G: anti-β_2 glycoprotein I IgG; B2M: anti-β_2 glycoprotein I IgM; C3c: complement component 3c; C4: complement component 4; CLG: anti-cardiolipin IgG; CLM: anti-cardiolipin IgM; cSLEDAI-2K: clinical Systemic Lupus Erythematosus Disease Activity Index 2000; DNA: anti-double stranded (ds)DNA; PFLC: polyclonal free light chains of kappa and lambda type; RF: rheumatoid factor; SM: anti-Smith; SSA52: anti-SSA/Ro52; SSA60: anti-SSA/Ro60; SSB: anti-SSB/La.

18 **Supplementary Table 17.** Correlations between positivity for selected serological markers and the cSLEDAI-2K serosal domain

**Serological marker**	**n/N**	**Coefficient**	***p* value**
**B2G**	338/347	0.01	0.887
**B2M**	338/347	0.03	0.549
**C3C**	347/347	-0.05	0.398
**C4**	347/347	-0.03	0.633
**CHROMATIN**	312/347	0.11	**0.043**
**CLG**	342/347	0.01	0.922
**CLM**	342/347	0.02	0.707
**DNA**	342/347	0.14	**0.010**
**PFLC**	220/347	0.14	**0.040**
**RF**	346/347	-0.04	0.457
**SM**	314/347	-0.03	0.581
**SSA52**	304/347	-0.02	0.763
**SSA60**	305/347	-0.03	0.581
**SSB**	315/347	-0.04	0.500
**U1RNP**	315/347	-0.02	0.716

Phi (φ) correlations between autoantibody positivity or low levels of C3c or C4 and the cSLEDAI-2K serosal domain. In case of missing values, the total number of patients with available data is indicated. Statistically significant *p* values are in bold.

B2G: anti-β_2 glycoprotein I IgG; B2M: anti-β_2 glycoprotein I IgM; C3c: complement component 3c; C4: complement component 4; CLG: anti-cardiolipin IgG; CLM: anti-cardiolipin IgM; cSLEDAI-2K: clinical Systemic Lupus Erythematosus Disease Activity Index 2000; DNA: anti-double stranded (ds)DNA; PFLC: polyclonal free light chains of kappa and lambda type; RF: rheumatoid factor; SM: anti-Smith; SSA52: anti-SSA/Ro52; SSA60: anti-SSA/Ro60; SSB: anti-SSB/La.

19 **Supplementary Table 18.** Correlations between positivity for selected serological markers and the cSLEDAI-2K constitutional domain

Serological marker	n/N	Coefficient	*p* value
**B2G**	338/347	-0.04	0.482
**B2M**	338/347	-0.03	0.579
**C3C**	347/347	0.08	0.145
**C4**	347/347	0.10	0.071
**CHROMATIN**	312/347	0.05	0.361
**CLG**	342/347	-0.04	0.472
**CLM**	342/347	-0.03	0.534
**DNA**	342/347	0.04	0.461
**PFLC**	220/347	-0.06	0.353
**RF**	346/347	-0.03	0.601
**SM**	314/347	-0.02	0.698
**SSA52**	304/347	0.03	0.625
**SSA60**	305/347	0.17	**0.004**
**SSB**	315/347	-0.03	0.635
**U1RNP**	315/347	0.02	0.665

Phi (φ) correlations between autoantibody positivity or low levels of C3c or C4 and the cSLEDAI-2K constitutional domain. In case of missing values, the total number of patients with available data is indicated. Statistically significant *p* values are in bold.

B2G: anti-β_2 glycoprotein I IgG; B2M: anti-β_2 glycoprotein I IgM; C3c: complement component 3c; C4: complement component 4; CLG: anti-cardiolipin IgG; CLM: anti-cardiolipin IgM; cSLEDAI-2K: clinical Systemic Lupus Erythematosus Disease Activity Index 2000; DNA: anti-double stranded (ds)DNA; PFLC: polyclonal free light chains of kappa and lambda type; RF: rheumatoid factor; SM: anti-Smith; SSA52: anti-SSA/Ro52; SSA60: anti-SSA/Ro60; SSB: anti-SSB/La.

20 **Supplementary Table 19.** Correlations between positivity for selected serological markers and the cSLEDAI-2K haematological domain

Serological marker	n/N	Coefficient	*p* value
**B2G**	338/347	-0.02	0.742
**B2M**	338/347	-0.03	0.590
**C3C**	347/347	0.07	0.177
**C4**	347/347	0.07	0.186
**CHROMATIN**	312/347	0.02	0.687
**CLG**	342/347	-0.02	0.647
**CLM**	342/347	-0.03	0.564
**DNA**	342/347	0.03	0.621
**PFLC**	220/347	-0.13	0.052
**RF**	346/347	0.10	0.057
**SM**	314/347	-0.07	0.193
**SSA52**	304/347	0.12	**0.037**
**SSA60**	305/347	0.14	**0.011**
**SSB**	315/347	0.05	0.355
**U1RNP**	315/347	-0.04	0.465

Phi (φ) correlations between autoantibody positivity or low levels of C3c or C4 and the cSLEDAI-2K haematological domain. In case of missing values, the total number of patients with available data is indicated. Statistically significant *p* values are in bold.

B2G: anti-β_2 glycoprotein I IgG; B2M: anti-β_2 glycoprotein I IgM; C3c: complement component 3c; C4: complement component 4; CLG: anti-cardiolipin IgG; CLM: anti-cardiolipin IgM; cSLEDAI-2K: clinical Systemic Lupus Erythematosus Disease Activity Index 2000; DNA: anti-double stranded (ds)DNA; PFLC: polyclonal free light chains of kappa and lambda type; RF: rheumatoid factor; SM: Smith; SSA52: anti-SSA/Ro52; SSA60: anti-SSA/Ro60; SSB: anti-SSB/La.

21 **Supplementary Table 20.** Correlations between positivity for selected serological markers and history of or current mucocutaneous manifestations

Serological marker	n/N	Coefficient	*p* value
**B2G**	249/253	0.04	0.503
**B2M**	249/253	-0.04	0.509
**C3C**	253/253	0.03	0.639
**C4**	253/253	0.02	0.769
**CHROMATIN**	229/253	0.06	0.357
**CLG**	253/253	0.08	0.201
**CLM**	253/253	-0.08	0.217
**DNA**	253/253	-0.03	0.644
**PFLC**	151/253	0.11	0.175
**RF**	252/253	0.06	0.336
**SM**	237/253	-0.01	0.823
**SSA52**	231/253	0.01	0.894
**SSA60**	232/253	-0.03	0.602
**SSB**	238/253	-0.03	0.660
**U1RNP**	238/253	0.06	0.333

Phi (φ) correlations between autoantibody positivity or low levels of C3c or C4 and any history of mucocutaneous manifestations. Mucocutaneous manifestations included malar rash, photosensitivity, acute cutaneous lupus, discoid lupus, chronic cutaneous lupus, mucosal ulcers, and alopecia. In case of missing values, the total number of patients with available data is indicated. Statistically significant *p* values are in bold.

B2G: anti-β_2 glycoprotein I IgG; B2M: anti-β_2 glycoprotein I IgM; C3c: complement component 3c; C4: complement component 4; CLG: anti-cardiolipin IgG; CLM: anti-cardiolipin IgM; CNS: central nervous system; DNA: anti-double stranded (ds)DNA; PFLC: polyclonal free light chains of kappa and lambda type; RF: rheumatoid factor; SM: Smith; SSA52: anti-SSA/Ro52; SSA60: anti-SSA/Ro60; SSB: anti-SSB/La.

Supplementary Table 21. Correlations between positivity for selected serological markers and history of or current arthritis

Serological marker	n/N	Coefficient	*p* value
**B2G**	249/253	-0.11	0.095
**B2M**	249/253	-0.03	0.630
**C3C**	253/253	0.03	0.674
**C4**	253/253	-0.02	0.756
**CHROMATIN**	229/253	-0.02	0.747
**CLG**	253/253	-0.06	0.346
**CLM**	253/253	-0.05	0.469
**DNA**	253/253	0.08	0.178
**PFLC**	151/253	0.03	0.697
**RF**	252/253	0.06	0.374
**SM**	237/253	0.02	0.804
**SSA52**	231/253	-0.01	0.901
**SSA60**	232/253	0.04	0.512
**SSB**	238/253	0.03	0.640
**U1RNP**	238/253	0.13	0.051

Phi (φ) correlations between autoantibody positivity or low levels of C3c or C4 and any history of arthritis. In case of missing values, the total number of patients with available data is indicated. Statistically significant *p* values are in bold. B2G: anti-β_2 glycoprotein I IgG; B2M: anti-β_2 glycoprotein I IgM; C3c: complement component 3c; C4: complement component 4; CLG: anti-cardiolipin IgG; CLM: anti-cardiolipin IgM; DNA: anti-double stranded (ds)DNA; PFLC: polyclonal free light chains of kappa and lambda type; RF: rheumatoid factor; SM: anti-Smith; SSA52: anti-SSA/Ro52; SSA60: anti-SSA/Ro60; SSB: anti-SSB/La.

Supplementary Table 22. Correlations between positivity for selected serological markers and history of or current serositis

Serological marker	n/N	Coefficient	*p* value
**B2G**	249/253	0.02	0.806
**B2M**	249/253	0.07	0.298
**C3C**	253/253	-0.10	0.125
**C4**	253/253	-0.05	0.385
**CHROMATIN**	229/253	0.17	**0.009**
**CLG**	253/253	0.03	0.603
**CLM**	253/253	0.03	0.628
**DNA**	253/253	0.18	**0.003**
**PFLC**	151/253	0.28	**<0.001**
**RF**	252/253	0.03	0.595
**SM**	237/253	-0.01	0.834
**SSA52**	231/253	0.12	0.072
**SSA60**	232/253	0.12	0.075
**SSB**	238/253	0.02	0.804
**U1RNP**	238/253	0.13	**0.039**

Phi (φ) correlations between autoantibody positivity or low levels of C3c or C4 and any history of serositis. In case of missing values, the total number of patients with available data is indicated. Statistically significant *p* values are in bold. B2G: anti-β_2 glycoprotein I IgG; B2M: anti-β_2 glycoprotein I IgM; C3c: complement component 3c; C4: complement component 4; CLG: anti-cardiolipin IgG; CLM: anti-cardiolipin IgM; DNA: anti-double stranded (ds)DNA; PFLC: polyclonal free light chains of kappa and lambda type; RF: rheumatoid factor; SM: anti-Smith; SSA52: anti-SSA/Ro52; SSA60: anti-SSA/Ro60; SSB: anti-SSB/La.

24 **Supplementary Table 23.** Correlations between positivity for selected serological markers and history of or current lupus nephritis

Serological marker	n/N	Coefficient	*p* value
**B2G**	249/253	0.01	0.928
**B2M**	249/253	0.00	0.975
**C3C**	253/253	0.00	0.962
**C4**	253/253	0.01	0.900
**CHROMATIN**	229/253	0.17	**0.010**
**CLG**	253/253	0.01	0.856
**CLM**	253/253	-0.01	0.854
**DNA**	253/253	0.12	0.062
**PFLC**	151/253	0.07	0.374
**RF**	252/253	-0.02	0.726
**SM**	237/253	0.08	0.237
**SSA52**	231/253	-0.06	0.347
**SSA60**	232/253	0.00	0.954
**SSB**	238/253	-0.05	0.461
**U1RNP**	238/253	-0.08	0.223

Phi (φ) correlations between autoantibody positivity or low levels of C3c or C4 and any history of lupus nephritis. In case of missing values, the total number of patients with available data is indicated. Statistically significant *p* values are in bold. B2G: anti-β_2 glycoprotein I IgG; B2M: anti-β_2 glycoprotein I IgM; C3c: complement component 3c; C4: complement component 4; CLG: anti-cardiolipin IgG; CLM: anti-cardiolipin IgM; DNA: anti-double stranded (ds)DNA; PFLC: polyclonal free light chains of kappa and lambda type; RF: rheumatoid factor; SM: anti-Smith; SSA52: anti-SSA/Ro52; SSA60: anti-SSA/Ro60; SSB: anti-SSB/La.

25 **Supplementary Table 24.** Correlations between positivity for selected serological markers and history of or current neuropsychiatric systemic lupus erythematosus

Serological marker	n/N	Coefficient	*p* value
**B2G**	249/253	-0.01	0.871
**B2M**	249/253	0.05	0.426
**C3C**	253/253	-0.04	0.557
**C4**	253/253	0.09	0.159
**CHROMATIN**	229/253	0.08	0.205
**CLG**	253/253	-0.02	0.790
**CLM**	253/253	0.07	0.256
**DNA**	253/253	-0.03	0.689
**PFLC**	151/253	0.08	0.350
**RF**	252/253	0.16	**0.010**
**SM**	237/253	-0.08	0.195
**SSA52**	231/253	0.01	0.829
**SSA60**	232/253	0.07	0.295
**SSB**	238/253	0.04	0.572
**U1RNP**	238/253	-0.04	0.588

Phi (φ) correlations between autoantibody positivity or low levels of C3c or C4 and any history of neuropsychiatric systemic lupus erythematosus. In case of missing values, the total number of patients with available data is indicated. Statistically significant *p* values are in bold.

B2G: anti-β_2 glycoprotein I IgG; B2M: anti-β_2 glycoprotein I IgM; C3c: complement component 3c; C4: complement component 4; CLG: anti-cardiolipin IgG; CLM: anti-cardiolipin IgM; DNA: anti-double stranded (ds)DNA; PFLC: polyclonal free light chains of kappa and lambda type; RF: rheumatoid factor; SM: anti-Smith; SSA52: anti-SSA/Ro52; SSA60: anti-SSA/Ro60; SSB: anti-SSB/La.

26 Supplementary Table 25. Correlations between positivity for selected serological markers and history of or current haematological manifestations

**Serological marker**	**n/N**	**Coefficient**	***p* value**
**B2G**	249/253	-0.06	0.372
**B2M**	249/253	-0.01	0.933
**C3C**	253/253	0.12	0.052
**C4**	253/253	0.17	**0.006**
**CHROMATIN**	229/253	0.10	0.128
**CLG**	253/253	-0.06	0.316
**CLM**	253/253	-0.03	0.638
**DNA**	253/253	0.14	**0.024**
**PFLC**	151/253	-0.05	0.513
**RF**	252/253	0.09	0.135
**SM**	237/253	0.06	0.377
**SSA52**	231/253	0.06	0.390
**SSA60**	232/253	0.13	**0.044**
**SSB**	238/253	0.15	**0.021**
**U1RNP**	238/253	-0.03	0.637

Phi (φ) correlations between autoantibody positivity or low levels of C3c or C4 and any history of haematological manifestations. Haematological manifestations included haemolytic anaemia, leukopenia, lymphopenia, and thrombocytopenia. In case of missing values, the total number of patients with available data is indicated. Statistically significant *p* values are in bold.

B2G: anti-β_2 glycoprotein I IgG; B2M: anti-β_2 glycoprotein I IgM; C3c: complement component 3c; C4: complement component 4; CLG: anti-cardiolipin IgG; CLM: anti-cardiolipin IgM; DNA: anti-double stranded (ds)DNA; PFLC: polyclonal free light chains of kappa and lambda type; RF: rheumatoid factor; SM: anti-Smith; SSA52: anti-SSA/Ro52; SSA60: anti-SSA/Ro60; SSB: anti-SSB/La.
